# Supplementary material for: Mothers in a cooperatively breeding bird increase investment per offspring at the pre-natal stage when they will have more help with post-natal care
Source: PLoS Biol. 2023 Nov 9;21(11):e3002356. doi: 10.1371/journal.pbio.3002356 (PMC10635431; doi:10.1371/journal.pbio.3002356)
Supplement: S1 File — Supporting information include the following: (A) Identification of the time windows of effect for temperature and rainfall. (B) Contextualizing the reported effect size of female helper number on egg volume. (C) No evidence of maternal adjustment of number of clutches laid per year according to helper numbers. (D) Maternal plasticity in egg volume cannot be readily attributed to carryover effects of past help. (E) No evidence of carry-over effects of past help on maternal body condition at laying. (F) Female helper effects on egg volume do not depend on time since the last breeding attempt or the number of helpers. (G) Variation in egg volume is better explained by “current” number of helpers than by number of helpers in the previous breeding attempt. (H) Bivariate models to estimate within- and among-female effects on egg volume and maternal provisioning rates. (I) Seeking evidence of a trade-off between egg volume and maternal provisioning rate. (J) Verifying maternal plasticity in egg size to the availability of female helpers when egg position data is missing. (PDF) [file pbio.3002356.s001.pdf]

## S1 File for:

# Mothers in a cooperatively breeding bird increase investment per offspring at the pre-natal stage when they will have more help with post-natal care

Pablo Capilla-Lasheras[1, 2 *], Alastair J. Wilson[1], Andrew J. Young[1 *]

[1] Centre for Ecology and Conservation, University of Exeter, Penryn, UK

[2] Current address: School of Biodiversity, One Health and Veterinary Medicine, University of Glasgow, Glasgow, UK

* Corresponding authors: Pablo Capilla-Lasheras (pacapilla@gmail.com), Andrew J. Young (A.J.Young@exeter.ac.uk)

**S1 File includes:**

**A - Identification of the time windows of effect for temperature and rainfall** 3

**B - Contextualising the reported effect size of female helper number on egg volume** ........ 7

**C – No evidence of maternal adjustment of number of clutches laid per year according to helper numbers** ........ 7

**D – Maternal plasticity in egg volume cannot be readily attributed to carry-over effects of past help** ........ 8

**E – No evidence of carry-over effects of past help on maternal body condition at laying** ........ 8

**F – Female helper effects on egg volume do not depend on time since the last breeding attempt or the number of helpers** ........ 9

**G – Variation in egg volume is better explained by 'current' number of helpers than by number of helpers in the previous breeding attempt** ........ 10

**H – Bivariate models to estimate within- and among-female effects on egg volume and maternal provisioning rates** ........ 10

**I – Seeking evidence of a trade-off between egg volume and maternal provisioning rate** ........ 13

**J – Verifying maternal plasticity in egg size to the availability of female helpers when egg position data is missing** ........ 14

**References** ........ 17

**A - Identification of the time windows of effect for temperature and rainfall**

*Rationale and methodology for the sliding window approach*

To control for the effects of variation in environmental temperature and rainfall on egg volume and maternal provisioning rate in the models presented in the main paper, we fitted two predictors within each model: a "heat waves" index (the total number of days within a specific time window prior to the focal event [laying or provisioning] in which the maximum daily temperature exceeded 35°C) and a rainfall index (the total amount of rainfall that fell within a specific time window prior to the focal event). The 'heat waves' index as defined here (i.e., number of days above 35°C) has been shown to appropriately capture hot-weather events in the Kalahari and it impacts the reproductive biology of several Kalahari bird species [1,2]. As the timing and duration of these specific time windows of effect were not known and could well differ between the two indices (heat waves and rainfall) and across the two response terms (egg volume and provisioning rate), prior to proceeding with the modelling exercise described in the main paper we used a sliding window approach to objectively identify these windows [3]. The sliding window approach described below was applied four times, to identify the best-supported time window of effect for each of the two indices when modelling each of the two responses. During this process we allowed for a linear effect of the heat wave index and both linear and quadratic effects of the rainfall index.

Within each application of the sliding window approach, we considered all possible temporal windows of >4 days in length, between an earliest start date of 80 days prior to egg laying and a latest end date of the day of egg laying. Only sliding windows of >4 days in length were considered, in order to decrease the likelihood of false positive results (which are more probable for very short windows). For each possible time window, the focal environmental index (heat waves or rainfall; see above) was calculated for all breeding attempts in the data set and then fitted as an additional predictor in a 'baseline model' for the focal response term (which was the full model described in the main paper for that response term, but lacking the heat waves and rainfall predictors). The level of statistical support for this time window was then calculated as the ΔAIC value between this model and the baseline model without this new predictor ('AIC support' below). The level of AIC support for all of the different possible windows for the focal environmental index were then ranked and the best-supported window carried forward for

use within the model described in the main paper if it improved the fit of the baseline model by >6 AIC points (a conservative threshold to avoid the accidental inclusion of uninformative terms [4]).

To assess the likelihood that this sliding window approach had yielded a false positive result for the best-supported window in each case, we carried out 25 randomisations of the data set [3]. In each randomisation, the 'biological reference date' (in this case the lay date of the focal clutch) in the data set was randomised by re-shuffling, similar to the approach implemented in the R package 'climwin' [5]. Following each randomisation of the data set, the full sliding window protocol described above was applied for the focal environmental variable, and the 'AIC support' for the model containing the best-supported window of effect for that environmental variable was recorded. The likelihood that the sliding window identified using the real (non-randomised) data set arose by chance (i.e., as a false positive) could then be estimated by calculating the proportion of the randomisations that yielded a best-supported window with stronger AIC support than that identified using the real data set.

*The time windows of effect identified for the heat wave and rainfall effects on egg volume*

The sliding window analysis for the effect of heat waves on egg volume identified a best-supported window that spanned the 13 days prior to egg laying (AIC support = -11.13), for which the heat wave index had a negative effect on egg volume (S2a Fig). The analysis identified a single and localised AIC peak in the sliding window landscape (S2b Fig) with no clear alternative windows supported by the data. None of our 25 randomisations yielded a best-supported window with equal or stronger AIC support than that identified using the real data, indicating that this result is unlikely to have arisen by chance (S2c Fig). Inclusion of this heat waves index as a predictor in the full model presented in the main paper led to a significant negative effect on egg volume (Table 1). High temperatures have been shown to negatively predict egg size in other species too [6–8]. It is conceivable that this pattern reflects an adaptive strategy (e.g., smaller eggs may be easier to keep cool) but could also reflect a detrimental effect of heat stress on pre-laying maternal condition or physiology [7,9].

The sliding window analysis for the effect of total rainfall on egg volume identified a best-supported window that spanned 44 to 49 days prior to egg laying (AIC support = -12.90), for which the rainfall index had a quadratic effect on egg volume (S3a Fig). This analysis revealed more scattered AIC

support across the sliding window landscape without such a clear single peak (S3b Fig), and three of our 25 randomisations yielded a best-supported window of effect with equal or stronger AIC support than that identified using the real data, indicating a 12% probability that this reflects a false positive result (S3c Fig). Inclusion of this total rainfall index as a predictor in the full model presented in the main paper led to a significant negative quadratic relationship between rainfall index and egg volume (Table 1). The shape of the quadratic rainfall relationship detected (S3a Fig) is consistent with the general expectation of a beneficial effect of rainfall on resource availability in this arid environment, but costs associated with very high rainfall events (that can damage the birds' woven structures and flood the landscape).

Additional analyses confirmed that the female helper number effects on egg volume detected in the main text have not been determined by including the sparser data for higher rainfall values or the resulting quadratic rainfall fit. When we just fit a linear rainfall relationship to the egg volume data from below the peak in the detected quadratic rainfall relationship (i.e., for rainfall levels < 60mm; see S3a Fig), where the data density is higher, the effect sizes for the effects of both female helper number (i.e., prior to partitioning) and Δ female helper number (following partitioning) on egg volume are virtually unchanged: (i) Female helper number effect size ± SE with full data set and quadratic rainfall fit (as per Table 1 in the manuscript) = 0.018 ± 0.009 $cm^3$ / female helper, and then for rainfall < 60mm and linear rainfall fit = 0.019 ± 0.009 $cm^3$ / female helper (the associated p-value for female helper number drops slightly from 0.038 to 0.026; S12 Table). (ii) Δ female helper number effect size ± SE with full data set and quadratic rainfall fit (as per Table 2) = 0.019 ± 0.009; and then for rainfall < 60mm and linear rainfall fit = 0.019 ± 0.009 $cm^3$ / female helper (the associated p-value for female helper number drops slightly from 0.037 to 0.035; S13 Table). Not including rainfall effects in the egg volume models leaves the effect size estimates for both female helper number (i.e., prior to partitioning) and Δ female helper number (following partitioning) virtually unchanged: (i) Female helper number effect size ± SE with rainfall index included (as per Table 1) = 0.018 ± 0.009 cm3 / female helper, and without rainfall included = 0.017 ± 0.009 cm3 / female helper (the associated p-value rises slightly from 0.038 to 0.063; S14 Table). (ii) Δ female helper number effect size ± SE with rainfall included (as per Table 2) = 0.019 ± 0.0090 cm3 / female helper, and without rainfall included = 0.017 ± 0.009 cm3 / female helper (the associated p-value rises slightly from 0.037 to 0.066; S15 Table).

*The time windows of effect identified for the heat wave and rainfall effects on maternal provisioning rate*

The sliding window analysis for the effect of heat waves on maternal provisioning rate identified a best-supported window that spanned 51-59 days prior to egg laying (AIC support = -10.01), for which the heat wave index had a positive effect on maternal provisioning rate (S4a Fig). The analysis revealed AIC support that was widely distributed across the sliding window landscape (S4b Fig), but none of our 25 randomizations yielded a best-supported window of effect with equal or stronger AIC support than that identified using the real data (S4c Fig). Inclusion of this heat waves index as a predictor in the full model presented in the main paper led to a significant positive effect on maternal provisioning rate (Table 3). Our inferences regarding the effects of female and male helper number on maternal provisioning rate remain unchanged, however, if this heat waves predictor is excluded from the model in the main paper.

The sliding window analysis for the effect of total rainfall on maternal provisioning rate identified a best-supported window that spanned 61-78 days prior to egg laying (AIC support = -13.08), for which total rainfall had a positive quadratic effect on maternal provisioning rate (S5a Fig). The analysis revealed a clear peak of AIC support in the sliding window landscape (S5b Fig). Four of our 25 randomizations yielded a best-supported window of effect with equal or stronger AIC support than that identified using the real data, indicating a 16% probability that this reflects a false positive result (S5c Fig). Inclusion of this total rainfall index as a predictor in the full model presented in the main paper led to a significant positive quadratic relationship between rainfall index and maternal provisioning rate (Table 3). The quadratic rainfall relationship detected (S5a Fig) is suggestive of an accelerating positive effect of increasing rainfall on maternal provisioning rate, consistent again with beneficial effects of rainfall on resource availability in this arid environment. Not including rainfall effects in models for maternal provisioning rates leaves the effect size estimates for both female helper number and Δ female helper number virtually unchanged: (i) Female helper number effect size with rainfall included = -0.457 ± 0.195 feeds / hour / female helper (as per Table 3), and without rainfall included = -0.454 ± 0.202 feeds / hour / female helper (the associated p-value rises slightly from 0.020 to 0.026; S16 Table). (ii) Δ female helper number effect size with rainfall included = -0.559 ± 0.269 feeds / hour / female helper (as per

Table 4), and without rainfall included = -0.530 ± 0.279 feeds / hour / female helper (the associated p-value rises slightly from 0.040 to 0.060; S17 Table).

**B - Contextualising the reported effect size of female helper number on egg volume**

While the effect size for our maternal plastic response in egg volume is modest, it aligns well with the effect sizes for the population-level relationships between helper number and egg size that are already the focus of research in this field. For example, Figure 2a in Russell et al., (2007), the landmark paper that stimulated research in this area, suggests that the effect size in that study for the contrast in egg volume between mothers with and mothers without helpers was approximately -0.067 $cm^3$ of egg volume (-5.3% of mean egg volume), which is broadly comparable in both absolute and proportional magnitude to the within-mother (i.e., plastic) effect size that we report here; for six female helpers (the natural range in female helper number in our analysis) the change in egg volume would be 6 × 0.019 $cm^3$ (see Table 2) = +0.114 $cm^3$ of egg volume (+3.3% of mean egg volume). As such, while it is reasonable to consider the likely biological significance of these effect sizes (see Discussion in the main text), the magnitude of the effect sizes in our study is not unusual.

**C – No evidence of maternal adjustment of number of clutches laid per year according to helper numbers**

We found no evidence that mothers adjusted the total number of clutches that they laid per breeding season according to the average number of female or male helpers that they had in their group over the course of the breeding season. Analysis at the population level revealed that the number of clutches that a mother laid per breeding season was not significantly predicted by either the number of female helpers (female helper number effect ± SE = 0.054 ± 0.041 clutches / female helper, $\chi^2_1 = 1.74$, p = 0.187; S18 Table) or the number of male helpers (male helper number effect ± SE = 0.087 ± 0.048 clutches / male helper, $\chi^2_1 = 3.23$, p = 0.072; S18 Table). We found similar results after partitioning variation in helper numbers into their within- and among-mother components; the number of clutches laid was not significantly predicted by within-mother variation in either female helper number (Δ female helper number effect ± SE = 0.010 ± 0.054 clutches / female helper; $\chi^2_1 = 0.03$, p = 0.855; S19 Table)

or male helper number (Δ male helper number effect ± SE = -0.054 ± 0.061 clutches / male helper; $\chi^2_1$ = 0.77, p = 0.379; S19 Table).

**D – Maternal plasticity in egg volume cannot be readily attributed to carry-over effects of past help**

The apparent maternal plasticity in egg size according to female helper number detected in the main paper could conceivably arise not because mothers *pre-emptively* adjust egg investment to the expected level of post-natal helping that a clutch will receive, but because the *past* actions of helpers in a previous breeding attempt have impacted maternal condition at laying (e.g., via lightening maternal workloads [11–13]). This alternative scenario cannot readily explain our findings, however, as (i) maternal body condition before laying is not predicted by the helper numbers that she had during her previous breeding attempt (see Supplementary materials E, below); (ii) the time since the last breeding attempt does not predict egg volume (either in isolation or via interactions with current helper numbers), suggesting that egg volume is not appreciably impacted by carry-over effects of past reproductive effort (see Supplementary materials F, below); and, the number of helpers in the previous breeding attempt does not predict egg volume (either in isolation or via interactions with the time since the last breeding attempt) (see Supplementary materials G, below). As such, it seems more likely that the egg size plasticity observed does reflect maternal adjustment of pre-natal investment according to the likely future availability of post-natal help [10,14], which is highly predictable at the time of laying (S1 Fig).

**E – No evidence of carry-over effects of past help on maternal body condition at laying**

We investigated whether maternal body condition before laying a given clutch was predicted by the numbers of helpers present during the rearing of her previous clutch. We restricted the analysis to those ‘previous’ clutches in which at least one nestling fledged, in order to ensure that mothers had had to engage in post-natal care during the previous breeding attempt and that helpers had had the opportunity to lighten the maternal post-natal workload. We routinely measured body mass (g) and tarsus length (mm) of mothers caught throughout the study period. We used mass measurements from mothers up to 45 days before they produced a new focal clutch (i.e., pre-laying). The data set comprised 45 focal clutches laid by 33 mothers in 26 social groups. We calculated the scale mass index of body condition (hereafter ‘maternal pre-laying body condition’) following [15], and built a linear mixed model to explain

variation in this variable. We investigated the effects of female and male helper number in the previous breeding attempt (as fixed effects) on maternal pre-laying body condition. We also included a fixed effect for the number of previous clutches laid by the mother during that breeding season. Mother ID, social group ID and breeding season ID were included as random effect intercepts. We found no evidence that maternal pre-laying body condition is predicted by the number of female helpers (effect of female helper number on maternal pre-laying condition ± SE = -0.232 ± 0.350 g / female helper, $\chi^2_1$ = 0.36, p = 0.551) or male helpers (effect of male helper number on maternal pre-laying condition ± SE = 0.108 ± 0.404 g / male helper, $\chi^2_1$ = 0.03, p = 0.862) that she had in her previous breeding attempt (S20 Table).

**F – Female helper effects on egg volume do not depend on time since the last breeding attempt or the number of helpers**

If mothers laid larger eggs when assisted by more female helpers (the relationship observed in the main paper) because female helper contributions lightened the mothers' post-natal workload within the *previous* breeding attempt, we would expect the positive effect of female helper number on egg volume to decrease in magnitude with increasing time since the last breeding attempt (i.e., an interaction between female helper number and time since last breeding attempt within the egg volume model). To investigate whether this was the case, we re-fitted the egg volume model presented in the main text to include 'time since last breeding attempt' (range 36-408 days; mean = 86.68 days) as an additional fixed effect predictor. We restricted the data set to only include the egg volumes of those breeding attempts for which the previous breeding attempt had fledged at least one nestling, in order to ensure that mothers had had to engage in post-natal care during the previous breeding attempt and that helpers had had the opportunity to lighten the maternal post-natal workload (n = 136 eggs from 79 clutches laid by 40 mothers in 32 groups). Time since the last breeding attempt did not explain variation in egg volume, either as an interaction with female ($\chi^2_1$ = 0.01, p = 0.909) or male helper number ($\chi^2_1$ = 0.02, p = 0.875) or as a simple predictor (effect size ± SE = 0.000 ± 0.000 $cm^3$ / day elapsed, $\chi^2_1$ = 0.37, p = 0.544; S21 Table).

**G – Variation in egg volume is better explained by 'current' number of helpers than by number of helpers in the previous breeding attempt**

If mothers laid larger eggs when assisted by more female helpers (the relationship observed in the main paper) because female helper contributions lightened the mothers' post-natal workload within the *previous* breeding attempt, we would expect that the number of helpers in the previous breeding attempt explains variation in egg volume better than the number of helpers in the current breeding event. To test this hypothesis, we used AIC to compare (a) the egg volume model presented in the main text including 'time since last breeding' as an additional fixed effect predictor in isolation and in interaction with 'current breeding attempt' male and female helper number (this is the model presented in Section F, above), and (b) an identical model replacing 'current breeding attempt' male and female helper numbers by 'previous breeding attempt' male and female helper numbers. Both, previous and current helper number variables were not included in the same model due to their high correlation (Pearson's product-moment correlation [95% CI] = 0.79 [0.71, 0.85] for previous and current helper number, for both male and female helpers). However, if helper effects on egg volume were a carry-over effect of past help, we would expect the 'previous breeding attempt' model to outperform (i.e., have lower AIC) the 'current breeding attempt' model. AIC model comparison provides an ideal framework for this test as it allows statistical comparison of non-nested models. Again, we restricted the dataset to only include the egg volumes of those breeding attempts for which the previous breeding attempt had fledged at least one nestling, in order to ensure that mothers had had to engage in post-natal care during the previous breeding attempt and that helpers had had the opportunity to lighten the maternal post-natal workload (n = 113 eggs from 66 clutches laid by 38 mothers in 32 groups with information for current and previous breeding attempt helper numbers). The 'previous breeding attempt' model (AIC = 1575.35) did not outperform the 'current breeding attempt' model (AIC = 1574.87). In fact, it fitted the data slightly worse than the 'current breeding attempt' model providing no evidence that helper effects on egg volume could be mediated by carry-over effects of past help.

**H – Bivariate models to estimate within- and among-female effects on egg volume and maternal provisioning rates**

In order to confirm the existence of a plastic maternal reaction norm to female helper numbers in the main paper, we used a partitioning approach that is expected to yield unbiased estimates for such

within-individual effects [16]. We also confirmed that a second, more data-demanding, method for estimating within-individual effects (a bivariate modelling approach) yielded similar effect size estimates for the within-mother reaction norms to female helper number, both within the egg volume analysis and the provisioning rate analysis. We present the methods and outcomes of these models below.

*Bivariate model for egg volume*

We followed the model specification described in the main text for the egg volume model, but included female helper number as a second response term. The fixed effect predictors for the egg volume response were identical to those in the model presented in the main paper Table 1, except that the female helper number term was omitted (as this was now fitted as a second response term). No fixed effect predictors were included for the female helper number response term; this was set up as an intercept-only model. We included group ID, season ID and mother ID variance and covariance terms for each response term. The model also estimated residual variance and covariance. Both responses were assumed to follow Gaussian distributions [17]. This model was fitted in R using the MCMCglmm package (v2.34; [18]), using an inverse Gamma as prior distribution for random and residual (co)variances (V = 1, nu = 1.002), 50000 MCMC iterations, with 1000 as initial burn-in and sampling every 10 iterations. Effective sample sizes for all model terms were always higher than 4000 and MCMC traces were visually inspected to confirm convergence. We calculated both the within- and among-mother slopes for the relationship between egg volume and female helper number by dividing the corresponding covariance between egg volume and female helper number by the estimated variance in female helper number. The resulting estimate for the within-mother slope of egg volume on female helper number (estimate [95% Credible Interval] = +0.015 [-0.004, 0.033] $cm^3$ / female helper; S22 Table) closely matched the magnitude of the within-mother slope (± SE) estimated in the main paper analyses via the partitioning method (+0.019 ± 0.009 $cm^3$ / female helper; Table 2); as expected given [16]. While the Credible Interval for the bivariate estimate of the within-mother slope is principally positive, it does span zero at its limit, which is to be expected given the data-demanding nature of the bivariate approach and the already modest statistical significance of the maternal reaction norm when using the partitioning approach ($p = 0.037$; see Table 2). The bivariate approach also estimated the among-mother slope for egg volume on female helper number (estimate [95% CrI] = +0.041 [-0.126, 0.214] $cm^3$ / female helper; S22 Table), and revealed no evidence that this differed significantly from

the within-mother slope (estimated difference between the within- and among-mother slopes [95% CrI] = -0.024 [-0.183, 0.151]).

*Bivariate model for maternal provisioning rate*

We followed the model specification described in the main text for the maternal provisioning rate model, but included female helper number as a second response term. The fixed effect predictors for the maternal provisioning rate response were identical to those in the model presented in the main paper Table 3, except that the female helper number term was omitted (as this was now fitted as a second response term). No fixed effect predictors were included for the female helper number response term; this was set up as an intercept-only model. We included group ID, season ID and mother ID variance and covariance terms for each response term. The model also estimated residual variance and covariance. Both responses were assumed to follow Gaussian distributions [17]. This model was fitted in R using the MCMCglmm package (v2.34; [18]), using an inverse Gamma as prior distribution for random and residual (co)variances (V = 1, nu = 1.002), 50000 MCMC iterations, with 1000 as initial burn-in and sampling every 10 iterations. Effective sample sizes for all model terms were always higher than 2500 and MCMC traces were visually inspected to confirm convergence. We calculated both the within- and among-mother slopes for the relationship between female helper number and maternal provisioning rate by dividing the corresponding covariance between maternal provisioning rate and female helper number by the estimated variance in female helper number. The resulting estimate for the within-mother slope of maternal provisioning rate on female helper number (estimate [95% Credible Interval] = -0.451 [-1.077, 0.151] feeds / hour / female helper; S23 Table) closely matched the magnitude of the within-mother slope (± SE) estimated in the main paper analyses via the partitioning method (-0.56 ± 0.27 feeds / hour / female helper; Table 4); as expected given [16]. While the Credible Interval for the bivariate estimate of the within-mother slope is principally negative, it does span zero at its limit, which is to be expected given the data-demanding nature of the bivariate approach and the already modest statistical significance of the maternal reaction norm when using the partitioning approach ($p = 0.040$; Table 4). The bivariate approach also estimated the among-mother slope for maternal provisioning rate on female helper number (estimate [95% CrI] = -0.311 [-1.381, 0.667] feeds / hour / female helper; S23 Table), and revealed no evidence that this differed significantly from the within-

mother slope (estimated difference between the within- and among-mother slopes [95% CrI] = -0.141 [-1.370, 1.214]).

**I – Seeking evidence of a trade-off between egg volume and maternal provisioning rate**

We fitted additional models to investigate whether there is evidence of negative within-mother covariance between egg volume and maternal provisioning rate, indicative of a trade-off between pre- and post-natal maternal investment. To do this we followed an approach similar to the hybrid approach suggested in [19]. In short, we fitted a bivariate model including egg volume and maternal provisioning rate as responses (both mean-centered and scaled, and assuming they follow a normal distribution). For both responses, we used the fixed effect structure that we specify in the main text and show in Table 1 (for egg volume) and Table 3 (for maternal provisioning rate), with all predictor variables mean-centered and scaled by one standard deviation to improve model convergence. The random effect structure of this bivariate model included group ID, season ID, mother ID and clutch ID variance terms for each response term as well as mother ID and clutch ID covariance terms. The model also estimated residual variance for egg volume. Residual variance for maternal feeding rate was fixed at 0 as the dataset contained only one observation per clutch ID. Therefore, the clutch ID covariance term captures covariation between egg volume and provisioning rate within mothers. This analysis used the full data set from the egg volume model in Table 1 (490 egg volume measures from 271 clutches) and the full data set from the maternal provisioning rate model in Table 3 (124 maternal provisioning rate measures from 124 broods). 336 clutches/broods in total, where 59 clutches/broods had measures for both egg volume and provisioning rate, while 277 clutches/broods had measures for only one of these two metrics.

Following [19], we fitted this model in two ways. First, we fitted it without female and male helper numbers as fixed effects for either response term, to test for evidence of negative within-mother covariance between the two maternal traits (indicative of a trade-off between pre- and post-natal maternal investment). Second, we fitted it with female and male helper numbers included as fixed effects for both responses, to establish whether any negative within-mother covariance evident in the first model became weaker on including these helper number terms (which would suggest that mothers adjust their resolution of such a trade-off [i.e., their position along the trade-off relationship] according

to helper numbers). These models were fitted in R using the brms package (v2.34; [20]), using a default prior distribution for random variance components and a normal distribution of mean 0 and standard deviation 100 for fixed effects, and four chains of 50000 MCMC iterations, with 25000 as initial burn-in and sampling every 10 iterations in each case. Model convergence was assessed via Rhat values (which were always below 1.01) and visual inspection of chain traces.

The first model (without helper numbers as fixed effects) revealed no evidence of a trade-off between maternal pre- and post-natal investment; the within-mother covariance between egg volume and maternal provisioning rate was estimated to be 0.018 (95% CrI = -0.058, 0.097; S24 Table). The second model (including helper numbers as fixed effects) was rendered somewhat redundant by the lack of an evident trade-off in the first model. It also revealed no evidence of such a trade-off (within-mother covariance = 0.032 [95% CrI = -0.042, 0.112]; S25 Table), while capturing the positive effect of female helper number on egg volume (model estimate [95% CrI] = 0.070 [0.003, 0.137]; S25 Table) and the negative effect of female helper number on maternal provisioning rate (model estimate [95% CrI] = -0.183 [-0.337,-0.027]; S25 Table).

**J – Verifying maternal plasticity in egg size to the availability of female helpers when egg position data is missing**

Egg position information was available for 490 eggs whose data we used in the analyses presented in the main text. Clutch size data was also highly accurate for these eggs as our measures of laid clutch size were more accurate for clutches with laying order data. This is because egg position information and accurate laid clutch size information both stem from conducting daily nest checks throughout the laying sequence (i.e., the longer the time interval between nest checks, the less likely it was that we had accurate laying order data, and the higher the probability that we missed laid eggs that were subsequently lost from the clutch before our next nest check). Using egg data with accurate egg position and clutch size information was a priority as this information allowed us to test for potentially important interactions between egg position and the focal helper effects of interest as well as to control for potential main effects of both egg position and clutch size on egg volume ([21]; indeed, egg position proved to be the strongest predictor of egg volume; see results).

Nevertheless, having found evidence for maternal plasticity in egg volume accordingly to female helper numbers when using this data set (see Results), we confirmed that evidence of maternal plasticity in egg volume was still apparent when conducting an 'extended analysis' using the full data set (n = 906 eggs; see methods), despite the inability to fit egg position as a predictor when using this data (as egg position was unknown for 416 of these eggs). To do this, we repeated the whole sliding window approach with the extended dataset and recovered the same time windows of relevance for rainfall and heat wave index. Then, we re-ran the full model partitioning female and male helper number into their within- and among-mother components following the same structure of the model presented in the main text, but lacking the egg position predictor. Briefly, this extended analysis model included rainfall index (linear and quadratic terms), heat wave index, clutch size (as a continuous variable), Δ and μ female and male helper numbers, and the interactions between within- and among-mother helper numbers and clutch size. The random effect part of the model included mother ID, clutch ID, group ID and breeding season.

This extended analysis model yielded an effect size estimate for the effect of within-mother variation in female helper number on egg volume of +0.010 ± 0.009 $cm^3$ / female helper (S26 Table; for reference, the effect reported in the main text is +0.019 ± 0.009 $cm^3$ / female helper; Table 2). Exploring the origin of this reduction in the overall effect size estimate provided strong evidence for a positive effect of within-mother variation in female helper number on egg volume in clutches of one egg (effect size = +0.131 ± 0.019 $cm^3$ / female helper, $\chi^2_1$ = 20.89, p < 0.001; S27 Table) and clutches of three eggs (effect size = +0.058 ± 0.020 $cm^3$ / female helper, $\chi^2_1$ = 7.26, p = 0.007; S28 Table); but no evidence of an effect in clutches of two eggs (effect size = -0.002 ± 0.010 $cm^3$ / female helper, $\chi^2_1$ = 0.03, p = 0.855; S29 Table). These contrasting effects of within-mother variation in female helper number across clutches of different sizes were reflected as a significant interaction between clutch size (as a categorical variable) and within-mother variation in female helper number ($\chi^2_2$ = 6.06, p = 0.048; S30 Table). As such, analysis of the expanded data set also provides statistical support for maternal plasticity in egg volume according to female helper numbers, and highlights the possibility that the positive maternal reaction norm of egg volume to female helper number is also context dependent: larger in magnitude than the effect reported in our main analyses in some clutch sizes and not discriminable from zero in others. Whether this interaction reflects biological reality is unclear, however,

as this extended analysis cannot account for the demonstrably important effect of egg position on egg volume (see Results). It is also difficult to interpret this interaction, as the uncontrolled variation in egg position within this expanded data set will also confound variation in clutch size (e.g., because third-laid eggs only occur in three-egg clutches), leaving it unclear whether any such interaction is driven principally by variation in clutch size or egg position effects.

As in the main paper analyses, the positive maternal reaction norm in egg volume was only true for female helper numbers (in no contexts was a positive response to male helpers evident), underscoring the interpretation within the main paper that mothers are mounting their positive plastic response in egg volume to the availability of help *per se*, rather than simply variation in group size (because only female helpers have a net positive [i.e., additive] effect on the rate at which nestlings are fed [22] and only female helpers significantly lighten the workloads of mothers; Tables 3 and 4). The extended analysis also highlights that the maternal response to male helper numbers may become significantly negative under some contexts, as male helper number also interacted with clutch size (S30 Table). This result should also be treated with caution, given inherent weaknesses in this extended analysis (see above) and because the more reliable main paper analyses revealed no evidence of such a negative response to male helper numbers (Tables 1 and 2). If mothers actually were reducing their egg volume in response to male helper numbers in some contexts, this reduction would be unlikely to reflect a response to help (as we never observe such a negative response to female helper numbers) and so any adaptive explanation for it would likely lie outside the focal hypotheses of this paper. One possible explanation is that dominant females may suffer net costs from having too many male helpers on their territories (due, for example, to foraging competition), which may constrain their ability to invest in the egg. This would be consistent with our recent finding that dominant females suffer reduced survival when there are more male helpers in their group, but enjoy improved survival when they have more female helpers [23]; perhaps because female helpers compensate for their presence by lightening maternal workloads; Tables 3 and 4).

**References**


1. Cunningham SJ, Martin RO, Hojem CL, Hockey PAR. Temperatures in excess of critical thresholds threaten nestling growth and survival in a rapidly-warming arid savanna: a study of common fiscals. PLoS One. 2013;8: e74613. doi:10.1371/journal.pone.0074613

2. Cunningham SJ, Kruger AC, Nxumalo MP, Hockey PAR. Identifying biologically meaningful hot-weather events using threshold temperatures that affect life-history. PLoS One. 2013;8: e82492. doi:10.1371/journal.pone.0082492

3. van de Pol M, Bailey LD, McLean N, Rijsdijk L, Lawson CR, Brouwer L. Identifying the best climatic predictors in ecology and evolution. Methods Ecol Evol. 2016;7: 1246–1257. doi:10.1111/2041-210X.12590

4. Richards SA. Dealing with overdispersed count data in applied ecology. Journal of Applied Ecology. 2008;45: 218–227. doi:10.1111/j.1365-2664.2007.01377.x

5. Bailey LD, van de Pol M. Climwin: An R toolbox for climate window analysis. PLoS One. 2016;11: e0167980. doi:10.1371/journal.pone.0167980

6. Bennion NL, Warren DC. Temperature and its effect on egg size in the domestic fowl. Poult Sci. 1933;XII: 69–82. doi:10.3382/ps.0120069

7. Blanckenhorn WU. Temperature effects on egg size and their fitness consequences in the yellow dung fly Scathophaga stercoraria. Evol Ecol. 2000;14: 627–643. doi:10.1023/A:1010911017700

8. Langmore NE, Bailey LD, Heinsohn RG, Russell AF, Kilner RM. Egg size investment in superb fairy-wrens: Helper effects are modulated by climate. Proceedings of the Royal Society B. 2016;283: 20161875. doi:10.1098/rspb.2016.1875

9. Ernsting G, Isaaks JA, Universiteit V. Effects of temperature and season on egg size, hatchling size and adult size in Notiophilus biguttatus. Ecol Entomol. 1997;22: 32–40. doi:10.1046/j.1365-2311.1997.00040.x

10. Russell AF, Langmore NE, Cockburn A, Astheimer LB, Kilner RM. Reduced egg investment can conceal helper effects in cooperatively breeding birds. Science. 2007;317: 941–944.

11. Hatchwell BJ. Investment Strategies of Breeders in Avian Cooperative Breeding Systems. American Naturalist. 1999;154: 205–219. doi:10.1086/303227

12. Zöttl M, Fischer S, Taborsky M. Partial brood care compensation by female breeders in response to experimental manipulation of alloparental care. Anim Behav. 2013;85: 1471–1478. doi:10.1016/j.anbehav.2013.03.045

13. Brouwer L, van de Pol M, Cockburn A. The role of social environment on parental care: Offspring benefit more from the presence of female than male helpers. Journal of Animal Ecology. 2014;83: 491–503. doi:10.1111/1365-2656.12143

14. Russell AF, Lummaa V. Maternal effects in cooperative breeders: From hymenopterans to humans. Philosophical Transactions of the Royal Society B. 2009;364: 1143–1167. doi:10.1098/rstb.2008.0298

15. Peig J, Green AJ, Ame C. New perspectives for estimating body condition from mass / length data : the scaled mass index as an alternative method. Oikos. 2009;118: 1883–1891. doi:10.1111/j.1600-0706.2009.17643.x

16. Westneat DF, Araya-Ajoy YG, Allegue H, Class B, Dingemanse N, Dochtermann N, et al. Collision between biological process and statistical analysis revealed by mean-centering. Journal of Animal Ecology. 2020;89: 2813–2824. doi:10.1111/1365-2656.13360

17. Schielzeth H, Dingemanse NJ, Nakagawa S, Westneat DF, Allegue H, Teplitsky C, et al. Robustness of linear mixed-effects models to violations of distributional assumptions. Methods Ecol Evol. 2020;11: 1141–1152. doi:10.1111/2041-210x.13434

18. Hadfield JD. MCMC Methods for Multi-Response Generalized Linear Mixed Models in R: The MCMCglmm R package. J Stat Softw. 2010;33: 1–22. doi:10.1002/ana.22635

19. McAdam AG, Garant D, Wilson AJ. The effects of others' genes: maternal and other indirect genetic effects. In: Charmantier A, Darant dany, Kruuk LE, editors. Quantitative Genetics in the Wild. Oxford: Oxford University Press; 2014. pp. 84–103. Available: https://academic.oup.com/book/27921/chapter/204009906

20. Bürkner P-C. brms: An R Package for Bayesian Multilevel Models Using Stan. J Stat Softw. 2017;10: 1–28. doi:10.18637/jss.v080.i01

21. Fortuna R, Paquet M, Biard C, Élard L, Ferreira AC, Leroux-Coyaux M, et al. Egg components and offspring survival vary with group size and laying order in a cooperative breeder. Oecologia. 2023. doi:10.1007/s00442-023-05379-w

22. Capilla-Lasheras P, Harrison X, Wood EM, Wilson AJ, Young AJ. Altruistic bet hedging and the evolution of cooperation in a Kalahari bird. Sci Adv. 2021;7: eabe8980.

23. O'Callaghan O. Social effects on adult survival in a wild cooperative bird. Msc Thesis. University of Exeter. 2021.
