# Supplementary figures and images for: Mothers in a cooperatively breeding bird increase investment per offspring at the pre-natal stage when they will have more help with post-natal care
Source: PLoS Biol. 2023 Nov 9;21(11):e3002356. doi: 10.1371/journal.pbio.3002356 (PMC10635431; doi:10.1371/journal.pbio.3002356)

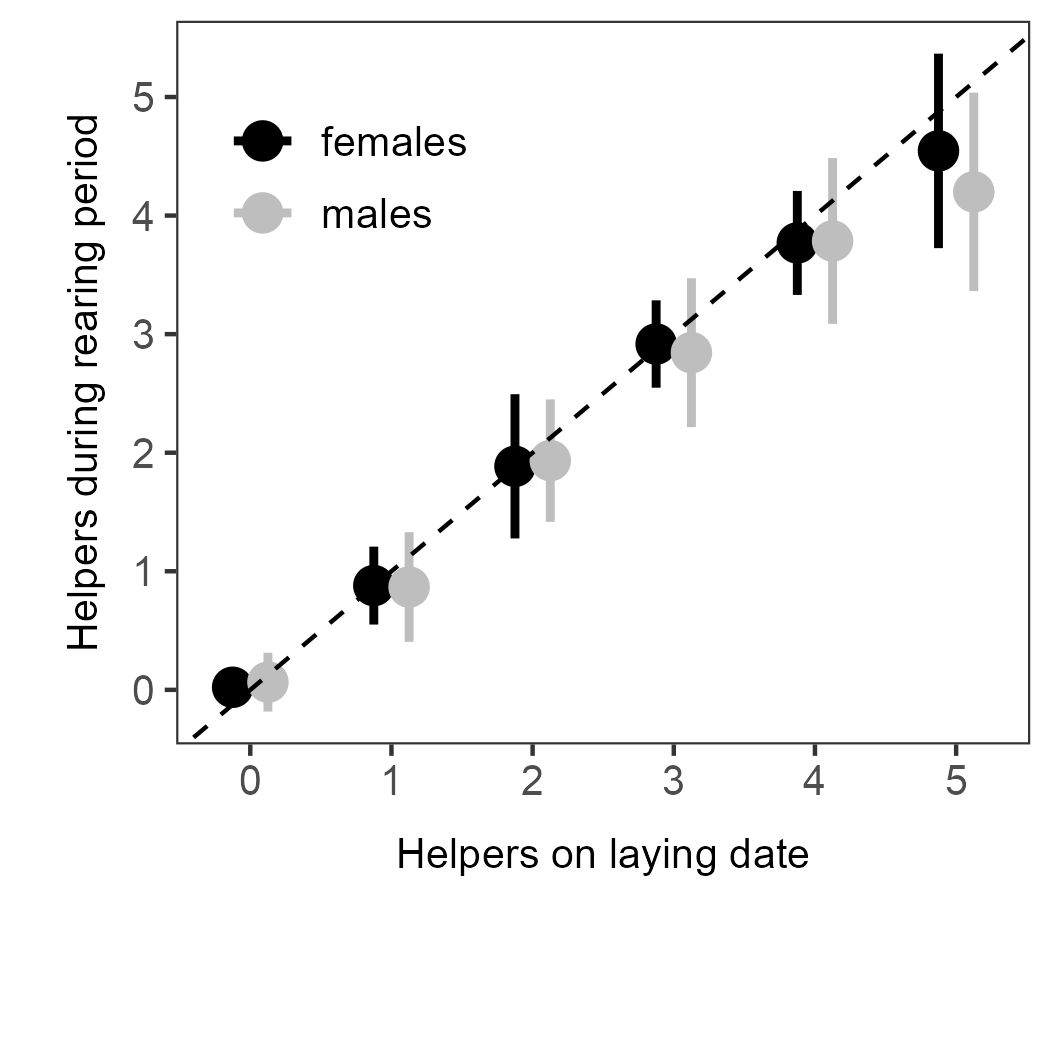

Supplement: S1 Fig — Mean ± standard deviation (SD) is presented for both male and female helper numbers (dashed line indicates a 1:1 relationship). For female helper number, linear model: N = 271 breeding attempts, β = 0.94 ± 0.017. For male helper number, linear model: N = 271 breeding attempts, β = 0.93 ± 0.022). The data underlying this figure can be found at https://doi.org/10.5281/zenodo.8385995. (TIF) [file pbio.3002356.s002.tif]

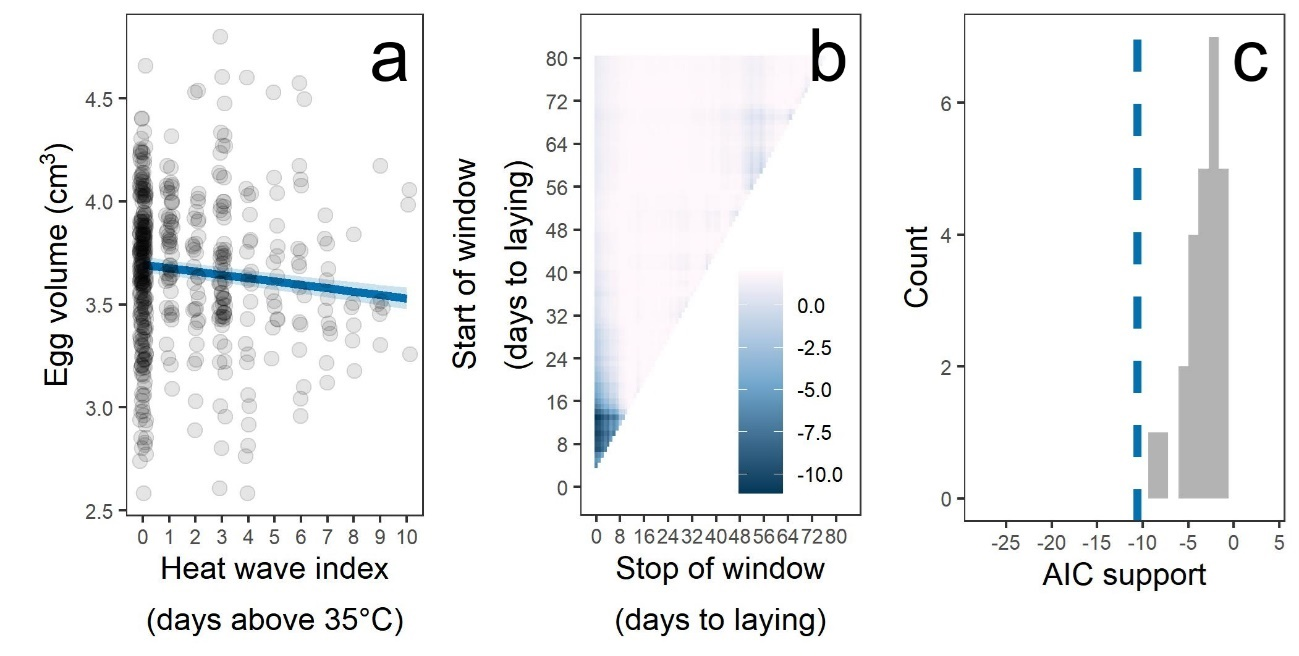

Supplement: S2 Fig — See Section A in S1 File above for methods and interpretation. (a) Effect of the best-supported “heat waves” index (i.e., that calculated for 0–13 days prior to egg laying) on egg volume when tested within the baseline model. Raw data points in black and regression line (± SE) in blue. (b) AIC support (i.e., the difference in AIC between a given sliding window model and the baseline model) for all possible sliding windows of >4 days in length within the 80 days before egg laying. The darker the color of the tiles, the stronger the support for a given window. (c) Histogram showing the AIC support for the best-supported heat wave index windows arising from 25 randomisations (i.e., the distribution of AIC support expected if no relationship exists between the heat waves index and egg volume). The blue dashed line illustrates the AIC support achieved using the best-supported window from the real data set. The data underlying this figure can be found at https://doi.org/10.5281/zenodo.8385995. (TIF) [file pbio.3002356.s003.tif]

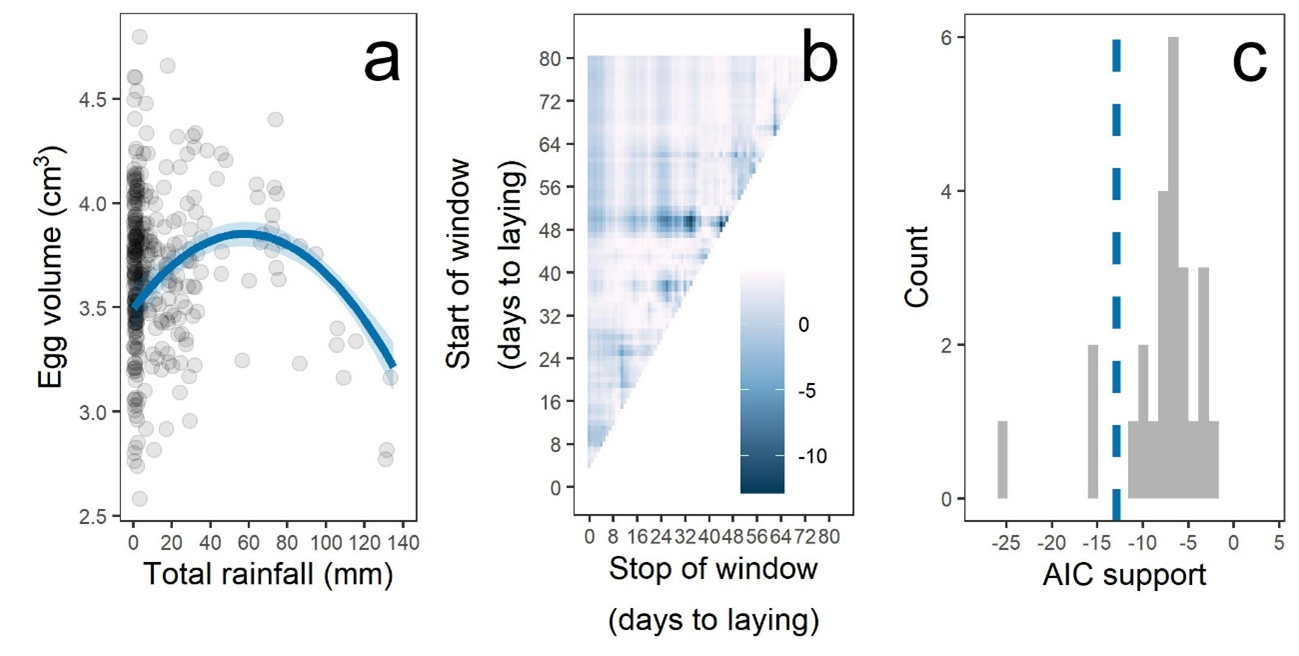

Supplement: S3 Fig — See Section A in S1 File above for methods and interpretation. (a) Effect of the best-supported total rainfall index (i.e., that calculated for 49–44 days prior to egg laying) on egg volume when tested within the baseline model. Raw data points in black and regression line (± SE) in blue. (b) AIC support (i.e., difference in AIC between a given sliding window model and the baseline model) for all possible sliding windows of >4 days in length within the 80 days before egg laying. The darker the color of the tiles, the stronger the support for a given window. (c) Histogram showing the AIC support for the best-supported rainfall index windows arising from the 25 randomizations (i.e., the distribution of AIC support expected if no relationship exists between the rainfall index and egg volume). The blue dashed line illustrates the AIC support achieved using the best-supported window from the real data set. The data underlying this figure can be found at https://doi.org/10.5281/zenodo.8385995. (TIF) [file pbio.3002356.s004.tif]

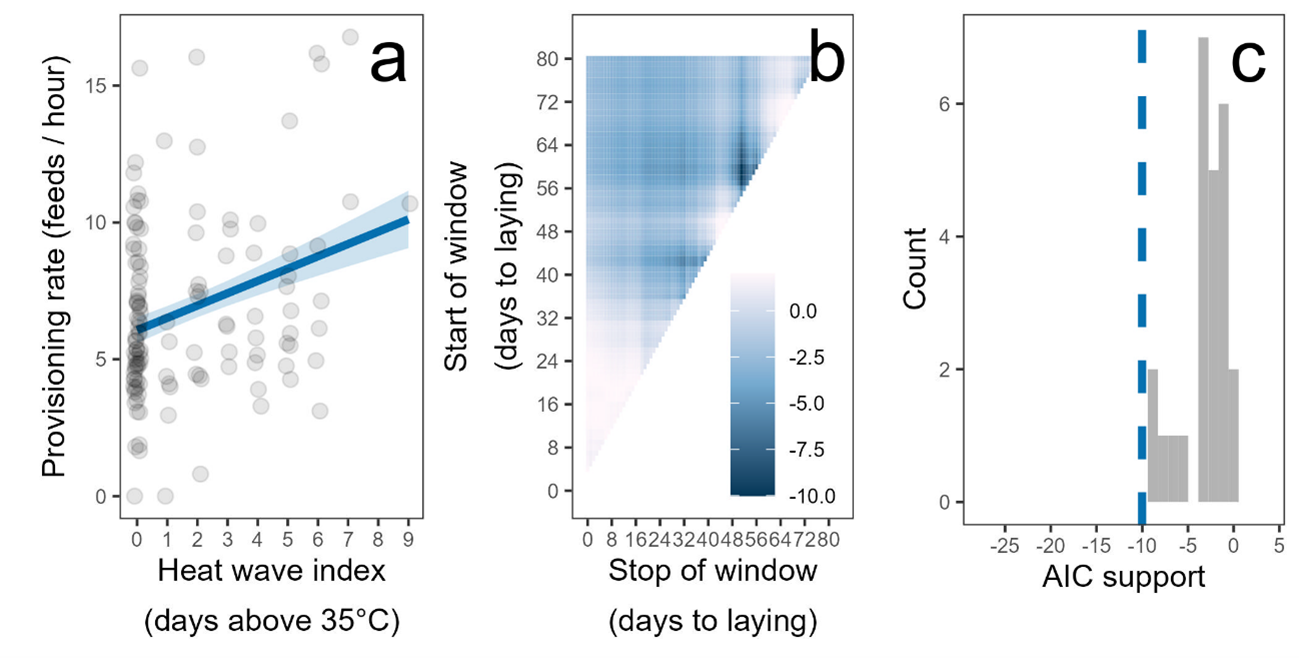

Supplement: S4 Fig — See Section A in S1 File above for methods and interpretation. (a) Effect of the best-supported “heat waves” index (i.e., that calculated for 59–51 days prior to egg laying) on maternal provisioning rate when tested within the baseline model. Raw data points in black and regression line (± SE) in blue. (b) AIC support (i.e., difference in AIC between a given sliding window model and the baseline model) for all possible sliding windows of >4 days in length within the 80 days before egg laying. The darker the color of the tiles, the stronger the support for a given window. (c) Histogram showing the AIC support for the best-supported heat waves index windows arising from 25 randomizations (i.e., the distribution of AIC support expected if no relationship exists between the heat waves index and maternal provisioning rate). The blue dashed line illustrates the AIC support achieved using the best-supported window from the real data set. The data underlying this figure can be found at https://doi.org/10.5281/zenodo.8385995. (TIF) [file pbio.3002356.s005.tif]

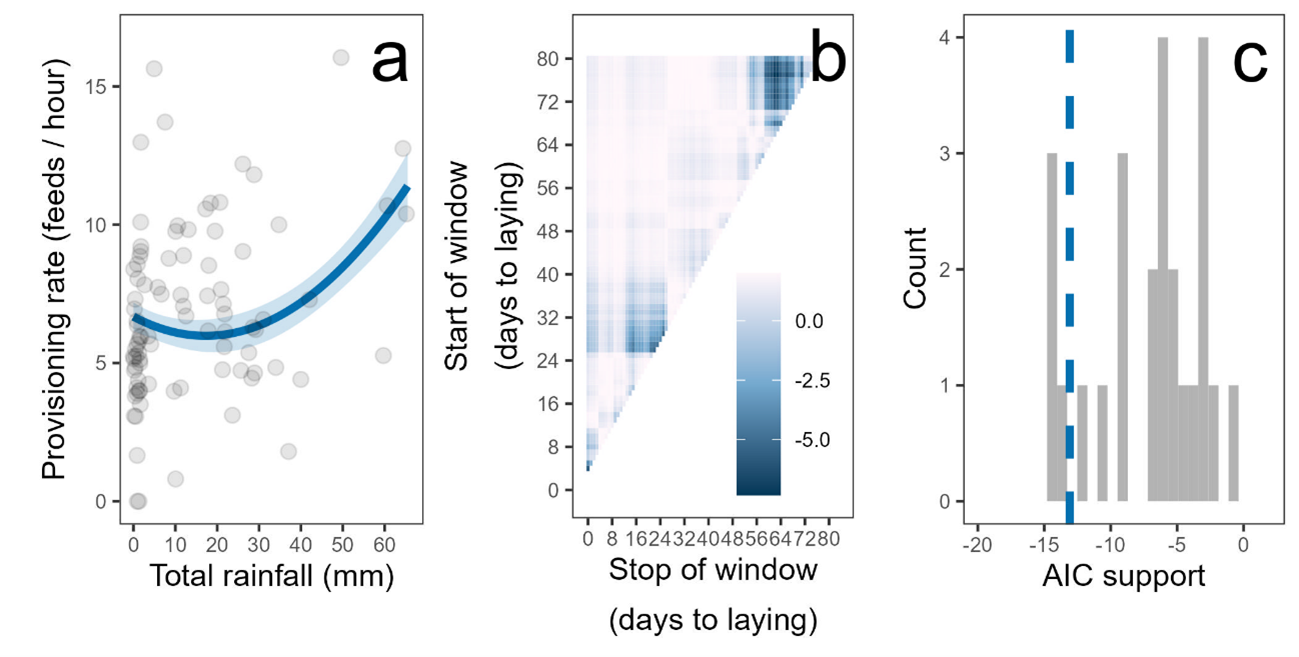

Supplement: S5 Fig — See Section A in S1 File above for methods and interpretation. (a) Effect of the best-supported total rainfall index (i.e., that calculated for 78–61 days prior to egg laying) on maternal provisioning rate when tested within the baseline model. Raw data points in black and regression line (± SE) in blue. (b) AIC support (i.e., difference in AIC between a given sliding window model and the baseline model) for all possible sliding windows of >4 days in length within the 80 days before egg laying. The darker the color of the tiles, the stronger the support for a given window. (c) Histogram showing the AIC support for the best-supported rainfall index windows from each of the 25 randomizations (i.e., the distribution of AIC support expected if no relationship exists between the rainfall index and maternal provisioning rate). The blue dashed line illustrates the AIC support achieved using the best-supported window from the real data set. The data underlying this figure can be found at https://doi.org/10.5281/zenodo.8385995. (TIF) [file pbio.3002356.s006.tif]

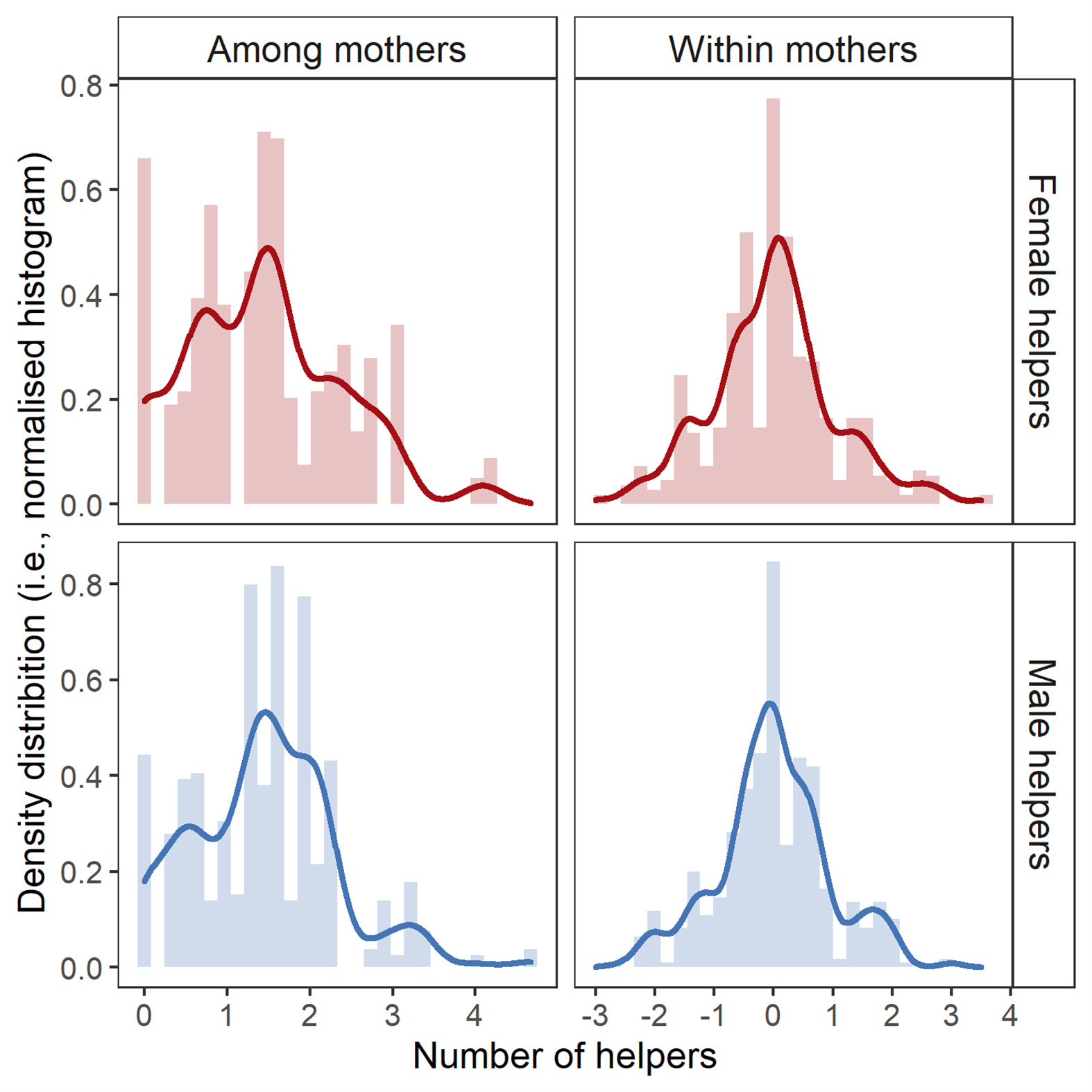

Supplement: S6 Fig — Analogous distributions for female and male indicate that the power to detect female and male effects was similar. The data underlying this figure can be found at https://doi.org/10.5281/zenodo.8385995. (TIF) [file pbio.3002356.s007.tif]

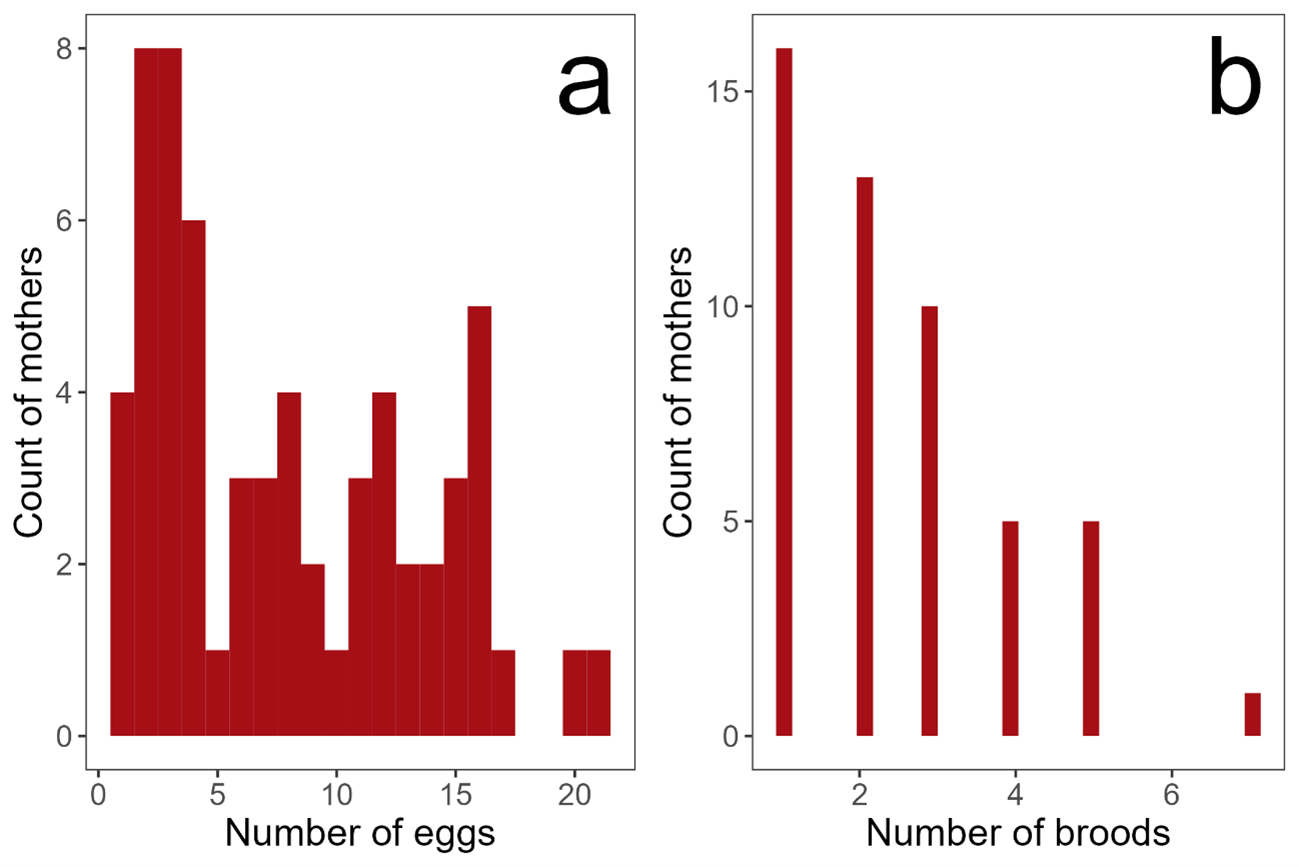

Supplement: S7 Fig — (a) Number of eggs per mother included in our egg volume analysis and (b) number of broods per female in our maternal provisioning analysis. The data underlying this figure can be found at https://doi.org/10.5281/zenodo.8385995. (TIF) [file pbio.3002356.s008.tif]
