# Supplementary material for: Mothers in a cooperatively breeding bird increase investment per offspring at the pre-natal stage when they will have more help with post-natal care
Source: PLoS Biol. 2023 Nov 9;21(11):e3002356. doi: 10.1371/journal.pbio.3002356 (PMC10635431; doi:10.1371/journal.pbio.3002356)
Supplement: S2 Table — Model estimates, standard errors (SE), and their 95% confidence intervals (CI (95%)) are provided along with results from likelihood-ratio tests (χ2df = 1 and associated p-values) assessing the statistical significance of each predictor within the full model (i.e., a model containing all of the terms in the table below). Random effect standard deviation: “mother ID” = 0.230 g. (DOCX) [file pbio.3002356.s010.docx]

**S2 Table.** Egg volume (cm^3^) effects on hatchling mass (g). Model estimates, standard errors (SE) and their 95% confidence intervals (CI (95%)) are provided along with results from likelihood-ratio tests (χ^2^_df = 1_ and associated p-values) assessing the statistical significance of each predictor within the full model (i.e., a model containing all of the terms in the table below). Random effect standard deviation: ‘mother ID’ = 0.230 g.

| **Predictors** | **Estimates** | **SE** | **CI (95%)** | **χ ^2^_1_** | **p-value** |
| --- | --- | --- | --- | --- | --- |
| Intercept | 0.861 | 0.465 | -0.057,1.778 |  |  |
| Egg volume (cm^3^) | 0.679 | 0.124 | 0.434,0.924 | 27.23 | < 0.001 |
