# Supplementary material for: Mothers in a cooperatively breeding bird increase investment per offspring at the pre-natal stage when they will have more help with post-natal care
Source: PLoS Biol. 2023 Nov 9;21(11):e3002356. doi: 10.1371/journal.pbio.3002356 (PMC10635431; doi:10.1371/journal.pbio.3002356)
Supplement: S3 Table — Note that partitioning within-mother (Δ) and among-mother (μ) components might produce bias in the among mother component [70]; however, this method produces robust estimation of the within-mother component, which the evidence of plasticity is based upon. Model estimates, standard errors (SE), and their 95% confidence intervals (CI (95%)) are provided along with results from likelihood-ratio tests (χ2df = 1 and associated p-values) assessing the statistical significance of each predictor within the full model (i.e., a model containing all of the terms in the table below). Random effect standard deviation: “mother ID” = 0.231 g. (DOCX) [file pbio.3002356.s011.docx]

**S3 Table.** Within (Δ) and among mother (μ) effects of egg volume (cm^3^) on hatchling mass (g). Note that partitioning within-mother (Δ) and among-mother (µ) components might produce bias in the among mother component [24]; however, this method produces robust estimation of the within-mother component, which the evidence of plasticity is based upon. Model estimates, standard errors (SE) and their 95% confidence intervals (CI (95%)) are provided along with results from likelihood-ratio tests (χ^2^_df = 1_ and associated p-values) assessing the statistical significance of each predictor within the full model (i.e., a model containing all of the terms in the table below). Random effect standard deviation: ‘mother ID’ = 0.231 g.

| **Predictors** | **Estimates** | **SE** | **CI (95%)** | **χ ^2^_1_** | **p-value** |
| --- | --- | --- | --- | --- | --- |
| Intercept | 1.125 | 0.556 | 0.029,2.222 | - | - |
| Δ Egg volume | 0.846 | 0.228 | 0.397,1.295 | 13.22 | <0.001 |
| μ Egg volume | 0.608 | 0.149 | 0.314,0.902 | 14.55 | <0.001 |
