# Supplementary material for: Mothers in a cooperatively breeding bird increase investment per offspring at the pre-natal stage when they will have more help with post-natal care
Source: PLoS Biol. 2023 Nov 9;21(11):e3002356. doi: 10.1371/journal.pbio.3002356 (PMC10635431; doi:10.1371/journal.pbio.3002356)
Supplement: S6 Table — Model estimates, standard errors (SE), and their 95% confidence intervals (CI (95%)) are provided along with results from likelihood-ratio tests (χ2df = 1 and associated p-values) assessing the statistical significance of each predictor within the full model (i.e., a model containing all of the terms in the table below). Random effect standard deviation: “season” = 0 clutches, “group ID” = 0 clutches, “mother ID” = 0 clutches. (DOCX) [file pbio.3002356.s014.docx]

**S6 Table.** Summary of results of a generalised linear mixed models with zero-truncated Poisson error explaining variation in clutch size (N = 344 clutches eggs laid by 66 mothers in 37 social groups). Model estimates, standard errors (SE) and their 95% confidence intervals (CI (95%)) are provided along with results from likelihood-ratio tests (χ^2^_df = 1_ and associated p-values) assessing the statistical significance of each predictor within the full model (i.e., a model containing all of the terms in the table below). Random effect standard deviation: ‘season’ = 0 clutches, ‘group ID’ = 0 clutches, ‘, ‘mother ID’ = 0 clutches.

| **Predictors** | **Estimates** | **SE** | **CI (95%)** | **χ ^2^_1_** | **p-value** |
| --- | --- | --- | --- | --- | --- |
| Intercept | 0.578 | 0.136 | 0.311, 0.846 |  |  |
| Clutch order | 0.032 | 0.046 | -0.057, 0.122 | 0.50 | 0.478 |
| Number of female helpers | -0.046 | 0.041 | -0.125, 0.034 | 1.29 | 0.256 |
| Number of male helpers | -0.043 | 0.047 | -0.134, 0.049 | 0.85 | 0.357 |
