# Supplementary material for: Mothers in a cooperatively breeding bird increase investment per offspring at the pre-natal stage when they will have more help with post-natal care
Source: PLoS Biol. 2023 Nov 9;21(11):e3002356. doi: 10.1371/journal.pbio.3002356 (PMC10635431; doi:10.1371/journal.pbio.3002356)
Supplement: S7 Table — Model estimates, standard errors (SE), and their 95% confidence intervals (CI (95%)) are provided along with results from likelihood-ratio tests (χ2df = 1 and associated p-values) assessing the statistical significance of each predictor within the full model (i.e., a model containing all of the terms in the table below). Random effect standard deviation: “season” = 0 clutches, “group ID” = 0 clutches, “mother ID” = 0 clutches. (DOCX) [file pbio.3002356.s015.docx]

**S7 Table.** Summary of results of a generalised linear mixed models with zero-truncated Poisson error explaining variation in clutch size (N = 344 clutches eggs laid by 66 mothers in 37 social groups) when population-level variation in female and male helper number were partitioned into their within-mother (Δ) and among-mother (µ) components. Model estimates, standard errors (SE) and their 95% confidence intervals (CI (95%)) are provided along with results from likelihood-ratio tests (χ^2^_df = 1_ and associated p-values) assessing the statistical significance of each predictor within the full model (i.e., a model containing all of the terms in the table below). Random effect standard deviation: ‘season’ = 0 clutches, ‘group ID’ = 0 clutches, ‘mother ID’ = 0 clutches.

| **Predictors** | **Estimates** | **SE** | **95% CI** | **χ ^2^_1_** | **p-value** |
| --- | --- | --- | --- | --- | --- |
| Intercept | 0.643 | 0.212 | 0.226, 1.059 |  |  |
| Clutch order | 0.031 | 0.046 | -0.060, 0.121 | 0.44 | 0.506 |
| Δ Number of female helpers | -0.047 | 0.051 | -0.146, 0.053 | 0.85 | 0.358 |
| Δ Number of male helpers | -0.049 | 0.060 | -0.166, 0.069 | 0.66 | 0.416 |
| µ Number of female helpers | -0.043 | 0.065 | -0.170, 0.085 | 0.43 | 0.510 |
| µ Number of male helpers | -0.033 | 0.073 | -0.176, 0.110 | 0.21 | 0.650 |
