# Supplementary material for: Mothers in a cooperatively breeding bird increase investment per offspring at the pre-natal stage when they will have more help with post-natal care
Source: PLoS Biol. 2023 Nov 9;21(11):e3002356. doi: 10.1371/journal.pbio.3002356 (PMC10635431; doi:10.1371/journal.pbio.3002356)
Supplement: S8 Table — This analysis revealed very week evidence for an effect of female or male helper number on clutch size. Models including female or male helper number received similar support from the data that the intercept-only model. Model coefficients (effect sizes ± standard errors) are shown along with number of model parameters (“k”), AIC and ΔAIC. (DOCX) [file pbio.3002356.s016.docx]

**S8 Table.** Model selection table for models explaining variation in clutch size (zero-truncated models). This analysis revealed very week evidence for an effect of female or male helper number on clutch size. Models including female or male helper number received similar support from the data that the intercept-only model. Model coefficients (effect sizes ± standard errors) are shown along with number of model parameters (‘k’), AIC and ΔAIC.

| **Intercept** | **Number of helping females** | **Number of male helpers** | **Clutch order** | **k** | **AIC** | **ΔAIC** |
| --- | --- | --- | --- | --- | --- | --- |
| 0.63 ± 0.06 | −0.06 ± 0.04 |  |  | 5 | 851.40 | 0.00 |
| 0.63 ± 0.07 |  | −0.07 ± 0.04 |  | 5 | 851.86 | 0.46 |
| 0.55 ± 0.05 |  |  |  | 4 | 851.95 | 0.55 |
| 0.66 ± 0.07 | −0.05 ± 0.04 | −0.05 ± 0.05 |  | 6 | 852.40 | 1.00 |
| 0.54 ± 0.13 | −0.06 ± 0.04 |  | 0.04 ± 0.05 | 6 | 852.74 | 1.34 |
| 0.45 ± 0.12 |  |  | 0.04 ± 0.05 | 5 | 853.08 | 1.68 |
| 0.54 ± 0.13 |  | −0.06 ± 0.04 | 0.04 ± 0.05 | 6 | 853.18 | 1.78 |
| 0.58 ± 0.14 | −0.05 ± 0.04 | −0.04 ± 0.05 | 0.03 ± 0.05 | 7 | 853.89 | 2.49 |
