# Supplementary material for: Mothers in a cooperatively breeding bird increase investment per offspring at the pre-natal stage when they will have more help with post-natal care
Source: PLoS Biol. 2023 Nov 9;21(11):e3002356. doi: 10.1371/journal.pbio.3002356 (PMC10635431; doi:10.1371/journal.pbio.3002356)
Supplement: S9 Table — Models including female or male helper number components received similar support from the data that the intercept-only model. Model coefficients (effect sizes ± standard errors) are shown along with number of model parameters (“k”), AIC and ΔAIC. “Clutch order” was mean centered and scaled by one standard deviation prior model fit to improve model convergence. (DOCX) [file pbio.3002356.s017.docx]

**S9 Table.** Model selection table for models explaining variation in clutch size (zero-truncated models) after partitioning variation in female and male helper number into their within-mother (Δ) and among-mother (µ) components. Models including female or male helper number components received similar support from the data that the intercept-only model. Model coefficients (effect sizes ± standard errors) are shown along with number of model parameters (‘k’), AIC and ΔAIC. ‘Clutch order’ was mean centered and scaled by one standard deviation prior model fit to improve model convergence.

| **Intercept** | **Δ Number of female helpers** | **μ Number of female helpers** | **Δ Number of male helpers** | **μ Number of male helpers** | **Clutch order** | **k** | **AIC** | **ΔAIC** |
| --- | --- | --- | --- | --- | --- | --- | --- | --- |
| 0.55 ± 0.05 | −0.07 ± 0.05 |  |  |  |  | 5 | 851.94 | 0.00 |
| 0.55 ± 0.05 |  |  |  |  |  | 4 | 851.95 | 0.01 |
| 0.55 ± 0.05 |  |  | −0.08 ± 0.05 |  |  | 5 | 852.07 | 0.13 |
| 0.45 ± 0.12 |  |  |  |  | 0.04 ± 0.05 | 5 | 853.08 | 1.14 |
| 0.55 ± 0.05 | −0.05 ± 0.05 |  | −0.05 ± 0.06 |  |  | 6 | 853.11 | 1.17 |
| 0.66 ± 0.14 | −0.07 ± 0.05 | −0.05 ± 0.06 |  |  |  | 6 | 853.32 | 1.38 |
| 0.47 ± 0.12 | −0.06 ± 0.05 |  |  |  | 0.04 ± 0.05 | 6 | 853.35 | 1.41 |
| 0.66 ± 0.14 |  | −0.05 ± 0.06 | −0.08 ± 0.05 |  |  | 6 | 853.49 | 1.55 |
| 0.47 ± 0.12 |  |  | −0.07 ± 0.05 |  | 0.03 ± 0.05 | 6 | 853.52 | 1.58 |
| 0.64 ± 0.15 |  | −0.04 ± 0.07 |  |  |  | 5 | 853.57 | 1.63 |
| 0.64 ± 0.16 | −0.07 ± 0.05 |  |  | −0.04 ± 0.07 |  | 6 | 853.59 | 1.65 |
| 0.65 ± 0.16 |  |  | −0.08 ± 0.05 | −0.04 ± 0.07 |  | 6 | 853.69 | 1.75 |
| 0.63 ± 0.16 |  |  |  | −0.04 ± 0.07 |  | 5 | 853.71 | 1.77 |
| 0.66 ± 0.14 | −0.05 ± 0.05 | −0.05 ± 0.06 | −0.05 ± 0.06 |  |  | 7 | 854.48 | 2.54 |
| 0.55 ± 0.18 |  | −0.05 ± 0.07 |  |  | 0.05 ± 0.05 | 6 | 854.59 | 2.65 |
| 0.48 ± 0.12 | −0.05 ± 0.05 |  | −0.05 ± 0.06 |  | 0.03 ± 0.05 | 7 | 854.70 | 2.76 |
| 0.58 ± 0.18 | −0.06 ± 0.05 | −0.05 ± 0.06 |  |  | 0.04 ± 0.05 | 7 | 854.71 | 2.77 |
| 0.64 ± 0.16 | −0.05 ± 0.05 |  | −0.05 ± 0.06 | −0.04 ± 0.07 |  | 7 | 854.73 | 2.79 |
| 0.53 ± 0.19 |  |  |  | −0.04 ± 0.07 | 0.05 ± 0.05 | 6 | 854.75 | 2.81 |
| 0.58 ± 0.18 |  | −0.05 ± 0.06 | −0.07 ± 0.05 |  | 0.03 ± 0.05 | 7 | 854.92 | 2.98 |
| 0.56 ± 0.19 | −0.06 ± 0.05 |  |  | −0.04 ± 0.07 | 0.04 ± 0.05 | 7 | 854.95 | 3.01 |
| 0.57 ± 0.19 |  |  | −0.07 ± 0.05 | −0.05 ± 0.07 | 0.04 ± 0.05 | 7 | 855.10 | 3.16 |
| 0.71 ± 0.19 | −0.07 ± 0.05 | −0.04 ± 0.06 |  | −0.03 ± 0.07 |  | 7 | 855.15 | 3.21 |
| 0.71 ± 0.19 |  | −0.04 ± 0.06 | −0.08 ± 0.05 | −0.03 ± 0.07 |  | 7 | 855.30 | 3.36 |
| 0.69 ± 0.20 |  | −0.04 ± 0.07 |  | −0.03 ± 0.07 |  | 6 | 855.43 | 3.49 |
| 0.59 ± 0.18 | −0.05 ± 0.05 | −0.05 ± 0.06 | −0.05 ± 0.06 |  | 0.03 ± 0.05 | 8 | 856.06 | 4.12 |
| 0.58 ± 0.19 | −0.05 ± 0.05 |  | −0.05 ± 0.06 | −0.05 ± 0.07 | 0.03 ± 0.05 | 8 | 856.29 | 4.35 |
| 0.71 ± 0.19 | −0.05 ± 0.05 | −0.04 ± 0.06 | −0.05 ± 0.06 | −0.03 ± 0.07 |  | 8 | 856.30 | 4.36 |
| 0.60 ± 0.22 |  | −0.04 ± 0.07 |  | −0.03 ± 0.07 | 0.05 ± 0.05 | 7 | 856.40 | 4.46 |
| 0.63 ± 0.21 | −0.06 ± 0.05 | −0.04 ± 0.06 |  | −0.03 ± 0.07 | 0.04 ± 0.05 | 8 | 856.52 | 4.58 |
| 0.63 ± 0.21 |  | −0.04 ± 0.06 | −0.07 ± 0.05 | −0.03 ± 0.07 | 0.04 ± 0.05 | 8 | 856.70 | 4.76 |
| 0.64 ± 0.21 | −0.05 ± 0.05 | −0.04 ± 0.06 | −0.05 ± 0.06 | −0.03 ± 0.07 | 0.03 ± 0.05 | 9 | 857.86 | 5.92 |
