# Supplementary material for: Mothers in a cooperatively breeding bird increase investment per offspring at the pre-natal stage when they will have more help with post-natal care
Source: PLoS Biol. 2023 Nov 9;21(11):e3002356. doi: 10.1371/journal.pbio.3002356 (PMC10635431; doi:10.1371/journal.pbio.3002356)
Supplement: S10 Table — This table presents all models within ΔAIC < 6 of the top model. Model coefficients (effect sizes ± standard errors [SE]) are shown along with number of model parameters (“k”), AIC and ΔAIC. “Heat waves” (days above 35°C) and “Brood size” were mean centered and scaled by one standard deviation prior model fit to improve model convergence. Similarly, “Rainfall” and “Rainfall2” were fitted as orthogonal vectors, and their estimates are not back transformed in this table (i.e., units do not refer to the real data scale). (DOCX) [file pbio.3002356.s018.docx]

**S10 Table.** Model selection table for models explaining variation in maternal provisioning rate (feeds / hour). This table presents all models within ΔAIC < 6 of the top model. Model coefficients (effect sizes ± standard errors [SE]) are shown along with number of model parameters (‘k’), AIC and ΔAIC. ‘Heat waves’ (days above 35˚C) and ‘Brood size’ were mean centered and scaled by one standard deviation prior model fit to improve model convergence. Similarly, ‘Rainfall’ and ‘Rainfall^2^’ were fitted as orthogonal vectors and their estimates are not back transformed in this table (i.e., units do not refer to the real data scale).

| **Intercept** | **Number of female helpers** | **Number of male helpers** | **Brood size** | **Rainfall** | **Rainfall^2^** | **Heat waves** | **Number of female helpers x Brood size** | **Number of male helpers x Brood size** | **k** | **AIC** | **ΔAIC** |
| --- | --- | --- | --- | --- | --- | --- | --- | --- | --- | --- | --- |
| 7.37 ± 0.47 | −0.46 ± 0.19 |  | 1.45 ± 0.24 | 4.60 ± 3.02 | 7.69 ± 2.75 | 0.71 ± 0.30 |  |  | 10 | 606.99 | 0.00 |
| 7.46 ± 0.54 | −0.46 ± 0.19 | −0.07 ± 0.23 | 1.44 ± 0.24 | 4.55 ± 3.02 | 7.67 ± 2.75 | 0.70 ± 0.30 |  |  | 11 | 608.89 | 1.90 |
| 7.37 ± 0.47 | −0.46 ± 0.20 |  | 1.43 ± 0.32 | 4.64 ± 3.09 | 7.68 ± 2.76 | 0.71 ± 0.30 | 0.01 ± 0.20 |  | 11 | 608.99 | 2.00 |
| 7.46 ± 0.51 | −0.44 ± 0.19 | −0.13 ± 0.23 | 1.81 ± 0.36 | 4.45 ± 2.96 | 7.85 ± 2.73 | 0.71 ± 0.29 |  | −0.30 ± 0.20 | 12 | 609.01 | 2.02 |
| 7.35 ± 0.52 | −0.47 ± 0.20 |  | 1.47 ± 0.25 | 7.59 ± 2.79 | 8.79 ± 2.77 |  |  |  | 9 | 610.26 | 3.27 |
| 6.82 ± 0.43 |  |  | 1.47 ± 0.25 | 4.66 ± 3.10 | 7.64 ± 2.81 | 0.71 ± 0.31 |  |  | 9 | 610.47 | 3.48 |
| 7.46 ± 0.51 | −0.43 ± 0.20 | −0.13 ± 0.23 | 1.75 ± 0.39 | 4.75 ± 3.03 | 7.78 ± 2.73 | 0.69 ± 0.29 | 0.10 ± 0.20 | −0.32 ± 0.21 | 13 | 610.79 | 3.80 |
| 7.46 ± 0.54 | −0.45 ± 0.20 | −0.07 ± 0.23 | 1.42 ± 0.33 | 4.59 ± 3.09 | 7.66 ± 2.75 | 0.70 ± 0.30 | 0.01 ± 0.20 |  | 12 | 610.89 | 3.90 |
| 7.52 ± 0.58 | −0.46 ± 0.20 | −0.14 ± 0.23 | 1.45 ± 0.25 | 7.38 ± 2.80 | 8.72 ± 2.77 |  |  |  | 10 | 611.92 | 4.93 |
| 7.01 ± 0.48 |  | −0.19 ± 0.23 | 1.86 ± 0.36 | 4.59 ± 3.02 | 7.82 ± 2.78 | 0.67 ± 0.30 |  | −0.32 ± 0.21 | 11 | 612.13 | 5.14 |
| 7.35 ± 0.52 | −0.46 ± 0.20 |  | 1.41 ± 0.33 | 7.73 ± 2.84 | 8.73 ± 2.79 |  | 0.05 ± 0.20 |  | 10 | 612.20 | 5.21 |
| 6.96 ± 0.51 |  | −0.11 ± 0.23 | 1.46 ± 0.25 | 4.62 ± 3.09 | 7.59 ± 2.81 | 0.70 ± 0.31 |  |  | 10 | 612.29 | 5.30 |
| 7.55 ± 0.56 | −0.44 ± 0.20 | −0.20 ± 0.24 | 1.78 ± 0.36 | 7.16 ± 2.76 | 8.91 ± 2.75 |  |  | −0.28 ± 0.21 | 11 | 612.30 | 5.31 |
| 7.39 ± 0.44 | −0.45 ± 0.20 |  | 1.30 ± 0.24 | 4.82 ± 3.06 |  | 0.82 ± 0.30 |  |  | 9 | 612.35 | 5.36 |
| 7.43 ± 0.47 | −0.46 ± 0.20 |  | 1.33 ± 0.24 |  |  | 1.02 ± 0.28 |  |  | 8 | 612.67 | 5.68 |
