# Supplementary material for: Mothers in a cooperatively breeding bird increase investment per offspring at the pre-natal stage when they will have more help with post-natal care
Source: PLoS Biol. 2023 Nov 9;21(11):e3002356. doi: 10.1371/journal.pbio.3002356 (PMC10635431; doi:10.1371/journal.pbio.3002356)
Supplement: S11 Table — Model coefficients (effect sizes ± standard errors) are shown along with number of model parameters (“k”), AIC and ΔAIC. “Heat waves” (days above 35°C) and “Brood size” were mean centered and scaled by one standard deviation prior model fit to improve model convergence. Similarly, “Rainfall” and “Rainfall2” were fitted as orthogonal vectors, and their estimates are not back transformed in this table (i.e., units do not refer to the real data scale). (DOCX) [file pbio.3002356.s019.docx]

**S11 Table.** Model selection table for models explaining variation in maternal provisioning rate (feeds / hour), when population-level variation in female and male helper number were partitioned into their within-mother (Δ) and among-mother (µ) components prior to model selection. Model coefficients (effect sizes ± standard errors) are shown along with number of model parameters (‘k’), AIC and ΔAIC. ‘Heat waves’ (days above 35˚C) and ‘Brood size’ were mean centered and scaled by one standard deviation prior model fit to improve model convergence. Similarly, ‘Rainfall’ and ‘Rainfall^2^’ were fitted as orthogonal vectors and their estimates are not back transformed in this table (i.e., units do not refer to the real data scale).

| **Intercept** | **Δ Number of female helpers** | **μ Number of female helpers** | **Δ Number of male helpers** | **μ Number of male helpers** | **Brood size** | **Rainfall** | **Rainfall^2^** | **Heat waves** | **k** | **AIC** | **ΔAIC** |
| --- | --- | --- | --- | --- | --- | --- | --- | --- | --- | --- | --- |
| 6.85 ± 0.41 | −0.53 ± 0.26 |  |  |  | 1.48 ± 0.24 | 4.62 ± 3.04 | 7.74 ± 2.76 | 0.73 ± 0.30 | 10 | 608.40 | 0.00 |
| 7.27 ± 0.52 | −0.54 ± 0.26 | −0.36 ± 0.28 |  |  | 1.45 ± 0.24 | 4.59 ± 3.02 | 7.72 ± 2.75 | 0.72 ± 0.30 | 11 | 608.78 | 0.37 |
| 7.24 ± 0.61 | −0.56 ± 0.26 |  |  | −0.32 ± 0.39 | 1.46 ± 0.24 | 4.61 ± 3.02 | 7.78 ± 2.76 | 0.72 ± 0.30 | 11 | 609.77 | 1.36 |
| 6.85 ± 0.41 | −0.55 ± 0.27 |  | 0.06 ± 0.30 |  | 1.48 ± 0.24 | 4.66 ± 3.04 | 7.76 ± 2.77 | 0.74 ± 0.30 | 11 | 610.36 | 1.96 |
| 6.82 ± 0.43 |  |  |  |  | 1.47 ± 0.25 | 4.66 ± 3.10 | 7.64 ± 2.81 | 0.71 ± 0.31 | 9 | 610.47 | 2.07 |
| 7.45 ± 0.64 | −0.56 ± 0.26 | −0.33 ± 0.30 |  | −0.19 ± 0.40 | 1.45 ± 0.24 | 4.59 ± 3.01 | 7.73 ± 2.74 | 0.71 ± 0.30 | 12 | 610.56 | 2.16 |
| 7.27 ± 0.53 | −0.54 ± 0.27 | −0.37 ± 0.29 | −0.01 ± 0.30 |  | 1.45 ± 0.24 | 4.58 ± 3.02 | 7.71 ± 2.75 | 0.72 ± 0.30 | 12 | 610.77 | 2.37 |
| 7.22 ± 0.53 |  | −0.35 ± 0.29 |  |  | 1.45 ± 0.25 | 4.66 ± 3.07 | 7.60 ± 2.79 | 0.70 ± 0.31 | 10 | 611.01 | 2.61 |
| 7.23 ± 0.61 | −0.58 ± 0.27 |  | 0.08 ± 0.30 | −0.32 ± 0.39 | 1.46 ± 0.24 | 4.67 ± 3.03 | 7.80 ± 2.76 | 0.73 ± 0.30 | 12 | 611.70 | 3.30 |
| 6.83 ± 0.46 | −0.51 ± 0.27 |  |  |  | 1.49 ± 0.25 | 7.67 ± 2.80 | 8.83 ± 2.79 |  | 9 | 611.98 | 3.58 |
| 7.27 ± 0.57 | −0.53 ± 0.27 | −0.40 ± 0.29 |  |  | 1.47 ± 0.25 | 7.61 ± 2.79 | 8.82 ± 2.78 |  | 10 | 612.15 | 3.74 |
| 7.05 ± 0.62 |  |  |  | −0.20 ± 0.40 | 1.46 ± 0.25 | 4.69 ± 3.09 | 7.64 ± 2.81 | 0.70 ± 0.31 | 10 | 612.24 | 3.84 |
| 6.83 ± 0.42 |  |  | −0.06 ± 0.30 |  | 1.47 ± 0.25 | 4.63 ± 3.10 | 7.61 ± 2.81 | 0.71 ± 0.31 | 10 | 612.44 | 4.03 |
| 7.44 ± 0.64 | −0.56 ± 0.27 | −0.33 ± 0.30 | 0.01 ± 0.31 | −0.19 ± 0.41 | 1.45 ± 0.24 | 4.60 ± 3.02 | 7.74 ± 2.75 | 0.71 ± 0.30 | 13 | 612.56 | 4.16 |
| 7.26 ± 0.54 |  | −0.38 ± 0.29 | −0.14 ± 0.30 |  | 1.44 ± 0.25 | 4.58 ± 3.08 | 7.54 ± 2.79 | 0.68 ± 0.31 | 11 | 612.81 | 4.41 |
| 7.28 ± 0.65 |  | −0.34 ± 0.30 |  | −0.06 ± 0.41 | 1.44 ± 0.25 | 4.67 ± 3.07 | 7.60 ± 2.79 | 0.70 ± 0.31 | 11 | 612.99 | 4.58 |
| 7.29 ± 0.66 | −0.55 ± 0.27 |  |  | −0.38 ± 0.41 | 1.47 ± 0.25 | 7.59 ± 2.79 | 8.87 ± 2.78 |  | 10 | 613.13 | 4.72 |
| 6.81 ± 0.46 |  |  |  |  | 1.48 ± 0.25 | 7.68 ± 2.84 | 8.66 ± 2.83 |  | 8 | 613.63 | 5.22 |
| 6.87 ± 0.38 | −0.51 ± 0.27 |  |  |  | 1.33 ± 0.25 | 4.83 ± 3.09 |  | 0.84 ± 0.30 | 9 | 613.85 | 5.44 |
| 7.49 ± 0.69 | −0.55 ± 0.27 | −0.35 ± 0.30 |  | −0.23 ± 0.42 | 1.46 ± 0.25 | 7.57 ± 2.78 | 8.83 ± 2.77 |  | 11 | 613.86 | 5.45 |
| 6.84 ± 0.46 | −0.51 ± 0.27 |  | −0.03 ± 0.30 |  | 1.49 ± 0.25 | 7.63 ± 2.82 | 8.81 ± 2.79 |  | 10 | 613.97 | 5.56 |
| 7.23 ± 0.57 |  | −0.38 ± 0.30 |  |  | 1.46 ± 0.25 | 7.61 ± 2.83 | 8.62 ± 2.82 |  | 9 | 614.01 | 5.61 |
| 7.31 ± 0.58 | −0.51 ± 0.27 | −0.42 ± 0.30 | −0.11 ± 0.31 |  | 1.46 ± 0.25 | 7.47 ± 2.81 | 8.76 ± 2.78 |  | 11 | 614.03 | 5.62 |
| 6.91 ± 0.42 | −0.52 ± 0.27 |  |  |  | 1.36 ± 0.25 |  |  | 1.04 ± 0.28 | 8 | 614.11 | 5.70 |
| 7.06 ± 0.62 |  |  | −0.06 ± 0.30 | −0.20 ± 0.40 | 1.45 ± 0.25 | 4.66 ± 3.09 | 7.62 ± 2.81 | 0.69 ± 0.31 | 11 | 614.21 | 5.80 |
| 7.31 ± 0.50 | −0.52 ± 0.27 | −0.38 ± 0.29 |  |  | 1.30 ± 0.24 | 4.80 ± 3.06 |  | 0.83 ± 0.30 | 10 | 614.23 | 5.82 |
