# Supplementary material for: Mothers in a cooperatively breeding bird increase investment per offspring at the pre-natal stage when they will have more help with post-natal care
Source: PLoS Biol. 2023 Nov 9;21(11):e3002356. doi: 10.1371/journal.pbio.3002356 (PMC10635431; doi:10.1371/journal.pbio.3002356)
Supplement: S12 Table — Model estimates, standard errors (SE), and their 95% confidence intervals (CI (95%)) are provided along with results from likelihood-ratio tests (χ2df = 1 and associated p-values) assessing the statistical significance of each predictor within the full model. “Rainfall,” “Heat waves” (days above 35°C), “Clutch size,” and “Egg position” were mean centered and scaled by one standard deviation prior model fit to improve model convergence. (DOCX) [file pbio.3002356.s020.docx]

**S12 Table.** Summary of results of a linear mixed model explaining variation in egg volume (cm^3^), including every main effect of interest, but including data for rainfall values below its peak (N = 466 eggs). Model estimates, standard errors (SE) and their 95% confidence intervals (CI (95%)) are provided along with results from likelihood-ratio tests (χ^2^_df = 1_ and associated p-values) assessing the statistical significance of each predictor within the full model. ‘Rainfall’, ‘Heat waves’ (days above 35˚C), ‘Clutch size’ and ‘Egg position’ were mean centered and scaled by one standard deviation prior model fit to improve model convergence.

| **Predictors** | **Estimates** | **SE** | **95% CI** | **χ ^2^_1_** | **p-value** |
| --- | --- | --- | --- | --- | --- |
| Intercept | 3.626 | 0.040 | 3.547, 3.704 |  |  |
| Rainfall | -0.001 | 0.010 | -0.020, 0.018 | 0.01 | 0.918 |
| Heat waves | -0.040 | 0.010 | -0.059, -0.021 | 16.03 | < 0.001 |
| Number of female helpers | 0.019 | 0.009 | 0.002, 0.036 | 4.94 | 0.026 |
| Number of male helpers | 0.009 | 0.010 | -0.009, 0.028 | 0.96 | 0.327 |
| Clutch size | 0.007 | 0.011 | -0.016, 0.029 | 0.36 | 0.551 |
| Egg position | -0.042 | 0.009 | -0.060, -0.025 | 21.43 | < 0.001 |
