# Supplementary material for: Mothers in a cooperatively breeding bird increase investment per offspring at the pre-natal stage when they will have more help with post-natal care
Source: PLoS Biol. 2023 Nov 9;21(11):e3002356. doi: 10.1371/journal.pbio.3002356 (PMC10635431; doi:10.1371/journal.pbio.3002356)
Supplement: S13 Table — Model estimates, standard errors (SE), and their 95% confidence intervals (CI (95%)) are provided along with results from likelihood-ratio tests (χ2df = 1 and associated p-values) assessing the statistical significance of each predictor within the full model. “Rainfall,” “Heat waves” (days above 35°C), “Clutch size,” and “Egg position” were mean centered and scaled by one standard deviation prior model fit to improve model convergence. (DOCX) [file pbio.3002356.s021.docx]

**S13 Table.** Summary of results of a linear mixed model explaining variation in egg volume (cm^3^), including every main effect of interest after population-level variation in female and male helper number were partitioned into their within-mother (Δ) and among-mother (µ) components, including data for rainfall values below its peak (N = 466 eggs). Model estimates, standard errors (SE) and their 95% confidence intervals (CI (95%)) are provided along with results from likelihood-ratio tests (χ^2^_df = 1_ and associated p-values) assessing the statistical significance of each predictor within the full model. ‘Rainfall’, ‘Heat waves’ (days above 35˚C), ‘Clutch size’ and ‘Egg position’ were mean centered and scaled by one standard deviation prior model fit to improve model convergence.

| **Predictors** | **Estimates** | **SE** | **95% CI** | **χ ^2^_1_** | **p-value** |
| --- | --- | --- | --- | --- | --- |
| Intercept | 3.632 | 0.052 | 3.529, 3.734 |  |  |
| Rainfall | -0.001 | 0.010 | -0.020, 0.018 | 0.01 | 0.926 |
| Heat waves | -0.040 | 0.010 | -0.059, -0.021 | 16.02 | < 0.001 |
| Δ Number of female helpers | 0.019 | 0.009 | 0.002, 0.037 | 4.47 | 0.035 |
| µ Number of female helpers | 0.020 | 0.028 | -0.034, 0.074 | 0.52 | 0.472 |
| Δ Number of male helpers | 0.010 | 0.010 | -0.010, 0.030 | 0.99 | 0.319 |
| µ Number of male helpers | 0.004 | 0.029 | -0.052, 0.061 | 0.02 | 0.886 |
| Egg position | -0.042 | 0.009 | -0.060, -0.025 | 21.43 | < 0.001 |
| Clutch size | 0.007 | 0.012 | -0.016, 0.029 | 0.34 | 0.558 |
