# Supplementary material for: Mothers in a cooperatively breeding bird increase investment per offspring at the pre-natal stage when they will have more help with post-natal care
Source: PLoS Biol. 2023 Nov 9;21(11):e3002356. doi: 10.1371/journal.pbio.3002356 (PMC10635431; doi:10.1371/journal.pbio.3002356)
Supplement: S15 Table — Model estimates, standard errors (SE), and their 95% confidence intervals (CI (95%)) are provided along with results from likelihood-ratio tests (χ2df = 1 and associated p-values) assessing the statistical significance of each predictor within the full model. “Heat waves” (days above 35°C), “Clutch size,” and “Egg position” were mean centered and scaled by one standard deviation prior model fit to improve model convergence. (DOCX) [file pbio.3002356.s023.docx]

**S15 Table.** Summary of results of a linear mixed model explaining variation in egg volume (cm^3^), without the inclusion of rainfall and after population-level variation in female and male helper number were partitioned into their within-mother (Δ) and among-mother (µ) components (N = 490 eggs). Model estimates, standard errors (SE) and their 95% confidence intervals (CI (95%)) are provided along with results from likelihood-ratio tests (χ^2^_df = 1_ and associated p-values) assessing the statistical significance of each predictor within the full model. ‘Heat waves’ (days above 35˚C), ‘Clutch size’ and ‘Egg position’ were mean centered and scaled by one standard deviation prior model fit to improve model convergence.

| **Predictors** | **Estimates** | **SE** | **95% CI** | **χ ^2^_1_** | **p-value** |
| --- | --- | --- | --- | --- | --- |
| Intercept | 3.637 | 0.052 | 3.535, 3.739 |  |  |
| Heat waves | -0.038 | 0.010 | -0.057, -0.018 | 13.14 | < 0.001 |
| Δ Number of female helpers | 0.017 | 0.009 | -0.001, 0.036 | 3.38 | 0.066 |
| µ Number of female helpers | 0.011 | 0.028 | -0.044, 0.066 | 0.14 | 0.705 |
| Δ Number of male helpers | 0.010 | 0.011 | -0.011, 0.031 | 0.86 | 0.354 |
| µ Number of male helpers | 0.009 | 0.029 | -0.048, 0.066 | 0.09 | 0.759 |
| Egg position | -0.044 | 0.009 | -0.061, -0.026 | 23.23 | < 0.001 |
| Clutch size | 0.003 | 0.012 | -0.020, 0.025 | 0.06 | 0.811 |
