# Supplementary material for: Mothers in a cooperatively breeding bird increase investment per offspring at the pre-natal stage when they will have more help with post-natal care
Source: PLoS Biol. 2023 Nov 9;21(11):e3002356. doi: 10.1371/journal.pbio.3002356 (PMC10635431; doi:10.1371/journal.pbio.3002356)
Supplement: S16 Table — Model estimates, standard errors (SE), and their 95% confidence intervals (CI (95%)) are provided along with results from likelihood-ratio tests (χ2df = 1 and associated p-values) assessing the statistical significance of each predictor within the full model. “Heat waves” (days above 35°C) and “Brood size” were mean centered and scaled by one standard deviation prior model fit to improve model convergence. (DOCX) [file pbio.3002356.s024.docx]

**S16 Table.** Summary of results of a linear mixed model explaining variation in maternal provisioning rate (feeds / hour), without the inclusion of rainfall and at the population-level. Model estimates, standard errors (SE) and their 95% confidence intervals (CI (95%)) are provided along with results from likelihood-ratio tests (χ^2^_df = 1_ and associated p-values) assessing the statistical significance of each predictor within the full model. ‘Heat waves’ (days above 35˚C) and ‘Brood size’ was mean centered and scaled by one standard deviation prior model fit to improve model convergence.

| **Predictors** | **Estimates** | **SE** | **95% CI** | **χ ^2^_1_** | **p-value** |
| --- | --- | --- | --- | --- | --- |
| Intercept | 7.548 | 0.544 | 6.482, 8.615 |  |  |
| Heat waves | 0.996 | 0.281 | 0.446, 1.545 | 11.85 | 0.001 |
| Number of female helpers | -0.454 | 0.202 | -0.849, -0.058 | 4.93 | 0.026 |
| Number of male helpers | -0.103 | 0.237 | -0.566, 0.361 | 0.19 | 0.665 |
| Brood size | 1.321 | 0.246 | 0.839, 1.803 | 25.12 | < 0.001 |
