# Supplementary material for: Mothers in a cooperatively breeding bird increase investment per offspring at the pre-natal stage when they will have more help with post-natal care
Source: PLoS Biol. 2023 Nov 9;21(11):e3002356. doi: 10.1371/journal.pbio.3002356 (PMC10635431; doi:10.1371/journal.pbio.3002356)
Supplement: S17 Table — Model estimates, standard errors (SE), and their 95% confidence intervals (CI (95%)) are provided along with results from likelihood-ratio tests (χ2df = 1 and associated p-values) assessing the statistical significance of each predictor within the full model. “Heat waves” (days above 35°C) and “Brood size” were mean centered and scaled by one standard deviation prior model fit to improve model convergence. (DOCX) [file pbio.3002356.s025.docx]

**S17 Table.** Summary of results of a linear mixed model explaining variation in maternal provisioning rate (feeds / hour), without the inclusion of rainfall and after population-level variation in female and male helper number were partitioned into their within-mother (Δ) and among-mother (µ) components. Model estimates, standard errors (SE) and their 95% confidence intervals (CI (95%)) are provided along with results from likelihood-ratio tests (χ^2^_df = 1_ and associated p-values) assessing the statistical significance of each predictor within the full model. ‘Heat waves’ (days above 35˚C) and ‘Brood size’ was mean centered and scaled by one standard deviation prior model fit to improve model convergence.

| **Predictors** | **Estimates** | **SE** | **95% CI** | **χ ^2^_1_** | **p-value** |
| --- | --- | --- | --- | --- | --- |
| Intercept | 7.498 | 0.658 | 6.208, 8.788 |  |  |
| Heat waves | 1.006 | 0.282 | 0.453, 1.559 | 11.97 | 0.001 |
| Δ Number of female helpers | -0.530 | 0.279 | -1.078, 0.017 | 3.55 | 0.060 |
| µ Number of female helpers | -0.357 | 0.316 | -0.976, 0.262 | 1.27 | 0.260 |
| Δ Number of male helpers | -0.065 | 0.318 | -0.689, 0.558 | 0.04 | 0.837 |
| µ Number of male helpers | -0.153 | 0.424 | -0.984, 0.678 | 0.13 | 0.719 |
| Brood size | 1.326 | 0.246 | 0.843, 1.809 | 25.26 | <0.001 |
