# Supplementary material for: Mothers in a cooperatively breeding bird increase investment per offspring at the pre-natal stage when they will have more help with post-natal care
Source: PLoS Biol. 2023 Nov 9;21(11):e3002356. doi: 10.1371/journal.pbio.3002356 (PMC10635431; doi:10.1371/journal.pbio.3002356)
Supplement: S18 Table — Model estimates, standard errors (SE), and their 95% confidence intervals (CI (95%)) are provided along with results from likelihood-ratio tests (χ2df = 1 and associated p-values) assessing the statistical significance of each predictor within the full model. Random effect standard deviation: “mother ID” = 0 clutches, “group ID” = 0 clutches; breeding season = 0.470 clutches (χ21 = 64.65, p < 0.001). (DOCX) [file pbio.3002356.s026.docx]

**S18 Table.** Summary of results of a generalized linear mixed model (Poisson error structure) explaining variation in the number of clutches laid per year. Model estimates, standard errors (SE) and their 95% confidence intervals (CI (95%)) are provided along with results from likelihood-ratio tests (χ^2^_df = 1_ and associated p-values) assessing the statistical significance of each predictor within the full model. Random effect standard deviation: ‘mother ID’ = 0 clutches, ‘group ID’ = 0 clutches; breeding season = 0.470 clutches (χ ^2^_1_= 64.65, p < 0.001).

| **Predictors** | **Estimates** | **SE** | **95% CI** | **χ ^2^_1_** | **p-value** |
| --- | --- | --- | --- | --- | --- |
| Intercept | 0.234 | 0.437 | -0.623, 1.091 |  | 0.592 |
| Number of female helpers | 0.054 | 0.041 | -0.026, 0.134 | 1.74 | 0.187 |
| Number of male helpers | 0.087 | 0.048 | -0.007, 0.181 | 3.23 | 0.072 |
| Rainfall | 0.001 | 0.001 | -0.001, 0.003 | 0.72 | 0.397 |
