# Supplementary material for: Mothers in a cooperatively breeding bird increase investment per offspring at the pre-natal stage when they will have more help with post-natal care
Source: PLoS Biol. 2023 Nov 9;21(11):e3002356. doi: 10.1371/journal.pbio.3002356 (PMC10635431; doi:10.1371/journal.pbio.3002356)
Supplement: S19 Table — Model estimates, standard errors (SE), and their 95% confidence intervals (CI (95%)) are provided along with results from likelihood-ratio tests (χ2df = 1 and associated p-values) assessing the statistical significance of each predictor within the full model. Random effect standard deviation: “mother ID” = 0 clutches, “group ID” = 0 clutches; breeding season = 0.45 clutches (χ21 = 55.81, p < 0.001). (DOCX) [file pbio.3002356.s027.docx]

**S19 Table.** Summary of results of a generalized linear mixed model (Poisson error structure) explaining variation in the number of clutches laid per year after population-level variation in female and male helper number were partitioned into their within-mother (Δ) and among-mother (µ) components. Model estimates, standard errors (SE) and their 95% confidence intervals (CI (95%)) are provided along with results from likelihood-ratio tests (χ^2^_df = 1_ and associated p-values) assessing the statistical significance of each predictor within the full model. Random effect standard deviation: ‘mother ID’ = 0 clutches, ‘group ID’ = 0 clutches; breeding season = 0.45 clutches (χ ^2^_1_= 55.81, p < 0.001).

| **Predictors** | **Estimates** | **SE** | **95% CI** | **χ ^2^_1_** | **p-value** |
| --- | --- | --- | --- | --- | --- |
| Intercept | -0.126 | 0.465 | -1.037, 0.786 |  |  |
| Δ Number of female helpers | 0.010 | 0.054 | -0.095, 0.115 | 0.03 | 0.855 |
| µ Number of female helpers | 0.120 | 0.075 | -0.027, 0.266 | 2.53 | 0.112 |
| Δ Number of male helpers | 0.054 | 0.061 | -0.066, 0.175 | 0.77 | 0.379 |
| µ Number of male helpers | 0.125 | 0.088 | -0.046, 0.297 | 2.03 | 0.155 |
| Rainfall | 0.001 | 0.001 | -0.001, 0.003 | 0.77 | 0.380 |
