# Supplementary material for: Mothers in a cooperatively breeding bird increase investment per offspring at the pre-natal stage when they will have more help with post-natal care
Source: PLoS Biol. 2023 Nov 9;21(11):e3002356. doi: 10.1371/journal.pbio.3002356 (PMC10635431; doi:10.1371/journal.pbio.3002356)
Supplement: S20 Table — Model estimates, standard errors (SE), and their 95% confidence intervals (CI (95%)) are provided along with results from likelihood-ratio tests (χ2df = 1 and associated p-values) assessing the statistical significance of each predictor within the full model. (DOCX) [file pbio.3002356.s028.docx]

**S20 Table.** Effect of the number of female and male helper numbers on a previous breeding attempt on egg volume (cm^3^). Model estimates, standard errors (SE) and their 95% confidence intervals (CI (95%)) are provided along with results from likelihood-ratio tests (χ^2^_df = 1_ and associated p-values) assessing the statistical significance of each predictor within the full model.

| **Predictors** | **Estimates** | **SE** | **95% CI** | **χ ^2^_1_** | **p-value** |
| --- | --- | --- | --- | --- | --- |
| Intercept | 45.099 | 1.790 | 41.591, 48.607 |  |  |
| Number of female helpers in previous breeding attempt | -0.232 | 0.350 | -0.918, 0.455 | 0.36 | 0.551 |
| Number of male helpers in previous breeding attempt | 0.108 | 0.404 | -0.683, 0.899 | 0.03 | 0.862 |
| Clutch order | -0.537 | 0.511 | -1.538, 0.463 | 0.96 | 0.328 |
