# Supplementary material for: Mothers in a cooperatively breeding bird increase investment per offspring at the pre-natal stage when they will have more help with post-natal care
Source: PLoS Biol. 2023 Nov 9;21(11):e3002356. doi: 10.1371/journal.pbio.3002356 (PMC10635431; doi:10.1371/journal.pbio.3002356)
Supplement: S21 Table — Model estimates, standard errors (SE), and their 95% confidence intervals (CI (95%)) are provided along with results from likelihood-ratio tests (χ2df = 1 and associated p-values) assessing the statistical significance of each predictor within the full model. (DOCX) [file pbio.3002356.s029.docx]

**S21 Table.** Effect of ttime since the last breeding attempt on egg volume (cm^3^). Model estimates, standard errors (SE) and their 95% confidence intervals (CI (95%)) are provided along with results from likelihood-ratio tests (χ^2^_df = 1_ and associated p-values) assessing the statistical significance of each predictor within the full model.

| **Predictors** | **Estimates** | **SE** | **95% CI** | **χ ^2^_1_** | **p-value** |
| --- | --- | --- | --- | --- | --- |
| Intercept | 3.751 | 0.074 | 3.606, 3.895 |  |  |
| Rainfall | -0.376 | 0.246 | -0.857, 0.106 | 2.09 | 0.149 |
| Rainfall^2^ | -0.342 | 0.198 | -0.730, 0.047 | 2.62 | 0.106 |
| Heat waves | -0.023 | 0.010 | -0.043, -0.002 | 4.02 | 0.045 |
| Time since previous breeding attempt | 0.000 | 0.000 | 0.000, 0.001 | 0.37 | 0.544 |
| Number of female helpers | 0.003 | 0.015 | -0.028, 0.033 | 0.02 | 0.879 |
| Number of male helpers | -0.025 | 0.016 | -0.057, 0.006 | 2.24 | 0.134 |
| Clutch size | -0.038 | 0.024 | -0.086, 0.010 | 2.34 | 0.126 |
| Egg position | -0.051 | 0.016 | -0.082, -0.020 | 9.95 | 0.002 |
