# Supplementary material for: Mothers in a cooperatively breeding bird increase investment per offspring at the pre-natal stage when they will have more help with post-natal care
Source: PLoS Biol. 2023 Nov 9;21(11):e3002356. doi: 10.1371/journal.pbio.3002356 (PMC10635431; doi:10.1371/journal.pbio.3002356)
Supplement: S22 Table — This model was fitted in R using the MCMCglmm package (v2.34; [71]), using an inverse Gamma as prior distribution for random and residual (co)variances (V = 1, nu = 1.002), 50,000 MCMC iterations, with 1,000 as initial burn-in and sampling every 10 iterations. Effective sample sizes for all model terms were always higher than 4,000, and MCMC traces were visually inspected to confirm convergence. We calculated the slope between egg volume and female helper number within and among mothers by dividing the corresponding covariance between egg volume and female helper number by the estimated variance in female helper number. We found that both the within- and among-mother slopes of female helper number on egg volume were positive (within mothers [95% CrI] = 0.015 [−0.004, 0.033]; among mothers [95% CrI] = 0.041 [−0.126, 0.214]). Mean posterior estimates (“Mean”) and their 95% credible intervals (“95% CI”) are provided. (DOCX) [file pbio.3002356.s030.docx]

**S22 Table.** Bivariate model of egg volume (cm^3^) and number of female helpers. This model was fitted in R using the MCMCglmm package (v2.34; [1]), using an inverse Gamma as prior distribution for random and residual (co)variances (V = 1, nu = 1.002), 50000 MCMC iterations, with 1000 as initial burn-in and sampling every 10 iterations. Effective sample sizes for all model terms were always higher than 4000 and MCMC traces were visually inspected to confirm convergence. We calculated the slope between egg volume and female helper number within and among mothers by dividing the corresponding covariance between egg volume and female helper number by the estimated variance in female helper number. We found that both the within- and among-mother slopes of female helper number on egg volume were positive (within mothers [95% CrI] = 0.015 [-0.004, 0.033]; among mothers [95% CrI] = 0.041 [-0.126, 0.214]). Mean posterior estimates (‘Mean’) and their 95% credible intervals (‘95% CI’) are provided.

|  | **Egg volume (cm^3^)** | | **Number of female helper** | |
| --- | --- | --- | --- | --- |
| **Fixed effect estimates** | **Mean** | **95% CI** | **Mean** | **95% CI** |
| Intercept | 3.788 | 3.463, 4.096 | 1.248 | 0.583, 1.914 |
| Rainfall | -0.844 | -1.434, -0.247 |  |  |
| Rainfall^2^ | -1.091 | -1.675, -0.486 |  |  |
| Heat waves | -0.015 | -0.024, -0.006 |  |  |
| Number of male helpers | 0.001 | -0.020, 0.022 |  |  |
| Clutch size | 0.003 | -0.041, 0.046 |  |  |
| Egg position | -0.067 | -0.095, -0.039 |  |  |
| **Variance component estimates** |  |  |  |  |
| Residual Variance | 0.034 | 0.029**,** 0.039 | 0.893 | 0.765, 1.008 |
| Residual Covariance | 0.013 | -0.003, 0.030 |  |  |
| Breeding season | 0.171 | 0.040, 0.376 | 0.804 | 0.183, 1.780 |
| Breeding season Covariance | 0.023 | -0.281, 0.370 |  | |
| Group ID Variance | 0.078 | 0.037, 0.130 | 0.372 | 0.134, 0.670 |
| Group ID Covariance | 0.001 | -0.075, 0.079 |  |  |
| Mother ID | 0.089 | 0.054, 0.134 | 0.414 | 0.194, 0.661 |
| Mother ID Covariance | 0.017 | -0.053, 0.087 |  | |

**Reference**

1. Hadfield JD. MCMC Methods for Multi-Response Generalized Linear Mixed Models in R: The MCMCglmm R package. J Stat Softw. 2010;33: 1–22. doi:10.1002/ana.22635
