# Supplementary material for: Mothers in a cooperatively breeding bird increase investment per offspring at the pre-natal stage when they will have more help with post-natal care
Source: PLoS Biol. 2023 Nov 9;21(11):e3002356. doi: 10.1371/journal.pbio.3002356 (PMC10635431; doi:10.1371/journal.pbio.3002356)
Supplement: S25 Table — This analysis used the full data sets from the egg volume model in Table 1 (490 measures from 271 clutches) and the maternal provisioning rate model in Table 3 (124 measures from 124 broods). Approximately 59 clutches/broods had measures for both egg volume and provisioning rate, while 277 clutches/broods had measures for only one of these 2 metrics. This model was fitted in R using the brms package (v2.34; [72]), using a default prior distribution for random variance components and a normal distribution of mean 0 and standard deviation 100 for fixed effects, and 4 chains of 50,000 MCMC iterations, with 25,000 as initial burn-in and sampling every 10 iterations in each case. Residual variation for maternal provisioning rates was fixed to 0.01 (i.e., no residual variation was left after accounting for differences among clutches). Convergence was assessed via Rhat values, which were always below 1.01 and visual inspection of chain traces. Response terms and fixed effect variables were mean centered and scaled by one standard deviation prior model fit to improve model convergence. “Rainfall” and “Rainfall2” were fitted as orthogonal polynomial, and their estimates are not back transformed in this table (i.e., units do not refer to the real data scale). Mean posterior estimates (“Mean”) and their 95% credible intervals (“95% CI”) are provided. (DOCX) [file pbio.3002356.s033.docx]

**S25 Table.** Bivariate model of egg volume (cm^3^) and maternal provisioning rates (feeds / hour) including the number of helpers as predictors. This analysis used the full data sets from the egg volume model in Table 1 (490 measures from 271 clutches) and the maternal provisioning rate model in Table 3 (124 measures from 124 broods). 59 clutches/broods had measures for both egg volume and provisioning rate, while 277 clutches/broods had measures for only one of these two metrics. This model was fitted in R using the brms package (v2.34; [1]), using a default prior distribution for random variance components and a normal distribution of mean 0 and standard deviation 100 for fixed effects, and four chains of 50000 MCMC iterations, with 25000 as initial burn-in and sampling every 10 iterations in each case. Residual variation for maternal provisioning rates was fixed to 0.01 (i.e., no residual variation was left after accounting for differences among clutches). Convergence was assessed via Rhat values, which were always below 1.01 and visual inspection of chain traces. Response terms and fixed effect variables were mean centered and scaled by one standard deviation prior model fit to improve model convergence. ‘Rainfall’ and ‘Rainfall^2^’ were fitted as orthogonal polynomial and their estimates are not back transformed in this table (i.e., units do not refer to the real data scale). Mean posterior estimates (‘Mean’) and their 95% credible intervals (‘95% CI’) are provided.

|  | **Egg volume (cm)** | | | **Maternal Provisioning rates (feeds / hour)** | | |
| --- | --- | --- | --- | --- | --- | --- |
| **Fixed effect estimates** | **Mean** | **SE** | **95% CI** | **Mean** | **SE** | **95% CI** |
| Intercept | 0.008 | 0.106 | -0.203, 0.224 | -0.014 | 0.150 | -0.345, 0.308 |
| Rainfall | -1.523 | 0.608 | -2.726, -0.341 | 1.055 | 1.115 | -1.124, 3.282 |
| Rainfall^2^ | -2.298 | 0.595 | -3.461, -1.119 | 2.763 | 1.060 | 0.669, 4.839 |
| Heat waves | -0.114 | 0.028 | -0.168, -0.060 | 0.201 | 0.100 | 0.010, 0.398 |
| Number of female helpers | 0.070 | 0.034 | 0.003, 0.137 | -0.183 | 0.078 | -0.337, -0.027 |
| Number of male helpers | 0.022 | 0.035 | -0.046, 0.091 | -0.038 | 0.081 | -0.195, 0.121 |
| Egg position | -0.119 | 0.025 | -0.168, -0.072 |  |  |  |
| Clutch size | 0.003 | 0.032 | -0.058, 0.065 |  |  |  |
| Brood size |  |  |  | 0.413 | 0.076 | 0.262, 0.563 |
| **Variance component estimates** |  |  |  |  |  |  |
| Residual Variance | 0.225 | 0.001 | 0.184, 0.271 | 0 | 0 | 0 |
| Breeding season | 0.002 | 0.001 | 0.001, 0.019 | 0.151 | 0.030 | 0.016, 0.643 |
| Group ID Variance | 0.013 | 0.009 | 0.001, 0.121 | 0.010 | 0.006 | 0.001, 0.086 |
| Mother ID | 0.591 | 0.006 | 0.394, 0.884 | 0.038 | 0.013 | 0.001, 0.198 |
| Mother ID Covariance | 0.072 | 0.067 | -0.033, 0.222 |  |  |  |
| Clutch ID | 0.029 | 0.004 | 0.001, 0.080 | 0.573 | 0.003 | 0.428, 0.765 |
| Clutch ID Covariance | 0.032 | 0.039 | -0.042, 0.112 |  |  |  |

**Reference**

1. Bürkner P-C. brms: An R Package for Bayesian Multilevel Models Using Stan. J Stat Softw. 2017;10: 1–28. doi:10.18637/jss.v080.i01
