# Supplementary material for: Mothers in a cooperatively breeding bird increase investment per offspring at the pre-natal stage when they will have more help with post-natal care
Source: PLoS Biol. 2023 Nov 9;21(11):e3002356. doi: 10.1371/journal.pbio.3002356 (PMC10635431; doi:10.1371/journal.pbio.3002356)
Supplement: S26 Table — This model contains data for a single clutch of 4 eggs. Model estimates, standard errors (SE), and their 95% confidence intervals (CI (95%)) are provided along with results from likelihood-ratio tests (χ2df = 1 and associated p-values) assessing the statistical significance of each predictor within the full model (i.e., a model containing all of the terms in the table below). Random effect standard deviation: “season” = 0.001 cm3, “group ID” = 0 cm3, “clutch ID” = 0.014 cm3, “mother ID” = 0.078 cm3. “Heat waves” (days above 35°C) and “Clutch size” were mean centered and scaled by one standard deviation prior model fit to improve model convergence. “Rainfall” and “Rainfall2” were fitted as orthogonal polynomial, and their estimates are not back transformed in this table (i.e., units do not refer to the real data scale). (DOCX) [file pbio.3002356.s034.docx]

**S26 Table**. Summary of results of a linear mixed model explaining variation in egg volume (cm^3^) using the extended dataset that includes low quality observations not containing information on egg position within the clutch (N = 906 eggs). This model contains data for a single clutch of four eggs. Model estimates, standard errors (SE) and their 95% confidence intervals (CI (95%)) are provided along with results from likelihood-ratio tests (χ^2^_df = 1_ and associated p-values) assessing the statistical significance of each predictor within the full model (i.e., a model containing all of the terms in the table below). Random effect standard deviation: ‘season’ = 0.001 cm^3^, ‘group ID’ = 0 cm^3^, ‘clutch ID’ = 0.014 cm^3^, ‘mother ID’ = 0.078 cm^3^. ‘Heat waves’ (days above 35˚C) and ‘Clutch size’ were mean centered and scaled by one standard deviation prior model fit to improve model convergence. ‘Rainfall’ and ‘Rainfall^2^’ were fitted as orthogonal polynomial and their estimates are not back transformed in this table (i.e., units do not refer to the real data scale).

| **Predictors** | **Estimates** | **SE** | **95% CI** | **χ ^2^_1_** | **p-value** |
| --- | --- | --- | --- | --- | --- |
| Intercept | 3.720 | 0.054 | 3.614, 3.826 |  |  |
| Rainfall | -0.418 | 0.263 | -0.933, 0.098 | 2.48 | 0.115 |
| Rainfall^2^ | -1.005 | 0.262 | -1.518, -0.492 | 14.37 | < 0.001 |
| Heat waves | -0.037 | 0.009 | -0.055, -0.019 | 14.78 | < 0.001 |
| Δ Number of female helpers | 0.010 | 0.009 | -0.006, 0.027 |  |  |
| µ Number of female helpers | -0.010 | 0.026 | -0.062, 0.041 |  |  |
| Δ Number of male helpers | 0.003 | 0.010 | -0.016, 0.022 |  |  |
| µ Number of male helpers | -0.024 | 0.032 | -0.086, 0.038 |  |  |
| Clutch size | -0.016 | 0.024 | -0.063, 0.032 |  |  |
| Δ Number of female helpers × Clutch size | 0.011 | 0.009 | -0.007, 0.028 | 1.44 | 0.229 |
| µ Number of female helpers × Clutch size | 0.009 | 0.014 | -0.018, 0.036 | 0.45 | 0.501 |
| Δ Number of male helpers × Clutch size | -0.007 | 0.013 | -0.033, 0.018 | 0.32 | 0.573 |
| µ Number of male helpers × Clutch size | -0.009 | 0.014 | -0.037, 0.019 | 0.37 | 0.545 |
