# Supplementary material for: Mothers in a cooperatively breeding bird increase investment per offspring at the pre-natal stage when they will have more help with post-natal care
Source: PLoS Biol. 2023 Nov 9;21(11):e3002356. doi: 10.1371/journal.pbio.3002356 (PMC10635431; doi:10.1371/journal.pbio.3002356)
Supplement: S31 Table — Model estimates, standard errors (SE), and their 95% confidence intervals (CI (95%)) are provided in the link scale (i.e., “log”) along with results from likelihood-ratio tests (χ2df = 1 and associated p-values) assessing the statistical significance of each predictor within the full model (i.e., a model containing all of the terms in the table below). Random effect standard deviation: “season” = 0.090 feeds/hour, “group ID” = 0 feeds/hour, “clutch ID” = 0.240 feeds/hour, “mother ID” = 0.084 feeds/hour, observation level = 0.341 feeds/hour. “Heat waves” (days above 35°C) and “Brood size” were mean centered and scaled by one standard deviation prior model fit to improve model convergence. “Rainfall” and “Rainfall2” were fitted as orthogonal polynomial, and their estimates are not back transformed in this table (i.e., units do not refer to the real data scale). (DOCX) [file pbio.3002356.s039.docx]

**S31 Table.** Summary of results of a generalised linear mixed model (Poisson residual distribution) explaining variation in maternal provisioning feeds, including log observation time as an offset and, thus, effectively modelleding maternal provisioning rate (feeds / hour; N = 251 days of maternal provisioning observations). Model estimates, standard errors (SE) and their 95% confidence intervals (CI (95%)) are provided in the link scale (i.e., ‘log’) along with results from likelihood-ratio tests (χ^2^_df = 1_ and associated p-values) assessing the statistical significance of each predictor within the full model (i.e., a model containing all of the terms in the table below). Random effect standard deviation: ‘season’ = 0.090 feeds / hour, ‘group ID’ = 0 feeds / hour, ‘clutch ID’ = 0.240 feeds / hour, ‘mother ID’ = 0.084 feeds / hour, observation level = 0.341 feeds / hour. ‘Heat waves’ (days above 35˚C) and ‘Brood size’ were mean centered and scaled by one standard deviation prior model fit to improve model convergence. ‘Rainfall’ and ‘Rainfall^2^’ were fitted as orthogonal polynomial and their estimates are not back transformed in this table (i.e., units do not refer to the real data scale).

| **Predictors** | **Estimates** | **SE** | **95% CI** | **χ ^2^_1_** | **p-value** |
| --- | --- | --- | --- | --- | --- |
| Intercept | 1.892 | 0.080 | 1.736, 2.049 |  |  |
| Rainfall | 0.693 | 0.632 | -0.546, 1.931 | 1.19 | 0.275 |
| Rainfall^2^ | 0.959 | 0.581 | -0.179, 2.097 | 2.67 | 0.102 |
| Heat waves | 0.090 | 0.044 | 0.004, 0.176 | 3.95 | 0.047 |
| Number of female helpers | -0.078 | 0.030 | -0.137, -0.019 | 6.46 | 0.011 |
| Number of male helpers | -0.001 | 0.037 | -0.073, 0.072 | 0.00 | 0.981 |
| Brood size | 0.184 | 0.039 | 0.107, 0.260 | 21.54 | < 0.001 |
