# Supplementary material for: Mothers in a cooperatively breeding bird increase investment per offspring at the pre-natal stage when they will have more help with post-natal care
Source: PLoS Biol. 2023 Nov 9;21(11):e3002356. doi: 10.1371/journal.pbio.3002356 (PMC10635431; doi:10.1371/journal.pbio.3002356)
Supplement: Attachment — Submitted filename: Response to Reviewers - PBIOLOGY-D-21-03064R1.pdf [file pbio.3002356.s040.pdf]

Point-by-point response to reviewers' comments - PBIOLOGY-D-21-03064R1

Reviewer #1:
This manuscript discusses patterns of maternal investment in eggs in a species of African cooperative-breeder, the white-browed sparrow-weaver, on which the authors have been gathering data for about a decade. The propose that when there are female helpers, mothers reduce their post-hatching workload, but lay larger eggs. They relate this to hypotheses from other animals, and propose a front-loading variant of these hypotheses, which posits (I think) that mothers invest early and relax later.

While these data and the discussion are of considerable interest, I found aspects of the methods and results unapproachable, and that the introduction and discussion failed to integrate the results properly with the relevant literature on cooperative breeding.

> Authors' Response: Thank you for your interest in the manuscript. We have now revised our statistical methods (following the recommendations of R3), and the new approach has allowed us to markedly simplify the presentation of our findings, which remain unchanged. We have also refined the theoretical framework of the manuscript as requested. We hope that this revision has addressed your concerns and would be happy to refine the manuscript further if desired.

Turning to the latter problem first, their description of load-lightening is awkward and confusing, as in extensive prior literature, the term is usually applied to the ability of the female to reduce her workload post-laying, which can provide her with an ability to re-nest faster, or be more likely to survive to breed again. Past compilations (e.g., Hatchwell Am Nat, Legge Anim Behav) have shown that this is very likely in low fecundity species where partial or complete brood failure through starvation is rare. Hence in many such cooperative breeders, groups with a big workforce can reduce their care without interfering with the total (corporate) delivery of food to the brood. By contrast, where nestling starvation is rife, the breeding pair tends not to load-lighten, but instead uses the enhanced workforce to boost nestling survival. In this paper, the egg focus means this conventional explanation of the nestling phase is hardly mentioned, though the dramatic reduction of feeding with helper number is far greater than any effect of egg size.

> Authors' Response: We appreciate the point and have now adjusted our introduction to the 'load-lightening hypothesis' for pre-natal maternal investment to clarify the terminology (see L73-80). We first explain that the recent literature uses the term 'load-lightening' to describe helper-induced reductions in maternal investment, whether they occur post-natally (i.e., at the chick-feeding stage; the context in which the term was first used, as emphasised above) or pre-natally (i.e., at the egg stage). This keeps our terminology in line with the recent literature examining maternal responses to helping at both the post- and pre-natal stages (e.g., "[…] *Load lightening could also occur at the egg stage* […]" p941 in Russell *et al*. 2007 *Science*; the first paper to really highlight the possibility of maternal load-lightening at the egg stage). We have also stressed further that here we are considering the "load-lightening hypothesis for the adjustment of maternal *pre-natal* investment…" (see L76-80).

Data on the total feeds etc are not presented to assess whether the reduction in female feeds is allowed by the helper contributions.

> Authors' Response: We have now adjusted the background text (L147-150) to clarify this aspect of wider context; we have already shown (Capilla-Lasheras *et al*. 2021 *Science Advances*) that broods reared by mothers with more female helpers are fed at higher rates overall (i.e., when the contributions of all group members are combined; perhaps what the reviewer means by 'total feeds etc'). So, taking those results together with our findings here, mothers do indeed appear to be engaging in a 'partial compensation' strategy within the post-natal stage: reducing their own contributions to chick feeding when helped, but not so much as to fully offset the post-natal contributions of their helpers (something that we highlight in e.g., L147-150 and L348-350).

There is also no data on brood reduction.

> Authors' Response: We would be happy to respond to specific suggestions in this regard, but it is not clear to us what specific data the reviewer would like to see presented regarding brood reduction and what particular concern this would address. If the reviewer could clarify, we'd happily address their concern.

This point drives home a second feature of the manuscript. Because of the use of AIC methods, the standardisation procedures, and the complete lack of units for effect sizes, it is difficult to assess the result.

> Authors' Response: Thank you for highlighting the potential for greater clarity in our statistical methods and reporting. In response to this and R3's suggestions, we have now changed the statistical approach in the main text (we now employ a simpler 'full model' approach using likelihood ratio tests with P values for significance testing; e.g., Tables 1-4) and provide non-standardised model coefficients (with units) for helper effect terms.
>
> Embracing the full model approach has not altered any conclusions. It has also allowed us to simplify and clarify our statistical methods and the presentation of our statistical outcomes (e.g., removing the additional complication previously entailed in using the 'nesting rule'). The original AIC-based model selection tables are now provided only in the supplementary materials to illustrate that our findings are robust to the use of alternative statistical modelling approaches.

Fig 1 shows that there is extreme variation in egg volume, spanning from about to 2.8 to 4.5cm^3, so max is 1.7 (or 60% greater than min). Fig 2a suggest that the effect of going from 0 to 5 helpers is about and possibly less than 0.1m^3, or about 6% of the variance, which is hardly compelling. The reader should not have to work this out for themselves. While AIC methods are the vogue, rendering the effects into realistic estimates seem often to go out the window.

> Authors' Response: To facilitate the biological interpretation of our results without reference to the figure (which illustrates the relationship and presents the full data spread and predicted effect with a y axis that does have units), we now present all

of our model-estimated effect sizes for the helper number effects and our figures in the same scales, with appropriate units. Our original use of AIC would not have affected the biological interpretation of model estimates, but switching to a full-model based approach in this revision has simplified our statistical reporting in a way that should enhance clarity.

We do agree that the detected within-mother effect of female helper number on egg volume is modest, and we explicitly acknowledge this in the manuscript (L291).

I also found problems with the presentation of the methods. Male numbers are present in some of the models and the main text is very confusing as to the reasons why these models are excluded. The text (l. 247-8) states: 'male helper number' effect was 0.93 AIC points below the top model (Table 2) and would be rejected under the nesting rule (see Table 2 legend)'. However, when you go to that table legend it advises on l 780-1 'Only the top model would be retained under the nesting rule ('Retained' [50]; see methods).'

Authors' Response: Switching to the suggested 'full model approach' has now addressed this concern, by reducing the complexity of the statistical reporting which should facilitate interpretation. For example, you will see that the male helper number and female helper number terms are both present as predictors within all four of the key full model output tables in the main paper (Tables 1-4).

The methods on l. 356-358 state 'We gave consideration to models with ΔAIC values < 6 [50,51] and subsequently reduced this Δ6 top model set by applying the 'nesting rule' described in [50]'. Reference 50 is Richards SA. Dealing with overdispersed count data in applied ecology. J Appl Ecol. 2008;45: 218-227. There is only one explicit use of the term 'nesting rule' in this paper, but it is not defined. The definition of nesting in the paper appears to be 'identifying which models are special cases of others' but I am afraid that was not much help to me, as it seems that models with male helpers as an explanatory variable are not special cases of models with female helpers. So this left me confused. I doubt anything incorrect has been done, and I am inclined to suspect not, but a reader should not be sent on a merry-go-round through the manuscript to find a reference which is difficult to apply to the current dataset.

Authors' Response: Thank you for highlighting this. Switching to the suggested 'full model approach' in the main text has addressed this point too, by removing the need for the nesting rule. Doing so leaves our overall findings and conclusions unchanged.

While the cited paper shows that the nesting rule offers a conservative approach to the interpretation of model comparison outcomes (and we should also have cited Arnold 2010 and Richards, Whittingham & Stephens 2011; apologies for the oversight), we do appreciate that its use creates challenges with interpretation for those unfamiliar with the approach. As such, the complementary AIC-based model selection tables that we now provide within the supplementary materials no longer have the nesting rule applied (allowing the reader to apply it themselves if that is their preference in this context). Our findings are the same regardless of the statistical approach taken.

For these reasons, I found the manuscript problematic at the moment, and I have not gone on to a full review, as that would have to be done after these problems are corrected.

> Authors' Response: Thank you for your feedback. We hope that the newly revised version with its simplified and clarified statistical methods and reporting, and confirmation of the key statistical outcomes using the full-model approach as well as the original AIC-based approach, leaves the reviewer more confident in the findings presented.

Reviewer #2:
Mothers front-load their investment to the egg stage when helped in a wild cooperative bird

Review

Major revision
This manuscript investigates how mothers in cooperative breeders could adjust their pre-natal investment relative to the amount of post-natal help they receive. This is an important study of broad interest. First, because the outcome will directly help us better understand the evolution of cooperation. Second, because such investment could conceal or reinforce the impact of post-natal helper contributions in cooperative breeders, a critical component of most investigations of benefits of cooperative breeding. The study is only the second to investigate such maternal strategies using a powerful approach to detect maternal plasticity (within-subject centring), applying rigorous analyses of a long term dataset on prenatal and postnatal investment. The study species is perfectly suited, as maternal strategies can be related to numbers of male and female helpers, whereby these helpers contribute differently, with only females providing additive care. This provides a powerful opportunity to disentangle hypotheses on maternal investment strategies. Strengths of the manuscript are the long term dataset, the comprehensive analyses also including climatic effects, the detailed understanding of the study species. The manuscript is mostly clear and well written. I have a few minor comments, and one major comment: the focus on egg size only in terms of framing, presentation and statistical analyses. A more nuanced framing and balanced approach would enhance the manuscript. Additionally, I suggest some analyses in addition to those in the supplement, and a thorough revision of the Discussion to improve the writing and enhance the impact of the message. Details below.

> Authors' Response: Thank you very much for your interest in the manuscript, for highlighting its key strengths and for your useful detailed feedback throughout. We feel that tending to these comments has significantly improved the manuscript. Please see below for a point-by-point response to your review.

Major comments
Framing. The manuscript is framed around egg size as pre-natal investment (introduction third and fourth paragraph). It is however only one component - pre-natal female investment consists of clutch size and egg volume combined. Some studies show trade-offs between the two, while they can also co-vary. The introduction should consider evidence for clutch size modifications also, and consider both equally as pre-natal investment.

> Authors' Response: Thank you, we do agree about the importance of clutch size (alongside egg size) in this context. To highlight that we have directly attended to the issue of clutch size, we have now moved our parallel analyses of clutch size from the supplemental materials to the main text as requested.
>
> When considering alternative framings for the introduction, we chose to focus our introduction from the outset (paragraph 1) on understanding variation in pre-natal investment *per offspring* (in this case egg volume) because this is the primary

motivation of the paper, and because most of the theoretical work (and associated hypotheses) to date regarding helper effects on pre-natal investment has focused principally on egg size. This focus is also appropriate for our model system, as our study species lays a small clutch (of just two eggs) whose size rarely varies and is unrelated to helper numbers (as we show within the manuscript). We therefore feel that, given the need for quite a complex setup already (to convey the relevant theory and hypotheses for helper effects on egg size alone), attempting to also explain the various mechanisms by which helpers could affect the optimal clutch size could yield an unwieldy theoretical framework that was more complex than required to both set up our study and interpret our findings. As such, it would be our preference to retain the current theoretical framework for the paper, with its emphasis on egg size.

Nevertheless, given the importance of considering clutch size (as we do in our analyses) when studying maternal pre-natal investment per offspring, we have now made revisions to the Introduction to more explicitly and earlier highlight (i) the potential importance of parallel changes in clutch size (L64-69), and (ii) the evidence of maternal plasticity in clutch size found by Lejeune *et al.* 2016 (L64-69 and L128; as is rightly highlighted below). We have also moved our analyses of clutch size into the main text (see below), highlighted more clearly up front the significance of our clutch size results for understanding variation in pre-natal investment per offspring (L177-180), and carried out the additional bivariate models requested (see below). We believe that the manuscript deals more effectively with the issue of clutch size now, providing clarity regarding the focal hypotheses relevant to understanding how helpers affect pre-natal investment per offspring, but at the same time giving due attention to the possibility that other components of maternal pre-natal investment (i.e., clutch size) change in parallel.

Analysis. Similarly, to analyse egg size separately from clutch size and only present egg volume as the main result is not best. Both these analyses belong in the main manuscript so that the reader can more readily assess the evidence for total pre-natal investment (and it is more evident that both were analysed by the same methods). Possibly Fig. 1 could be moved to supplement, if space needed to be saved, but overall, incorporating clutch size results in the main ms should not take up too much space, since the extended results can remain in the supplement.

Authors' Response: We agree and have now moved the clutch size analyses to the main paper results section as requested (see L239-251).

Moreover, Lejeune *et al*. 2016 analysed a very similar dataset testing the same hypotheses (albeit framed somewhat differently) in a similar species (with similarly small clutch size), using a multivariate analysis combining egg size and volume as bivariate response. This seems like a very good approach. If this approach is not possible or not preferable for some reason, this should be explained in the manuscript.

Authors' Response: We agree that the Lejeune *et al*. bivariate approach has merit (being particularly informative when clutch size and egg volume are correlated) and can confirm that conducting the suggested bivariate model confirms our existing findings; egg volume and clutch size are not correlated in our study species,

either among or within mothers (Response Table 1) and female helper numbers predict egg volume but not clutch size:

Response Table 1. Correlations between egg volume and clutch size within and between mothers after applying the suggested bivariate model with egg volume and clutch size as response variables (following R2's comment). Note that the 'covariance'/'correlation' terms are always close to 0 and their 95% credible intervals (95%CrI) overlap zero, indicating no evidence for correlations between these two traits (as shown in our analyses in the main text).

		**Egg volume**	**Clutch size**
**Between mothers**	**Egg volume**	Variance (95%CrI) = 0.589 (0.350, 0.836)	Covariance (95%CrI) = -0.061 (-0.166, 0.052)
	**Clutch size**	Correlation (95%CrI) = -0.335 (-0.873, 0.219)	Variance (95%CrI) = 0.082 (0.001-0.196)
**Within mothers**	**Egg volume**	Variance (95%CrI) = 0.203 (0.168, 0.238)	Covariance (95%CrI) = 0.009 (-0.043, 0.057)
	**Clutch size**	Correlation (95%CrI) = 0.029 (-0.107, 0.54)	Variance (95%CrI) = 0.741 (0.642, 0.837)

The main argument in Lejeune *et al*. to analyse egg size and clutch size with this bivariate approach was to account for correlations between those traits and investigate the existence of trade-offs between them. In our case, we included clutch size as a predictor of variation in egg volume, and hence our analysis also allows for a potential correlation (i.e., trade-off) between those traits. As our existing models indicate that clutch size is not correlated with egg volume (Table 1), and we also found no evidence of helper effects on clutch size (L239-251), we feel that our existing approach already covers the necessary ground, without the added complication of the reader needing to navigate a bivariate approach. The above confirmation could certainly be included in supplementary materials though if desired.

Discussion. Overall the discussion is in places hard to follow and does not quite grab the reader: the writing can be clearer and more concise, and the structure tightened up to improve the impact of the message

Authors' Response: Thank you for the suggestions. We have revised the discussion with a view to enhancing clarity, with particular attention to your specific comments below.

Minor comments
Introduction
L. 123-124: I am not sure that Langmore *et al.* 2016 did actually use the same approach. Lejeune *et al*. 2016 used within-subject centring, which does indeed partition the variance in within- and between-female effects as used here, but Langmore used mixed models with female ID as random term, combined with a climate window approach to find the best predictor climate.

Authors' Response: We have double checked the analysis in Langmore *et al*. 2016 to confirm that they did indeed partition variation in abiotic conditions (temperature) into its within- and among-subject components when investigating the effects of abiotic conditions (hence our acknowledgement of this). However, we do agree that they did *not* use this approach to distinguish the effects of within-and among-subject variation in helper numbers (as our study does). Our intention was to acknowledge their use of this statistical method, even though it was not applied to helper effects *per se*. Our phrasing here is therefore consistent with the contents of Langmore *et al*. 2016 (see L112-114 and L129-130).

L. 124-125: this wording is a little awkward and misleading, as the study by Lejeune *et al*. 2016 did test for maternal plasticity in egg volume, in a similar system (male and female helpers have different effects, only female helpers provide additive care): it's just that the females did not seem to adjust egg size according to helper number, which does not support the predictions of either the load-lightening or differential allocation hypothesis. However females breeders did increase clutch size when they had more helpers (of either sex), which supports the differential allocation hypothesis, whereas female helpers enabled more rapid re-nesting.

Authors' Response: We agree that our wording was awkward here, as one could have read this sentence in isolation and inferred that the partitioning approach had *only* been used to investigate maternal plasticity in egg volume according to abiotic conditions, which is of course incorrect (e.g. in the previous sentence we highlighted its use by Lejeune *et al* to test for plasticity in egg volume according to helper numbers). We have therefore adjusted the phrasing to clarify, and adjusted the previous sentence to also report Lejeune *et al*'s finding of maternal plasticity in clutch size (alongside our existing acknowledgement of their use of this technique to test for egg volume plasticity, which was not found). The new phrasing now covers these points more clearly (see 124-129; we also acknowledge Lejeune *et al*'s clutch size plasticity result earlier in the Introduction [L64-69] and more explicitly in the discussion [see below]).

l. 171-174: it would be informative to know exactly the level of replication across mothers, how many contributed only a single data point, what is the distribution. Especially given the modest sample size for provisioning (modest for the complex analyses applied here, with many females contributing only 1 brood).

Authors' Response: Agreed. We have now included these details in the manuscript (L564-565) and added two new supplementary figures with the distribution of eggs and provisioning observations per mother (Figure S6 and Figure S7).

Results
l. 203-204: could this not be formally tested using equation (3) in Van de Pol and Wright (2009)?

Authors' Response: Yes, indeed. Thank you for this suggestion. We have now conducted this test and included the associated methods (L643-645) and results (L214-221 and L267-269) in the manuscript.

As expected based on the within- and between-mother slopes originally reported, and consistent with our interpretation of our findings, applying Equation 3 in van de Pol & Wright to formally test for differences between them does indeed provide no statistical evidence for such a difference, either for the effect of female or male helper numbers.

l. 213 and 245: The power to detect within- and among-individual effects also depends on the variation, what is the distribution of number of male and female helpers within and among mothers? If this is not extremely similar, a power analysis would be helpful to compare whether the likelihood of detecting male and female effects is similar?

Authors' Response: We have now added a new figure to show that the distributions of female and male helpers (both within and among mothers) are indeed very similar in the relevant data set (see Figure S6 – also included below). Given the similarity of these data distributions, the power to detect their effects must have been very similar.

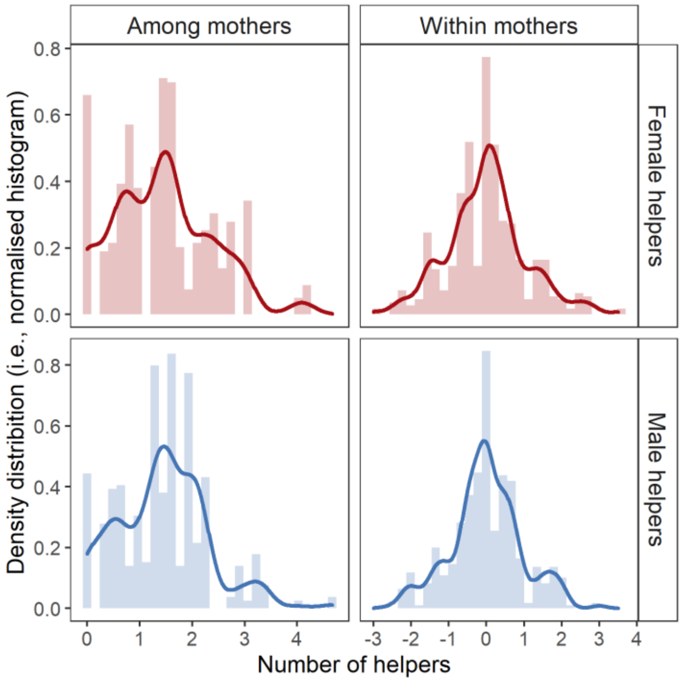

L. 221-223, 250-251 - could the difference in effect size somehow be formally tested by an interaction with sex? (I suspect not, but worth thinking about?)

Authors' Response: Yes, indeed. We have now managed to formally test whether the female and male helper effects here were significantly different from each other in the analyses of both egg volume and maternal provisioning rates using a method that we hadn't previously encountered (see results in L227-231 and L274-278; and methods in L616-618). These analyses confirm that (as one would expect on the basis of the reported effect sizes and standard errors) while the magnitude of the point estimates for the effects of female helper numbers were numerically larger than those for male helper numbers for both maternal traits (e.g. for within-mother variation, 0.019 vs 0.008 cm^3/helper respectively in the egg volume analysis, and -0.559 vs 0.006 feeds / hour / helper respectively in the provisioning analysis), they

weren't statistically *significantly* larger. We have now included these additional test outcomes within the results text for both egg volume (L227-231) and provisioning rate (L274-278)

l. 225, 257-258, Climatic analyses: see my comments for the supplementary material below

Authors' Response: We have replied to your specific comments below.

Discussion
To improve the structure could be partially achieved by streamlining the paragraphs, starting each with a clear brief topic sentence that indicates the take-home message, to avoid repetition, and to make arguments more concisely.

Authors' Response: As well as replying to your specific points below, we have streamlined the discussion accordingly.

Paragraph structure:
For example. l. 319-335 "It is conceivable that the core rationale of the differential allocation hypothesis nevertheless can account for the pattern of egg investment observed here, as the biological processes and/or parameter space explored in the theoretical work to date [9] might not capture all relevant aspects of the biology at play." the message seems to be that 'the differential allocation hypothesis could explain the pattern of egg investment observed here"? yet the previous paragraph seems to say the same? 'The 'differential allocation hypothesis' as applied to cooperative breeders does predict the pattern of egg investment observed here'. This not only makes the discussion hard to follow but also suggests these two paragraphs could be combined into one paragraph making the point more succinctly.

Authors' Response: We appreciate the point and have now edited these two paragraphs into a single paragraph (focussed on the extent to which our findings fit with the predictions of the differential allocation hypothesis) to capture the rationale more clearly (see L327-356).

Avoid starting paragraphs with generic openers (It is conceivable that the core rationale; While it may ultimately prove possible to reconcile…) and long statements.

Authors' Response: Thank you for this suggestion. We have now removed these openers in the discussion.

Repetition: For example, the hypotheses are nicely explained in the introduction, so the discussion can be briefer, and predictions do not need to be much repeated.

Authors' Response: We have addressed specific comments below and revised our discussion to be less repetitive. Thank you for these comments.

The description of your results is repeated a number of times (e.g. in the first paragraph, l. 263-265, l. 265-267, l. 270, l. 274-276), which could be streamlined. There also are repeated versions of the assertion 'this is the first time that plasticity in egg size relative

to helper number has been demonstrated'. (e.g. l. 263-264, l. 283-284, l. 380-381). This repetition nor the assertion are necessary. For example l. 269 - 273, this information is repeated l. 311-314.

Authors' Response: We agree that there was scope to reduce repetition and so have sought to do so wherever appropriate (e.g., we have removed the 'first time' novelty statements from L306-308 and L402-403 [formerly 283-284 & 380-381] and the mention of the Iberian magpie results from L294-297). Where the suggested areas of repetition refer to pairs of points that differ in content or in which the second use removes ambiguity at a later point in the text we have retained both points.

l. 279-280: delete

Authors' Response: This sentence highlights one of the key implications of our findings (and hence builds on the end of the previous sentence), so we are keen to retain it. We have removed the distracting 'Notably,' from the start of it though.

l. 282: the results of Lejeune here should be acknowledged and better discussed, while yours is the first study showing plasticity in egg volume, their study explicitly tested it, in a similar species, but did not find it; instead previously plasticity in clutch size was demonstrated. This is a different front loading strategy (offspring number/quality), I would argue?

Authors' Response: Thank you for this observation. In the very next sentence we noted the important contribution of Lejeune *et al*'s use of the partitioning approach to study helper effects on egg size (now L308-310), a point that we also stressed in our Introduction. So Lejeune *et al*'s approach and findings regarding egg size should hopefully be clear to the reader. We do very much agree that a fuller treatment of Lejeune *et al*'s clutch size results was needed though, and so we've made edits to highlight these both in our Introduction (see replies above) and our discussion (e.g., L318-320). We also now highlight that this plastic increase in clutch size when helped could itself be considered another form of maternal front-loading, that could arise via similar mechanisms to those discussed (see L380-384); thank you for highlighting this point.

l. 363-365: delete

Authors' Response: We consider this to be a main message of the manuscript and so have retained this sentence.

l. 365-278: this paragraph has as take home message that any egg volume adjustments can distort helper effects on offspring quality (conceal or amplify)? Is it worth addressing explicitly under what circumstances might females positively or negatively adjust egg volume?

Authors' Response: We agree that this is an interesting topic and so do speculate about the conditions that may lead to maternal front-loading or otherwise (e.g., L370-384). We would prefer not to repeat this speculation or delve too much deeper, given the length of the discussion as it stands.

Supplementary material,
Section A
Sliding window approaches with continues dependent variables, especially if these follow awkward distributions and sample size is a little limited, seem to have a tendency to generate results that are difficult to interpret biologically. I appreciate that the authors were conservative in their approach, using 4 day windows and large AIC cut-off. The result for the effect of heat waves on egg volume is very interesting, and seems robust and sensible. However, looking at the rainfall distribution and results for rainfall, these are a little awkward, rainfall is almost bimodal (yes/no; little/lots), as it commonly is. A quadratic effect of rainfall seems feasible, but why only for rain that occurs 44-49 days before laying for egg volume and 61-78 days before laying for provisioning - do these time spans make biological sense? I would imagine that strong rainfall during laying would depress egg volume, through effects on insect availability? Even if large amounts of rainfall can affect the woven structure or flood or resources, the support is not very strong, and the sample size, especially for provisioning, not very large? I could not quite work out if the authors assessed if inclusion or not of rainfall affected the eventual conclusions on helper effects on egg volume, but if so, perhaps coding rainfall effects more simply could be attempted to verify this result. This would be reassuring to know.

Authors' Response: Thanks for this suggestion and we appreciate the point. As requested, we have done follow-up analyses to address these points.

Rainfall in the Kalahari Desert triggers marked time-lagged vegetation changes and associated lagged increases in insect abundance, both of which are likely to increase resource availability for sparrow-weavers (as they eat seeds as well as insects). As such, it was no surprise to us to see the sliding window approach identify time-lagged and generally positive effects of rainfall on both egg size and provisioning rate. Indeed, our wider work has identified beneficial effects of rainfall on a range of fitness-related traits in this system: laying probability (Wood *et al*. 2021 *Mol Ecol*), clutches laid per season (this manuscript), nestling survival to fledging (Capilla-Lasheras *et al.* 2021 *Science Advances*) and fledglings reared per season (Wood *et al*. 2021 *Mol Ecol*). Rainfall also predicts the telomere length dynamics of adults (Wood *et al*. 2021 *Mol Ecol*) and offspring; hatchlings from eggs laid after higher rainfall periods have significantly longer mean telomere lengths (Wood *et al.* 2017; *PhD thesis*). The latter finding is consistent with the generally positive relationship reported here between rainfall and egg volume (as increased resource availability *in ovo* may facilitate self-maintenance during early development).

That said, we do appreciate the reviewer's caution regarding (i) the complexity of the detected quadratic response of egg volume to rainfall, and (ii) the short time windows that the sliding window approach has suggested are relevant in each case. While our sample sizes are quite large for data of this kind, they could nevertheless leave it challenging to identify the appropriate shape and time window for the rainfall effect. We have done two sets of analyses to allay these concerns.

First, regarding the complexity of the detected quadratic rainfall fit for egg volume, we can confirm that the female helper number effect sizes have not been affected by including the sparser data for higher rainfall values or the resulting quadratic

rainfall fit. When we just fit a linear rainfall relationship to the egg volume data from below the peak in the detected quadratic rainfall relationship (i.e., for rainfall levels < 60mm; see Figure S3a), where the data density is higher, the effect sizes for the effects of both female helper number (i.e., prior to partitioning) and Δ female helper number (following partitioning) on egg volume are virtually unchanged:

Female helper number effect size ± SE *with full data set and quadratic rainfall fit* (as per Table 1 in the manuscript) = +0.018 ± 0.009 cm^3/female helper; and then *for rainfall < 60mm and linear rainfall fit* = +0.019 ± 0.009 cm^3/female helper (the associated p value for female helper number drops slightly from 0.038 to 0.026).
Δ female helper number effect size ± SE *with full data set and quadratic rainfall fit* (as per Table 2) = +0.019 ± 0.009; and then *for rainfall < 60mm and linear rainfall fit =* +0.019 ± 0.009 cm^3/female helper (the associated p value for female helper number drops slightly from 0.037 to 0.035).

Second, we can confirm that the effect size estimates for the detected effects of female helper numbers on both egg volume and maternal provisioning rate also remain virtually unchanged if, despite the expectation of rainfall effects on both traits (see above), we do not allow rainfall to predict either trait:

For the **egg volume** analysis, removing rainfall entirely from the model leaves the effect size estimates for both female helper number (i.e., prior to partitioning) and Δ female helper number (following partitioning) virtually unchanged:
Female helper number effect size ± SE *with* rainfall (as per Table 1) = +0.018 ± 0.009 cm^3 / female helper; and *without* rainfall = +0.017 ± 0.009 cm^3 / female helper (the associated p value rises slightly from 0.038 to 0.063).
Δ female helper number effect size ± SE *with* rainfall (as per Table 2) = +0.019 ± 0.0090 cm^3 / female helper; and *without* rainfall = +0.017 ± 0.0093 cm^3 / female helper (the associated p value rises slightly from 0.037 to 0.063).

For the **maternal provisioning rate** analysis, removing rainfall entirely from the model again leaves the effect size estimates for both female helper number and Δ female helper number virtually unchanged:
Female helper number effect size *with* rainfall = -0.457 ± 0.195 feeds / hour / female helper (as per Table 3); and *without* rainfall = -0.454 ± 0.202 feeds / hour / female helper (the associated p value rises slightly from 0.020 to 0.026).
Δ female helper number effect size *with* rainfall = -0.559 ± 0.269 feeds / hour / female helper (as per Table 4); and *without* rainfall = -0.530 ± 0.279 feeds / hour / female helper (the associated p value rises slightly from 0.040 to 0.060).

As we have good biological reasons to expect lagged rainfall effects on both maternal traits (see above), and as our simulations suggest that the significant lagged rainfall effects detected for both traits have only a modest probability of having arisen by chance, we would expect the most reliable inferences about helper effects to stem from analyses that allow for the significant rainfall effects identified by the sliding window approach. We have therefore continued to include these predictors in our full models, but have also included all of the information above within the supplementary materials (see L113-133 and L159-166 in

Supplementary Material A), to reassure the reader that doing so has had no appreciable effect on the effect size estimates for the female helper number terms.

Section C
A more straightforward analysis to address whether egg volume can be attributed to carry-over effects of past help would be to run the same models but with helper numbers of the previous attempt, including time since the last breeding attempt as co-variate perhaps? Why was this not attempted?

Authors' Response: Thanks for this suggestion. We carried out the suggested additional analysis and found no effects of the number of helpers in the previous breeding attempt on egg volume (Supplementary materials F). This new analysis complements our existing findings in Supplementary materials C, D and E, which together find no evidence of carry over effects of helping on egg volumes in later breeding attempts.

Reviewer #3:
[identifies himself as Joel Pick]
I think this paper addresses an interesting topic, and adds to the emerging picture of how pre and postnatal maternal investment vary in cooperative systems.

However, I have a serious concerns about the analysis. I started my review with the methods and results sections, and based on my concerns I have restricted my review to these sections.

> Authors' Response:
>
> Dear Joel,
>
> Thank you for the time spent reviewing our paper.
>
> We welcome your suggestions and have fully embraced the key suggestion of using a 'full model' approach in place of AIC-based model selection, have demonstrated that all of our findings are robust to using this approach and now employ this approach throughout. We have relegated the original AIC-based model selection outcomes for each analysis to supplementary materials, where they serve only to confirm that our full model findings presented within the main paper are robust to using AIC-based model selection instead (for any readers with a preference for that approach). We agree that there are reasons to consider the proposed 'full model approach' more conservative in this context.
>
> Employing a full model approach also comes with the added benefit of its outcomes being more straightforward to present to readers. We therefore now draw our inferences exclusively using the full model approach, and as requested our main model tables now report the effect sizes, SEs and CIs for all predictors tested in this context, along with the outcomes of likelihood ratio tests conducted in this context. Embracing the full model approach has also allowed us to simplify and clarify our statistical methods and the presentation of our findings (e.g., by removing the complexity that previously arose from applying the 'nesting rule').
>
> Please, see below for a point-by-point response to your revision. Thanks again for your help strengthening the manuscript.

Main points:
1. Generally I am not a fan of model selection using AIC, but I do see a place it. However, I do not think it is appropriate for this kind of data set and should not be used in this way. I would strongly suggest that a single clear, biologically coherent model is run for each trait/question, and interpretation is based on this one model. I see no benefit to using extensive AIC model selection in this context, only potential issues. I can see that excluding interactions that are not significant/different based on AIC (these turn out to be essentially equivalent – see, for example, Murtaugh 2014 Ecology, Figure 2) make model interpretation easier, but I see no justification for removing any main effects from the model.

I would highly recommend that clear statistical justification for this approach is given, over just running a single model and presenting all the parameter estimates, their uncertainty and some measure of confidence in the estimates (e.g. p-values, likelihood ratios etc).

> Authors' Response: Thank you very much for your suggestion. We have confirmed that all of our findings are indeed robust to employing the suggested 'full model approach' in place of AIC-based model selection, and have now switched to using the full model approach throughout the manuscript as suggested.
>
> We agree that there are good reasons to consider the proposed 'full model approach' a more conservative procedure. Employing a full model approach also comes with the added benefit of its outcomes being more straightforward to present to readers. We therefore now draw our inferences solely using the full model approach, and our main model tables now report the effect sizes, SEs and CIs for all predictors tested in this context, along with the outcomes of likelihood ratio tests conducted in this context. Embracing the full model approach has also allowed us to simplify and clarify our statistical methods and the presentation of our statistical outcomes (e.g., by removing the need for the 'nesting rule').
>
> For the benefit of readers with a preference for AIC-based model selection (and to illustrate the robustness of our findings to the choice of modelling approach), we now (i) highlight within the methods (L603-613) and at the end of the legends for each of the main model output tables (Tables 1-4) that the findings from each of these main models are also robust to the use of AIC-based model selection, and (ii) present the relevant AIC-based model selection outcome tables for each of these main models within supplementary materials (as Tables S1 – S8).

Specific problems with the approach:
It is not clear how many models in total are actually run. What is clear is that the models haven't been selected on the basis of biological hypotheses. I suspect that a function like dredge has been used to make *every* combination of models. This is dangerous, as it can produce meaningless models.

> Authors' Response: We have embraced the suggestion of switching to a full model approach throughout, and this leaves our findings unchanged. We do agree that there are reasons to consider a 'full model approach' a more conservative means of assessing the strength of evidence for effects of our primary terms of interest than an AIC-based model selection approach.
>
> Our full models (in the original submission and now, when using the 'full model' approach) were carefully specified to test the predictions of our focal hypotheses regarding the effects of helper numbers (see the Introduction), while also allowing for the potential effects of variables that we considered *a priori* to be potential causes of variation in the focal response term. While it is no longer relevant to our current 'full model' approach, we note that the use of the 'all subsets' approach to model comparisons in our original submission did not leave us testing biologically

implausible models as each potential subset of predictors from the full model does reflect a biologically plausible scenario in this case.

Some of the models presented have interactions without the main effects being in the model - for example, in table 1 the third model has a clutch size x male helper number interaction, without either being included as main effects. Personally I see no plausible biological hypothesis that is being tested with this model.

Authors' Response: This was actually an error in our original Table 1; thank you for pointing it out. Whenever interactions were included in our models, the interacting terms were also always included as main effects (as you highlight should be the case). The interaction in Table 1 should have been stated as '*Egg position x Number male helpers*' not '*Clutch size x Number male helpers*' (note that Table 1 has now changed compared to the original submission; it now contains no interactions as, following your suggestion, non-significant interactions were dropped prior to the full model reporting for the remaining terms).

A large number of interactions are also included, but as far as I can see, no biological justification is given for the inclusion of *any* of these interactions in the manuscript. This is problematic - there is a huge amount of unnecessary testing that is going on here.

Authors' Response: We agree that only biological justifiable interactions should be considered and that we should have justified our choices more clearly. We don't cover all of the biologically plausible interactions tested in detail in the Introduction as we didn't want to distract the reader from the main effects of interest. We have addressed this concern by including *a priori* justification of these interactions within the Statistical Methods (e.g., L523-524 and L551-552; which provides full coverage of all interactions tested).

This method (model selection in general) may also bias estimates, and even produce spurious relationships, when predictors are correlated (see for example Freckleton 2011 BEAS). This can be seen, for example, in tables S1 and S2, where the female helpers estimate changes considerably (by over 10%) with the inclusion of male helpers, and vice versa - the male helper effect doubles when female helpers is not included.

Authors' Response: Switching to the suggested 'full model approach' has now addressed this concern. Our biological conclusions remain unaltered.

The method is also used in a way I can't really understand - model selection is used, a set of models is retained. This is based on delta AIC<6 and a nesting rule, both of which I am personally completely unfamiliar with. Without further investigation I am willing to accept there is some basis to this. However, the 'top-performing' models are then presented, based on delta AIC<2 (Tables 1 and 2), although this decision is justified or even mentioned in the methods.

Authors' Response: We apologise for the lack of the clarity. Switching to the suggested 'full model approach' has now addressed this concern. With regard to the AIC-based model selection tables that we now present in supplementary materials

(to complement the full model approach in the main paper), we have amended the table legends to address this comment..

Then the results from the 'top' model are presented in the text, and inferences based on this. Again this is not justified, and seems misleading. For example, the effect of both delta female helpers and mean female helpers on provisioning rate is not present in all the retained models (delta AIC <6), or even in all of the most highly ranked ones (delta AIC<2) - yet if presented as a major result (it is in the abstract for example), based on the presence of these variables in the top model. There needs to be a consistency in the approach being applied.

Authors' Response: Switching to the suggested 'full model approach' has now addressed this concern and clarified how we report our findings, which remain unchanged. Additionally, revising our analysis also revealed a typo in a script, whose correction led to a slight increase in sample size from 108 to 124 measures of maternal provisioning rates (due to a small coding error when applying the filtering criteria that we described in methods, we were erroneously filtering out 16 observations). As a consequence, we have updated all analysis with the corrected dataset (our conclusions remain unaltered) and have thoroughly double checked all our scripts.

2. I think there is a general issue with power here, with there not being sufficient data to well estimate the within and between mother effects that the paper is selling itself on. I think this is reflected in the model selection approach - see comment above - and is also clear in the large confidence intervals of the within and between mother effects for egg size and provisioning.

Authors' Response: We agree that teasing apart within and between subject effects is demanding with regard to data structure and volume (hence the rarity of studies explicitly investigating plastic responses of this type). However, our analyses do provide statistical support for the within-subject effects hypothesised. This was the case using our original statistical approach and remains so using the suggested full model approach that we now present. As such, we don't see an immediate reason for concern that a lack of statistical power could explain our findings.

3. Personally I think more could be done here to weedle out the interesting effects. Currently a model of egg size is run and a separate model of post natal provisioning. However, at no point is it shown that there is any correlation between the two, within or between mothers. To me the first step here would be to identify whether, within mothers, there is a negative correlation between egg investment and provisioning, and then whether this effect is mediated by helper number.

Authors' Response: Thanks for this suggestion. In the absence of a dedicated long-term field experiment to manipulate pre-natal investment, identifying such a trade-off between pre- and post-natal investment in our system is likely to be very difficult, if not impossible.

As the abiotic environmental conditions that would be expected to affect maternal condition and associated investment vary markedly between breeding attempts in

this system (as rainfall is highly variable in this semi-desert environment; Capilla-Lasheras *et al*. 2021 *Science Advances*), but are likely to be positively correlated between the pre- and post-natal investment stages within a given breeding attempt, we would expect natural variation in maternal pre- and post-natal investment to be positively correlated (both within- and among-mothers) even if mothers did experience a trade-off between pre- and post-natal investment.

While the existence of such a trade-off between pre- and post-natal investment is one potential mechanism that could explain our findings, such a trade-off is not required to explain our findings; the alternative mechanisms outlined in our discussion could also account for the observed patterns (whether acting instead of, or in concert with, such a trade-off). As such, while we would also be keen to know whether such a trade-off is at play, our interpretation already allows for the possibility that it may or may not be the major driving mechanism here.

4. Generally it is better to do these within and between models as bivariate models, from which the regression coefficients can be derived, see for example Phillimore et al. 2010 PNAS. This method was used recently by Fortuna et al 2021 JAE in the same context as used here. The slopes can be biased if there are few repeats per female (which doesn't seem to be the case here) or the repeatability in helper number is low (which I suspect is the case, because the mean helper number will be poorly estimated. The extent of the bias can be calculated (Ludtke et al 2008 Psychological Methods), and can be large is the repeatability of helper number if low (see Figure 1). A bivariate approach is preferable, as the error is incorporated into the analysis.

Authors' Response: Thanks for this suggestion. We acknowledge the merit of this complementary approach in some cases, but we do not agree that the suggested bivariate method is always preferable.

First, simulation studies that have compared the performance of the suggested bivariate approach and the within-subject centering approach that we employ show that the latter provides robust estimates, particularly of the *within*-subject slope which is the term of most interest in our analysis (Westneat *et al*. 2020; as a significant within-subject slope is indicative of plasticity). Westneat *et al*., 2020 shows that low predictor repeatability (i.e., of helper number in our case), can bias the *among*-subject slope, but not the *within*-subject slope that again is the main focus of our analysis. Moreover, the high number of within-subject egg and helper number observations that our dataset contains also suggests that any bias in the within-subject slope is likely to be minimal (Westneat *et al*. 2020).

Second, including (unpartitioned) female or male helper number as a response variable in the suggested bivariate model is more demanding of the dataset and carries additional statistical complications. For example, the variables female and male helper number are not normally distributed (nor are their model residuals in a Gaussian mixed model) which would cast doubt on the utility of the estimates of a bivariate model that assumed their normality. Log transforming these variables would not completely solve the problem and would modify the scale of the data, leaving it difficult to draw biological inferences.

Overall, as the modelling approach that we employ is able to calculate within-subject slopes with minimal bias (Westneat *et al*. 2020), avoids unnecessary statistical complexities and provides results whose biological interpretation is straightforward, we have continued to use this approach in the revised version of our manuscript.

5. No data is presented on chick mass at later stages. I suspect in a species like this with extensive post-natal care, the effect of egg size wears off pretty quick and the effect of post-natal provisioning becomes dominant (see for example Thomson et al 2017 evolution for a nice demonstration of the strength of prenatal and postnatal effects over ontogeny in blue tits - the prenatal effects have previously been linked to egg size (Hadfield et al 2013 evolution)). So I think it would be interesting to know the impact of increased egg size compared to decreased maternal provisioning on chick size at fledging for example (when a lot of presumably size related mortality occurs). From an evolutionary perspective, the costs and benefits of these strategies should balance out.

Authors' Response: We agree that effects of the pre-natal environment are likely to become less important the later one looks in an individual's life due to the progressive accrual of other environmental influences. However, as egg size predicts hatchling mass (shown here) and hatchling mass predicts survival to fledging (Table S3b in Capilla-Lasheras *et al.* 2021) in this system, it still seems likely that being born from a smaller egg entails a fitness cost on average for offspring. It isn't clear to us that assessments of the body mass of offspring at later life stages would shed further light on this, as such an analysis could only assess effects on the subset of offspring that survived to that life stage (thereby missing the fitness cost already paid via the lighter hatchlings' reduced survival to fledging), and as formative effects of pre-natal investment could also manifest via effects on other fitness-related traits. We agree with the latter point, that it would be interesting to be able to assess whether a 'unit' of maternal investment does indeed have greater fitness consequences for offspring if spent pre-natally than post-natally (as one might expect if maternal front-loading was adaptative), but we are not in a position to investigate this as we lack the ability to unite pre- and post-natal investment in a common currency of maternal investment.

Specific Comments:
l 150 - why not include this in the main paper? More generally, there seems to be a lot of analysis in the supplement, and actually not too much in the main paper.

Authors' Response: Thanks for this suggestion. We have now included the clutch size analysis within the main text as requested (L238-251).

l 182 Please present repeatability of egg size.

Authors' Response: As suggested, we have included this estimation (see L189-191).

Egg volume and egg mass
First, I am not sure why this is presented as a regression rather than a correlation. I then don't really understand why the within mother effects are presented? Why would egg

volume and egg mass be predicted to covary differently between and within mothers? Surely the question here is just are egg volume and egg mass highly correlated?

> Authors' Response: We focus here on the within-mother relationships in order to highlight the extent and biological significance of variation in egg volume *within* mothers (before we then investigate the causes of this within-mother variation).
>
> It is worth demonstrating that these relationships exist *within* mothers because egg volume and hatchling mass could be highly correlated *among* mothers (e.g., if some mothers always produce larger eggs and hatch heavier chicks), without being evidently correlated within mothers (e.g., due to a lack of within-mother variation in egg volume). We apply a regression method here simply because there is a clear directionality to the associations that we are testing.

l 185 ' ΔAIC = 878.5'
What does this refer to? Is this the difference in AIC between two nested models, with and without the effect of interest? Personally, I think it would be much more intuitive to include a p-value or likelihood ratio. Most readers will not have a clear intuition as to what ΔAIC = 878.5 represents, whereas a p-value would be more helpful. As stated above they are essentially equivalent, but people have a better intuition about p-values.

> Authors' Response: This point has now been addressed by employing the requested p-value driven full model approach throughout.

l 187-192 'These relationships also hold within mothers,'
It is important to test the difference in between and within mother slopes to determine whether they are different. You can do this by running a model with raw egg size and delta egg size (see Van de Pol and Wright paper). If the effect is causal then you expect them to be the same. For example, for the effect of egg size on hatching mass, 0.468 ± 0.048 and 0.282 ± 0.048 are very different slopes, and the between mother slope is not reported. Presumably the between mother slope is then much larger than the within mother slope, indicating that egg size itself is unlikely the main driver of chick mass, rather something correlated with egg size at the maternal level.

> Authors' Response: Our text here was highlighting that there is significant effect of within-mother variation in egg size on hatchling mass, allowing us to infer the existence of a plastic maternal reaction norm between the two variables. We could also have confirmed (as requested, and as we do below) that there was no significant difference between the effect sizes for the within- and among-mother components of variation in egg size; a scenario that would be consistent with the population level effect arising *solely* from the already-reported within-mother effect. However, (i) the lack of such a significant difference could also arise because of low power, and (ii) if there *was* a significant difference between the within- and among-mother effects this would not tell us that the within-mother effect was unimportant, it would just tell us that differences among mothers *other* than those arising from the within-mother effect *also* contribute significantly to the observed population level relationship. As determining whether the latter is the case isn't a priority for these relationships between egg volume and both egg mass and hatchling mass (our interest is focussed on the reported within-mother effects),

we'd prefer to avoid complicating this introductory results passage by including these additional tests (as doing so would also require including the above explanation of what the outcome of these tests does and does not tell us).

In case of interest though, here are the results of the requested tests (having also included maternal ID in the models as requested below and removed the standardisation of the predictors as requested by R1): the effect size estimates for the within- and among-mother components of egg volume did not significantly differ in the model of egg mass ($\chi^2_1 = 0.01$, $p = 0.947$) or in the model of hatchling mass ($\chi^2_1 = 0.77$, $p = 0.380$).

We have, however, now used this within- vs among- effect size comparison test to provide more detailed information on the key female helper number effects on egg volume and provisioning (as requested below), as it is a higher priority to know for those relationships whether among-mother differences *also* contribute to the population-level results, over and above the reported within-mother effects (see below).

l 186-192 These analyses also need to be run in mixed models - the text refers to 'LM', so presumably maternal ID is not included, but needs to be. More generally, there analyses are not described in the methods, and β standardized is not defined anywhere.

Authors' Response: We agree and have now added a new methods section to describe these analyses and have run them as suggested using mixed models, including maternal ID as a random intercept (see L506-514 for details on methods).

l 194 'heavier hatchlings in this species are more likely to survive to fledging [33].'
It would be worth stating explicitly where in the other paper this result is reported (table/figure), as it is not a main finding of the paper. It seems like it comes from Table S3. If so this seems a somewhat misleading statement - the effect of egg size on survival is tested, and is 'not retained' in the model, although effect size and standard error are not reported. My point here is that if egg size has such a strong effect on hatchling size, and hatchling size has a meaningful effect on survival, I would expect to see a correlation between egg size and survival. Otherwise it suggests that somewhere along this path something isn't causal.

Authors' Response: Thank you for this comment. As requested we have now included a reference to the Table in the reference (L201 and L292) that contains the result (it was indeed Table S3).

It is true that we cannot currently directly link egg volume to nestling survival, which is why we never claim within the manuscript that we can. We draw our inference explicitly from the two component relationships that we do know exist (e.g., "Laying larger eggs could therefore have fitness consequences for mothers and the resulting offspring, as larger eggs yield heavier hatchlings (Figure 1c) and heavier hatchlings in this species are more likely to survive to fledging [Table S3 in 35]."; L198-201). As both relationships are correlations it seems unnecessary to further qualify this statement by highlighting that these relationships might not

be causal (particularly as we have not claimed that they are), but if doing so was preferable we could do that too.

It is conceivable that the lack of an evident direct relationship all the way from egg volume to nestling survival to fledging in the analysis reported in Table S3 in the cited paper reflects the *lack* of causal link between the two, despite the two component relationships being evident and the general expectation of such a link (e.g., Krist 2011 Biological Reviews). However, a simpler explanation for the lack of an evident direct link is that, when three traits A, B and C are in a causal chain, it will typically be more difficult to detect a direct relationship between A and C than the two component relationships (i.e., between A and B and between B and C) simply because the direct analysis has to deal with the residual variance in *both* of the component relationships (i.e., in this case other sources of variation in both hatchling mass (B) and nestling survival to fledging (C) in natural populations, such as weather conditions, social effects, etc). Moreover, the Table S3 analysis in Capilla-Lasheras *et al.* that uses egg volume data was not set up to test this relationship; it was set up to investigate whether helper effects on nestling survival aggregated to the brood level (the focus of that paper) could have been shrouded by not controlling for the brood's mean egg volume at laying in the analysis. As such the analysis lacks the power that would stem from of a one-to-one concordance between individual eggs and their downstream fates. As such, we feel that it is fair to retain the phrasing as we have it, in which we don't claim to have demonstrated a direct (or causal) link between egg volume and offspring fitness, but rather state that one could exist given the two component relationships detected.

l 202 'individual mothers laid larger eggs when they had more female helpers'
Is this different from l 197?

Authors' Response: in L197, we state the general result before partitioning among and within-mother variation in helper number, while in L202 we were specifically referring to, and highlighting, individual (i.e., plastic) adjustments in egg volume to the number of female helpers. We have clarified this point (L205-221).

l 205 I don't understand why there is model selection on the within and between analysis. Its not clear to me why its helpful to remove either of the variables. The difference between the two slopes also needs to be tested. The variance components from these models also need to be presented somewhere.

Authors' Response: We agree that model selection on the within- and among-mother components is not required. Switching to the suggested 'full model approach' (both before and after partitioning the helper number terms) has now addressed this concern. When fitting the partitioned terms, the full model approach confirms a significant positive effect of within-mother variation in female helper number on egg volume (Table 2). As requested, we have now also included the requested tests, which confirm that the effect sizes for the effects of the within- and among-mother components of female helper number on egg volume do not significantly differ (L214-221); consistent with expectation if the population-level positive effect of female helper number on egg volume (Table 1)

arises solely from a (causal) maternal plastic response to female helper number. We also included a brief explanation of what this result tells us, as it would otherwise be difficult to understand why the result has been included (L214-221). We also now report the variance components as suggested (e.g., legends in Table 1-4).

l 222-223 These are estimated with considerable error, and the confidence intervals would clearly overlap.

Authors' Response: Yes indeed, we agree – we have made changes to clarify the relative strength of evidence for effects of female and male helper number (e.g., L227-231 and L274-278).

First, we highlight that female helper number has a significant effect on egg volume while male helper number does not (both at the population level Table 1; and when isolating the effects of within-mother variation in these terms Table 2). This clearer signal of a role for female helper number than male helper number is consistent with the maternal response in egg volume being a response to available help, as female helpers contribute twice as much as males (and, accordingly, only female helpers have a detectable additive effect on the overall rates at which nestlings are fed; Capilla-Lasheras *et al*. 2021). Second, we note too that, accordingly, our point estimate for the effect size for within-mother variation in female helper number (0.019 ± 0.009 cm^3 / female helper) is also larger than (roughly twice) that for within-mother variation in male helper number (0.008 ± 0.010 cm^3 / male helper), but now qualify this by explicitly noting that formal tests indicate that these effect size estimates do not differ significantly (L227-231). We now take this same approach for the provisioning rate analysis too, reporting the results of testing for a difference in the effects size for female and male helpers (L274-278).

l 238-239 These estimates also have a huge amount of error associated with them, and would have 95% CIs that overlap 0. The 'best supported model' is presented, however, not all the retained models contain there parameters.

Authors' Response: Switching to the suggested full model approach has addressed this issue, by confirming a significant negative effect of within-mother variation in female helper number on maternal provisioning rate (indicative of maternal plasticity). We have also now included formal tests which confirm that the effect sizes for the effects of the within- and among-mother variation in female helper number on maternal provisioning rate do not significantly differ (lines 267-269); consistent with expectation if the significant population-level negative effect of female helper number on maternal provisioning rate (Table 3) arises solely from a (causal) plastic maternal response to female helper number.

I would suggest log transforming provisioning rate before analysis, and also would suggest not correcting for observation period in the response variable, but by including an offset of log observation period.

Authors' Response: We have double checked the assumptions of our Gaussian models of provisioning rates, which seem to be fine (i.e., residuals are normally distributed and there is no evidence for heteroscedasticity). We would therefore prefer to keep this approach.

All tables - standard error or confidence intervals need to be presented with parameter estimates.

Authors' Response: Agreed - we have now done this throughout (e.g., Table 1-4, Table S1-S8).

Plots - egg volume is not presented on the same scale it is analysed in, which makes it difficult to interpret these compared to the reported estimates in the text and tables. I would suggest using the same scale in both.

Authors' Response: Agreed - we have now done this. All text, tables and figures use cm^3 as the units for egg volume. Additionally, we have clarified the units in which every predictor was included in the analysis in methods and table legends.

Thank you again for such detailed feedback on the manuscript.
