# Supplementary material for: Mothers in a cooperatively breeding bird increase investment per offspring at the pre-natal stage when they will have more help with post-natal care
Source: PLoS Biol. 2023 Nov 9;21(11):e3002356. doi: 10.1371/journal.pbio.3002356 (PMC10635431; doi:10.1371/journal.pbio.3002356)
Supplement: Attachment — Submitted filename: Response to Reviewers - PBIOLOGY-D-22-02779R1.pdf [file pbio.3002356.s042.pdf]

Point-by-point response to reviewers' comments - PBIOLOGY-D-22-02779R1

Reviewer #1:

This version of the manuscript has been rendered much more intelligible with the abandonment (or relegation to the SI) of the convoluted AIC methods used in the earlier version. Hence it is now possible to address what has actually been done. The authors contend that females in cooperatively breeding birds can vary egg provisioning in two very different ways in responses to changes in helper number. In one scenario, females can improve their own survival by reducing the investment in eggs in anticipation that the resultant small young will not suffer because larger groups have a dependably greater corporate feeding rate. The alternative hypothesis, which the authors use to explain data from arid-zone sparrow-weavers, is that because the adult females can anticipate greater care from helpers but cannot gain assistance from helpers during the incubation phase, they invest more heavily in eggs and hence can lessen their own contribution to offspring provisioning. Indeed, one of the strange things about this system is the massive variation in egg size - the largest eggs are 1.61 cm3 (57%) larger than the smallest eggs. I presume this variation partly motivated the study, as such variation in a low-fecundity small passerine seems quite unusual.

> Authors' Response:
> Thank you for your review. Please, see a point-by-point response to your observations below.

Much of the (presumably) between brood effects are driven in understandable ways by rainfall (0.31cm3 per 100m of rainfall). Within brood effects are driven mainly by egg position (-0.04cm3) from the first to the second egg, though the Methods for this are not given in the paper (see below).

> Authors' Response:
> We have added a sentence in the methods to clarify this point (please, see L494-497). Thank you for highlighting the need for this.

The data for within female change in helper number are largely confined to a maximum increase or decrease of 3 from the average produced by the female, which is predicted to lead to a change of 0.057cm3, or about 3.5% of the natural variation in egg size. A change in one helper is therefore about 1% of the natural variation (as can be understood by looking at the cloud of points in Fig. 1b). I am afraid I still find the likely biological effect of this uncompelling.

> Authors' Response:
> Thank you for this comment. We do appreciate the need for caution regarding biological significance and have made adjustments to the discussion to more directly engage with this issue (see details below).
>
> While the effect size for the maternal plastic response in egg volume to helper number is modest (as we acknowledge; L283-284 in the latest version), we nevertheless consider it an important finding for three main reasons. First, this is the first evidence of the hypothesised plastic maternal variation in egg size according to helper numbers in a cooperative bird. Second, the observed maternal plastic response actually runs counter to the population-level relationships generally reported to date between helper number and egg size (Dixit et al., 2017; Russell et al., 2007). Third, while the effect size for our maternal plastic response is modest, it actually aligns well with the effect sizes for the population-level relationships between helper number and egg size that are already the focus of research in this field. For example, Figure 2a in Russell et al., (2007), the landmark paper that stimulated research in this area, suggests that the effect size in that study for the contrast in egg volume between mothers with and mothers without

helpers was around -0.067 cm^3 of egg volume (-5.3% of mean egg volume), which is broadly comparable in both absolute and proportional magnitude to the within-mother (i.e., plastic) effect size that we report here; for six female helpers (the natural range in female helper number in our analysis) the change in egg volume would be 6 x +0.019 cm^3 (see Table 2) = +0.114 cm^3 of egg volume (+3.3% of mean egg volume). As such, while it is reasonable to consider the likely biological significance of these effect sizes (which we now do in a more nuanced way in the discussion; see below), the magnitude of the effect sizes in our study needn't be considered unusual, beyond being the first that demonstrably reflect the maternal plastic response. We have now included a brief statement in the discussion (L304-309) to help place our effect size in context by making reference to this qualitative comparison (itself now included in supplementary materials).

The other panels in that same figure of Russell et al., (2007) also reveal that that same effect size for egg volume was linked to significant effects on yolk wet and dry mass and lipid content, rendering it plausible that similar effects on consequential egg constituents are occurring in our study too. Indeed, the modest statistical effect size for egg volume in these studies could underestimate the biological effect of the maternal response in egg investment on offspring fitness, as any associated increases in nutrient provision could have stronger effects on offspring fitness than they do on egg volume per se (something we now note in L449-452).

Nevertheless, adaptive explanations for our results do hinge upon their biological significance, so we appreciate the importance of engaging with this issue in a more nuanced way when considering such explanations. In addition to the changes highlighted above, we also now provide (i) a more nuanced treatment of the potential routes through which egg volume could impact offspring fitness in this system (L413-433) and (ii) highlight that while the field is focussed on adaptive explanations for plasticity, we should acknowledge too the wider possibility that the net fitness consequences of the observed plastic response are not sufficiently strong across contexts for it to have been optimised by selection, such that adaptive explanations may not apply (L408-411).

The appropriate data are not really presented in this manuscript, but the authors suggest that heavier new-hatched chicks (unsurprisingly from heavier eggs) are more likely to thrive, yet the effects of helper number seem to be more profound for post-natal provisioning rates. However, this difference is difficult to interpret in the absence of corporate provisioning rates. What is clear is that the mother responds far more dramatically to variation in brood size than she does to the work force available. However, we don't know if similar responses occur in all group members.

Authors' Response:
Thank you for this comment. We appreciate the reason for the interest in the nature of the helper effects on the overall rate at which nestlings are fed in this species; as our work in this paper has highlighted that helpers may indirectly allow mothers to increase egg volume, the *overall* effects of helpers on offspring performance in this species will arise via a combination of these two mechanisms. As such, at the point in the introduction in which we highlight that female helper numbers are known to have a causal positive effect on the overall rates at which broods are feed, we now highlight not only the relevant paper in which we show this (Capilla-Lasheras et al., 2021) but direct the reader to the relevant figure for inspecting the effect sizes in case of interest (L151-152). We have also now added text to the discussion to attend to this issue of the likely relative importance of the pre-natal and post-natal components of any overall helper effect to the extent that we can (L444-449).

A further problem is that although the authors present the among female variation as a solution to the pervasive problem of whether it is possible to attribute the direction of causation in correlations between performance and helper number in cooperatively breeding societies. This method has been extensively discussed by Dickinson & Hatchwell (2004) Chapter 3 in Ecology and Evolution of Cooperatively Breeding in Birds, and I do not think that the difficulties they discuss are addressed by this method. In the present case, there is no help given to the reader concerning why the number of helpers fluctuate from clutch to clutch. The expectations are quite different if either: i) as is plausible in a desert environment, the reduction in helpers is associated with a collapse in population number, or ii. if amelioration in conditions through increased rainfall and the absence of heatwaves is associated with splitting of groups as more birds are able to attempt independent reproduction.

Authors' Response:
We agree that there is scope to add some additional caution regarding causality here. We have therefore adjusted the discussion to more fully unpack the extent to which our analyses address other potential confounds including those outlined above (see L300-304 and L318-325).

As the analysis of the effects of female helper number is within-mother (and hence within-territory, as mothers only ever breed on one territory) this approach should minimise problems arising from among-mother and among-territory confounds of helper number. However, the reviewer is correct in that there remains the potential for within-mother variation in female helper numbers to be correlated with within-mother temporal variation in abiotic factors (e.g., rainfall or temperature) via mechanisms such as those that the reviewer highlights above. However, this is unlikely to be an issue here as (i) our analyses simultaneously control for effects of rainfall and temperature (which we allow to take many forms through the sliding window approach) alongside the helper number terms, and (ii) neither rainfall nor temperature are correlated with female helper numbers (e.g., for the 490 eggs in the egg volume analysis, the correlation between female helper number and the rainfall predictor in that analysis = 0.030, $t = 0.65$, $df = 488$, $p = 0.514$, and the correlation between female helper number and the heat waves predictor in that analysis = 0.050, $t = 1.11$, $df = 488$, $p = 0.269$). This lack of a correlation between female helper numbers and abiotic factors is consistent with the natural history of the birds, as their group compositions are not dynamic over short timescales. Instead, young birds typically remain resident subordinates within their natal groups until they disperse to long-term residency within another group or die (Harrison et al., 2013).

The authors correctly point out that conclusions can be drawn from a greater change in numbers of female helpers than an equivalent change in the number of male helpers. However, it is not really explained why this is the case. Indeed, since Cockburn (1198, ARES) drew attention to the paradox that male-biased help is more common in cooperatively breeding birds, but female helpers more often seem to increase reproductive success, there has been no real progress in understanding of why this is the case.

Authors' Response:
Thanks for this comment. We too are intrigued by this and are currently investigating potential evolutionary drivers of this sex difference in helper effect.

In sparrow weaver groups, female helpers likely have a greater effect than male helpers on the total rate at which offspring are fed (Capilla-Lasheras et al., 2021) and maternal investment at both the pre- and post-natal stages (this paper), simply because female helpers feed offspring at twice the rate of male helpers (Capilla-Lasheras et al., 2021). We explain this in the introduction (L148-156) and discussion (L309-316).

While the evolutionary origin of this sex difference in helper effort is beyond the scope of this manuscript, we can share here some insights from our work so far. Our findings to date suggest that this sex difference in helper effort arises at least in part from a sex difference in dispersal activities that trade-off against helping. While both sexes typically disperse to breed (Harrison et al., 2014), male helpers disperse further (Harrison et al., 2014) and sooner than female helpers. Accordingly, a large-scale radio tracking study (unpublished) has shown that male helpers engage in higher rates of extra-territorial prospecting for dispersal opportunities (subordinate males do not secure extra-group paternity) than female helpers do and that investment in these prospecting forays appears to trade off against helping. Thus, it appears that male helpers, being more dispersive, invest more in prospecting forays and less in helping than female helpers do.

Technical comments

Methods

436-441 - I could not find methods for converting volume to mass - was this done with Hoyt's formula. If so, it should be stated.

Authors' Response:
Thanks for highlighting the lack of detail here. We have added a clarification in L495-497.

Results
189-191. How much of maternal repeatability is associated with between versus within brood effects (see lines 184-196).

Authors' Response:
The quantification of maternal repeatability that we present in the main text already accounts for variation among broods, which explained 4.7% of the total variation in egg volume (with maternal repeatability accounting for 69.3 % of this variation [L199]). Additional analysis revealed that out of the 69.3% of variation in egg volume explained by mother ID, 20.8% is explained by among clutch variation while the remaining is explained by within clutch variation.

191-194. How were individual eggs marked and then connected to individual chicks, as is done on Fig 3. I couldn't find this anywhere in the paper, though 'egg position' is used as an important variable in the models, and is highly significant in Table 1. It is quite unusual to have substantial hatching asynchrony in birds with a clutch size of 2. If it assumed that the first hatched chick is associated with the first laid egg the pattern may exist through the assumption rather than any biological effect.

Authors' Response:
Thank you for highlighting this. We have added a clarification in L500-502 that eggs and hatchlings were linked by direct observations; the process involved no biological assumptions. Briefly, daily checks for new eggs were conducted through the laying period, and any new eggs were individually marked upon discovery (using a non-toxic marker). As only one egg is laid per day, laying order can be known via daily checks. Daily checks during the hatching period then also allowed us to link individual hatchlings to individual eggs whenever only one of the two eggs had hatched at the time that we checked the nest (as we could see which marked egg was still intact).

200-201. This is an important point, and it is strange that it demonstrated by reference to Supplementary Information in another paper.

Authors' Response:
We agree that this is an important point. We now unpack this point in a more nuanced way later in the manuscript (L413-433).

238-251. It seems strange that there is no effect of clutch size on egg volume, but there is a very strong effect of position in the clutch.

Authors' Response:
Thanks for this comment. Including egg position and clutch size in the analysis allow us to investigate predictors of egg volume variation within clutches (i.e., egg position) and among clutches (i.e., clutch size). The results highlight that clutch size does not have an overall impact on egg volume, while within clutch variation in egg volume is explained by egg position (e.g., 1st laid eggs are larger than 2nd laid eggs, while 1st laid eggs of clutches of different sizes are of similar volume).

255+. The difference between male and female helpers raises the question of whether dominant males and other helpers also load-lighten, but this cannot be assessed.

Authors' Response:
Indeed, we are also interested in the potential for, and consequences of, paternal and helper responses to the available help. While these lie beyond the scope of the present study, we do expect to address these questions in future work.

Thank you for your review and comments.

Reviewer #3:
[identifies himself as Joel Pick]

I think this version of the manuscript is much improved from the last one. Many of my previous points have been addressed by the authors adoption of a different analytical approach. It is nice to see that this doesn't substantially affect the results, and some interesting patterns remain.

There are still a few points which I do not feel have been addressed sufficiently in the response and revised manuscript and remain unresolved. I don't think that they will alter the main message of the paper (the within mother effects of helper number on egg size and provisioning rate), but I think it is worth using the appropriate statistical models and trying to present unbiased results where possible.

> Authors' Response:
> Thank you very much for your latest review. We appreciate the time spent providing suggestions, which we feel have again helped to strengthen the manuscript. We have carried out all of the suggested additional analysis and our initial conclusions remain robust to their findings. We have included these additional findings in the main text of the paper, the supplementary materials or this response letter. We provide a brief summary of our responses to the main points below and follow up with a detailed point-by-point response to your numbered comments below.

The main comment relate to (addressed in more detail below)
- explicit testing for a within mother relationship between egg size and provisioning rate (comment 1)

> Authors' Response:
> We have done this analysis as requested, included its results in the supplementary materials (Supplementary Material H) and added a new paragraph to the discussion to integrate these findings into our discussion of the relevant adaptive explanation for our findings (L396-411).

- use of multivariate models to reduce bias in estimates (comment 2)

> Authors' Response:
> As suggested, we have adjusted the results section in the main text to focus on the effects of within-mother variation in helper numbers (from which we draw our inferences regarding the evidence for maternal plasticity), and removed the comparisons of the effect sizes for these with the effect sizes for among-mother variation in helper numbers (given the potential for the latter to be biased, and as we don't draw inference from these comparisons anyway). We have also carried out the additional suggested bivariate analysis, that should avoid such potential bias in the among-mother estimates, but which also entails certain acknowledged limitations. This bivariate analysis also supports our conclusion of a within-mother plastic response; it suggests that the within- and among-mother effect sizes for female helper number are very similar to each other, consistent with expectation if the significant positive effect size for population-level variation in female helper number is indeed driven (as our existing analyses suggest) by a within-mother effect of female helper number. Full details are provided below.

- why the egg size data set is halved, based on whether data on laying order is available (comment 20)

Authors' Response:
It was a priority to conduct the analysis of egg volume with information available on egg position (laying order), to allow for plausible and potentially important interactions between egg position and the focal helper number terms (see detailed replies below; Fortuna et al., 2023), as well as to control for the significant effect of egg position. Expanding the data set to include all of the egg volume data for which egg position information was unavailable, would also introduce additional uncertainty in clutch size (another key predictor in the model), as assessing both traits accurately required daily nest checks throughout the laying period. That said, we have confirmed that expanding our analysis to this larger data set still yields a signal of the helper effect despite the potential issues. Full details are provided below.

In reply to my previous comments and the responses:

1. Testing for a relationship between egg size and provisioning rate (my previous comment 3).

In the response to my previous comment, it is argued that this wouldn't be meaningful without an experiment. But the same can be said for the relationship between maternal expenditure and helper number. This is observational data and has its limitations, but I don't see why that is reason to not fully explore the hypothesis suggested here. For example, if female helper number provides general benefits to the group, then group size could reflect maternal condition pre-laying, and so the increase in egg size could be a function of maternal condition, rather than anticipation of future care from female helper. To me this seems completely plausible, but is not an alternative explanation explored in the manuscript, and cannot be ruled out without an experiment. Therefore, I think adopting a within/between mother approach to the relationships between egg size and provisioning rate would be no less reasonable than the analyses presented in the manuscript, and directly test the prediction that a change in egg size is accompanied by a change in provisioning rate, mediated by helper number (L 172).

A nice approach to showing that any relationship between the two was mediated by helper number would be to run a bivariate model of egg size and provisioning rate, within and without helper number as a covariate for both traits, and then see how the within mother covariance changes as a result. This would be similar to adopting a hybrid trait-based/variance component approach used to identify traits mediating maternal effects in quantitative genetics (Mcadam, Garant and Wilson 2014 in Quantitative Genetics in the Wild). Additionally, by having temporal random effects (such as breeding season) in this analysis would allow the problem of a correlation driven by the environment that is raised in the response to be mitigated.

Authors' Response:
We have implemented the suggested model following McAdam et al., (2014). We report its results in supplementary materials (Supplementary Material H) and interpret the implications of them for the potential explanations for our findings in a new paragraph in the discussion (L396-411).

As suggested, we fitted a bivariate model with egg volume and maternal provisioning rate as responses. For both responses, we used the fixed effect structure that we specify in the main text and show in Table 1 (for egg volume) and Table 3 (for maternal feeding rate). The random effect structure of this bivariate model included group ID, season ID, mother ID and clutch ID variance terms for each response term as well as mother ID and clutch ID covariances. The model also estimated residual variance for egg volume.

The residual variance for maternal provisioning rate was fixed at 0 as the dataset contained only one observation per clutch ID. Therefore, the clutch ID covariance term captures covariation between egg volume and provisioning rate within mothers. We could only use data for 59 clutches from 36 mothers (108 observations of egg volume and 59 observations of maternal provisioning rate) for which we had both egg volume data and maternal feeding rates for the same clutch. As such, the power of the analysis to capture the within-mother trade-off may not be high.

Following McAdam et al., (2014) we fitted this model i) without female and male helper number as fixed effects for either response term and then ii) with female and male helper number as fixed effects for both responses. We then compared the effect of including these predictors on the size of the within-mother covariance between egg volume and provisioning rate. In both cases, the estimate of within-mother covariance between egg volume and maternal provisioning rate was close to zero and it did not change much between models. We have included these results in Supplementary Material H and refer out to them from the discussion (see new paragraph L396-411) where we also consider their implications for the potential explanations for our findings.

2. Using bivariate models to account for the known bias in within subject centering models (my previous My comment 4).

The authors are right that the main bias in these models is in the between subject slopes. However, given the that between subject slopes and the difference are reported in the paper, it seems that strange to use a method that is known to be biased for the estimation of these additional parameters. For the analysis of provisioning rate in particular, this bias is likely to be large, ad there is a median of 2 observations per mother. I think that either the between mother slopes and difference should not be reported in the main text because of the known bias (and this bias and lack of presentation explained clearly in the manuscript), or a bivariate approach used.

I would strongly recommend the use of bivariate models, and the presenting of a more complete and unbiased set of parameters. The fact that the within mother slopes appear to be stronger is interesting in of itself. As stated in the introduction, one of the reasons for using the within subject centering approach is that between mother slopes may obscure the within mother slope of interest. However, if anything, the opposite is found. If helper number had a causal effect on the response alone you would expect the same slope in the two components. This means that the mothers are either not reacting to the absolute number of helpers, or there is an additional correlation in the opposite direction between average maternal expenditure and helper number which is interesting to discuss. The difference between the two is not statistically supported with these models, but its should be noted that the bias would decrease the difference between the two slopes, and so the bivariate approach may give greater granularity over whether there is a difference. To me it seems strange not to want to explore this further.

Authors' Response:
We have followed your recommendations and implemented the suggested model for the analyses of egg volume and maternal provisioning rates, which both yield results consistent with our existing conclusions of maternal plasticity.

First, as suggested, we have revised the presentation of our existing analyses using the within-subjects centring approach by removing from the main results text both the formal comparisons between the within- and among- mother slopes and the main-text reporting of the among-mother slopes (as our inferences regarding maternal plasticity stem only from the significance of the within-mother slope). We have also acknowledged the potential for this centring approach to yield a biased estimate of the among-mother slope

and explain that for this reason we do not make or report a formal comparison between the within- and among-mother effects and only present the among-mother estimates within the full model output tables, citing your suggested literature (see L727-731). We have also highlighted in the relevant tables that report the model outputs following centring that the among-mother slope estimates should be treated with caution for this reason (Table 2 [L963-966] & Table 4 [L992-995]).

Then, we carried out the suggested bivariate analyses to confirm that they yield the same conclusions that we present in the main text. Using this method, we find that the effect sizes for within- and among-mother variation in female helper number do not differ. As the reviewer highlights above, this finding is consistent with the significant female helper number relationships at the population level being driven by a causal effect of within-mother variation in female helper number (and hence with our existing conclusions from the significance of the within-mother effects using the within-subject centring approach).

We have not included the results of the bivariate models in the manuscript as we are not primarily interested in this comparison of the within and among mother effects. However, we present the details and results of these models below and would be open to including them within the supplementary materials to complement the existing analyses if there was a strong preference for this.

Bivariate model for egg volume, including female helper number as the second response term:
We followed the model specification described in the main text for the egg volume model, but including female helper number as a second response term. We included group ID, season ID and mother ID variance and covariance terms for each response term. The model also estimated residual variance and covariance. Both responses were mean-centred and scaled and assumed to follow Gaussian distributions (thanks for the reference showing that Gaussian mixed models deal well with several data types). This model was fitted in R using the MCMCglmm package (v2.34; (Hadfield, 2010)), using an inverse Gamma as prior distribution for random and residual (co)variances (V = 1, nu = 1.002), 50000 MCMC iterations, with 1000 as initial burn-in and sampling every 10 iterations. Effective sample sizes for all model terms were always higher than 4000 and MCMC traces were visually inspected to confirm convergence. We calculated the correlation between egg volume and female helper number within and among mothers by dividing the corresponding covariance between egg volume and female helper number by the product of the square-root of their variances. We found that both the within- and among-mother correlations between female helper number and egg volume were positive (correlation within mothers [95% CrI] = 0.074 [-0.017, 0.170]; correlation among mothers [95% CrI] = 0.099 [-0.257, 0.451]) and that their difference was close to zero (estimated difference between within and among mother correlations [95% CrI] = -0.025 [-0.409, 0.338]), consistent with expectation if the significant population level association arose from a causal effect of within-mother variation in female helper number.

Bivariate model for maternal provisioning rate, including female helper number as the second response term:
We followed the model specification described in the main text for the maternal provisioning model, but including female helper number as a second response term. We included group ID, season ID and mother ID variance and covariance terms for each response term. The model also estimated residual variance and covariance. Both responses were mean-centred and scaled and assumed to follow Gaussian distributions. This model was fitted in R using the MCMCglmm package (v2.34; (Hadfield, 2010)), using an inverse Gamma as prior distribution for random and residual (co)variances (V

= 1, nu = 1.002), 50000 MCMC iterations, with 1000 as initial burn-in and sampling every 10 iterations. Effective sample sizes for all model terms were always higher than 4000 and MCMC traces were visually inspected to confirm convergence. We calculated the correlation between maternal provisioning rate and female helper number within and among mothers by dividing the corresponding covariance between maternal provisioning rate and female helper number by the product of the square-root of their variances. We found that both within- and among-mother correlations between female helper number and maternal provisioning rate were negative (correlation within mothers [95% CrI] = -0.156 [-0.400, 0.067]; correlation among mothers [95% CrI] = -0.181 [-0.664, 0.311]) and their difference was close to zero (estimated difference between within and among mother correlations [95% CrI] = 0.027 [-0.558, 0.593]), consistent with expectation if the significant population level association arose from a causal effect of within-mother variation in female helper number.

In terms of the additional complexity of the bivariate model - I disagree. First, linear mixed models are amazingly robust to non-normality of the data (see for example Schielzeth et al 2020 MEE). Second I believe that the inferences would be *more* meaningful on the log scale. On re-reading the manuscript (before reading the responses) it occurred to me that helper number should anyway be log transformed. It is a count, and so inferences make more sense on a multiplicative scale. Not log transforming will also weaken any relationship between helper number and the response, if they are linear on that scale. The same also holds for provisioning rate (which I previously commented upon). The difficulty with log transformation would come with the presence of 0s - this can be handled by adding 0.5 (Yamamura 1999 Population Ecology), but is not very satisfactory. The data could also be treated as Poisson. It's worth noting that because the within subject centering is done with two variables, a bivariate model wouldn't strictly be appropriate. As shown in Froy et al 2019 Science (p6 of supplements) you can extend this bivariate approach in Phillimore et al 2010 PNAS to many variables.

Authors' Response:
We have followed your suggestions above and implemented bivariate models to inspect within and among mother correlations between helper numbers and both egg volume and maternal provisioning rate (see above). Thank you for highlighting Schielzeth et al., (2020); we found it a useful reference. Following their findings, we used Gaussian distributions to model maternal provisioning rates in the bivariate models presented above. As requested below, we have also run additional Poisson models of maternal provisioning rate (following the suggestion to model the underlying process), and these yielded identical results too. It is not clear to us why helper numbers should be log transformed as a predictor, particularly from a biological point of view, as helper effects are often linear. We have therefore kept this predictor on a linear scale. Full details can be found in our detailed response to the request to model maternal provisioning data using a Poisson model below.

The response also states that a 'bivariate model is more demanding of the dataset'. My reading of this is less likely to give a significant p-value - personally I would advocate being more worried about biased estimates and less about significance thresholds.

Authors' Response:
Thank you for this suggestion and observation, which we agree with and have tried to take on board. Please, see the results of the bivariate models above.

As a slight aside, the response and revised manuscript cites Westneat et al 2020 (citation 59) as a justification for the use of these models. A more appropriate reference is Ludtke et al 2008 Psychological Methods, as a) they derive the biases in these models and b) Westnest et al specifically addresses the scenario where there is between subject variation in the

response to the environment (i.e. random slopes), and addresses how models with and without mean centering (but specifying random slopes) cope with matching or mismatching data generating processes. Westnest et al do compare a bivariate model to these random slope models, as bivariate models have been suggested as a solution (Phillimore et al. 2010 PNAS) to the known bias in within subject centering models shown in Ludtke et al 2008, but never use the model without random slopes that is used in this manuscript.

Authors' Response:
Thank you for this further suggestion, which we have followed (we now cite Ludtke et al., (2008) in L729, Table 2 and Table 4).

3. Egg volume and egg mass
I am still unsure why there an analysis of within and between mother effects of egg volume on egg mass. My understanding of this is to show that egg volume is a good proxy for egg mass, which is a better measure of expenditure. In the response to this comment in my previous review the authors simply justify why this has been done for chick mass. The reason for analysis with check mass is clear (indeed I have published a paper promoting this approach for this question), but this doesn't answer what relevance this has to egg volume and egg mass. I think a simple correlation would suffice here.

Authors' Response:
We have fully followed this suggestion. We now only show the population-level relationship between egg volume and egg mass, having removed the results previously included following partitioning (L200-204).

Also please present the full results of these models in tables (in supplements is fine). The difference between within and among mother effects of egg size on chick mass are important for inferring causality (see Pick et al 2016 Am Nat).

Authors' Response:
We have added this table in supplementary materials (new Table S1).

4. Analysis of provisioning rate
The suggestion to log transform provisioning rate and properly adjust for observation period is based on trying model the underlying process, rather than achieving normality of the residuals. This suggestion was based on this preprint (https://ecoevorxiv.org/repository/view/4531/), an updated version of which is in publication. The log transformation would be more meaningful, and I suspect would lead to stronger associations. As stated above, this transformation would make sense for both helper number and provisioning rate.

Authors' Response:
We agree that, while linear mixed models are quite robust to non-normality of the data (as the reviewer highlights above), there is merit in confirming that our findings are robust to modelling the underlying process. We therefore reran the analysis using a Poisson model and this confirmed our existing findings.

We read the suggested pre-print and followed its recommendations (Pick et al., 2023). We re-ran our initial maternal provisioning rate models using a Poison GLMM, with the daily count of maternal feeds as the response; and, clutch ID, group ID, season, maternal ID as random intercepts. We included the same fixed effects that we specified for the maternal provisioning rate model presented in the main text. Additionally, we included (log) video recording duration (i.e., the amount of time over which daily maternal feeds were counted) as an offset in the model. And following advice from L465-

467 of the pre-print, we also included an observation level random effect to account for overdispersion.

The results of this Poisson model confirm our existing findings using the Gaussian model. The number of female helpers negatively predicted maternal provisioning rates (now modelled as a count process, with maternal number of feeds as the response and (log) observation duration as a model offset); female helper number effect ± SE = -0.078 ± 0.030, $\chi^2_1 = 6.46$, p = 0.011. Also in line with our findings, there was no evidence of an effect of male helper number on maternal provisioning rates (male helper number effect [± SE] = -0.001 [±0.037], $\chi^2_1 = 0.001$, p = 0.982). We have included these additional results within the methods section of the main text, where we explain the modelling approach for maternal provisioning rate, to highlight that our findings are robust to taking a Poisson approach instead (L641-647). Here we also cite the published version of the highlighted pre-print (Pick et al., 2023). We did not log transform the helper number predictors as we did not *a priori* expect a logarithmic relationship between helper numbers and maternal provisioning. Avoiding log transformation of the helper number predictor also allows readers to utilise the effect sizes to think in a meaningful way about the biological effect of a helper on the response term (as previously requested by other reviewers).

Other Comments:
5. L55: None of references 4-8 refer to theory papers, so it seems a bit strange to me to base evolutionary predictions on them.

Authors' Response:
We have amended these references as requested to include papers with a stronger theory base.

6. L57 (and throughout): This might be personal preference, but 'per-offspring pre-natal maternal investment' seems a more natural way to phrase this, than 'maternal pre-natal investment per offspring'.

Authors' Response:
Thank you for this comment, which we gave full consideration. In the end, we decided to keep our current terminology here.

7. With regards to the use of the phrase 'maternal investment' - it might be worth referring to it as maternal expenditure, as it has been argued that maternal investment implies a known cost to the mother and benefit for the offspring, which is not shown here (and typically is very hard to demonstrate). For example it is defined as 'Any investment by the parent in an individual offspring that increases the offspring's survival and reproductive success at the cost of the parent's ability to invest in other current or future offspring' in Royle, Smiseth and Kolliker 2012 Evolution of Parental Care (Table 1.1). Parental expenditure would perhaps be most accurate. Parent care infers a known benefit to the offspring, which (with reference to a comment in my previous review) I don't think is known for wither of these maternal traits.

Authors' Response:
Thank you for this observation. We appreciate the potential merits of the distinction and so considered using 'investment' when referring to the theory and 'expenditure' when referring to our results. However, when attempting to implement this change it created terminological complexities that were, in our opinion, more problematic than beneficial. It would therefore be our preference to keep using the term investment.

8. The concept of 'additive post-natal care' seems important to the predictions but is not really explained anywhere. When I first read it I assumed that it meant that each additional helper

one average added the same amount of care again (i.e. the effect of helper number was additive), but I think this is wrong. The first reference to additive care (reference 8, Hatchwell 1999) defines it (in the abstract of Hatchwell 1999) as 'When parents maintain the same effort regardless of helper number, helper care is additive'. One justification for the utility of this system is that 'That only female helpers provide demonstrably additive post-natal care provides an unusual opportunity to distinguish the hypothesized pre-natal maternal responses to the availability of additive help' (L150). However, under the definition of Hatchwell 1999, its not known whether the care is additive until the effect on the mother post-natal are is known, and then in this system it would not be, as the mother reduces her care. Perhaps there is more subtlety in the definition than I understand, but it would be worth explaining this concept further.

Authors' Response:
Thank you for highlighting this. We have made adjustments to the text to clarify and emphasise the definition of this term in the Introduction (e.g., we now define it when outlining both of the focal hypotheses; L77-78 and L94-95; and remind the reader of its meaning again on its first use in the discussion; L354-355).

Both by our definition and its use *within* the Hatchwell 1999 paper (see below), 'additive' post-natal care refers to a scenario in which helper contributions increase the overall provision of post-natal care to offspring (L77-78 and L94-95 in our paper); so a scenario in which parents do not *fully* compensate for the contributions of helpers, such that helpers have a net-positive effect on the overall provision of post-natal care.

This definition is actually consistent with the statement in Hatchwell 1999's abstract, where the text states (as the reviewer rightly highlighted above) "*When parents maintain the same effort regardless of helper number, helper care is additive*". This statement is accurate (helper care is indeed additive in that specific scenario) but it isn't the *definition* of additive care, instead it just captures *one* of the scenarios in which helper care is additive. This becomes evident if one delves deeper into the text of the Hatchwell 1999 paper, where we find the statement "*Compensation by parents and additive care are not mutually exclusive. Both may occur in the same species depending on group size (e.g., Hatchwell and Russell 1996), or parents may only partially compensate for the care of a helper so that care is both additive and compensated*". Here one can see that Hatchwell's intended meaning for additive care was the same as our definition – helpers increase the overall provision of post-natal care to offspring (which may arise either if parents *do not* compensate at all for helper contributions [the scenario he mentions in the abstract] or if they *partially* compensate for helper contributions [the scenario he outlines in this later text]). This definition of additive care (as used by us and within the text of Hatchwell 1999) is also fairly standard in the field. For example, Savage et al 2013 (which we now cite alongside Hatchwell 1999 for this purpose) use additive care in the same way.

9. l 115 should be 'a meta-analysis'

Authors' Response:
Thank you for pointing this out. This has been amended (L117).

10. L 120-134: It seems strange not to reference Fortuna et al 2021 (reference 31), as they did exactly this within and between analysis with helper number in Sociable Weavers. It would be again appropriate to reference it on L143.

Authors' Response:
Thank you for this comment. We have included reference to this paper in L133 and L145, as suggested.

11. L 138 'We do so by testing for maternal plasticity in both pre-natal investment per offspring (egg volume; while accounting for effects of clutch size) and post-natal investment per offspring (maternal nestling provisioning rate) according to the availability of help.' It might be more consistent to write (maternal nestling provisioning rate accounting for brood size), as both clutch size and brood size are accounted for in order to look at per-offspring expenditure.

Authors' Response:
Thank you for his comment. We have amended the main text as suggested (L141-142).

12. L 200 As in my previous review, the relationship between egg size and chick survival has been previous tested in this system, and no statistical support found. It is disingenuous to cite this paper and suggest otherwise. In the response to my previous comment several possible confounders (such as not accounting for clutch size) were mentioned. From the introduction, it is not completely clear why the analysis on chick size is included. Presumably this is to demonstrate the importance of egg size. It seems to me that a model of chick survival would directly address this issue. If the argument is that it is a power issue, I would think that an analysis based on 490 eggs (or 906 in the full dataset) would be able to predict meaningful effect sizes, or at least a power analysis would show the minimum effect size that was able to be detected, and whether this minimum effect size was meaningful. There is also no analysis looking at the impact of provisioning rate on chick size or survival (as mention in my last review - comment 5), and this is an equally important part of the picture, if stages are being traded off against each other.

Authors' Response:
We appreciate that we do not capture the fitness consequences for offspring of variation in egg volume within this paper and now made changes to provide a more direct and nuanced treatment of this point in the discussion. First, we adjusted the text (L283-285) to clarify the biological significance of the demonstrated correlation between egg volume and hatchling mass, and to remove the text that previously highlighted at this point the potential for a link between egg volume and offspring survival mediated by hatchling mass. Second, we added a dedicated paragraph to directly address the issue of the potential fitness consequences of egg volume in a more nuanced way later in the discussion (L413-433). This paragraph highlights the likelihood of such fitness effects (given evidence to this effect in the literature), but also explicitly acknowledges that a previous study in this species did not detect an effect of the average egg volume in the clutch on offspring survival to fledging (addressing the perceived lack of transparency highlighted above). The text then notes too that in reality the relationship between egg volume and offspring fitness could well be complex (as selection for plasticity in egg investment is expected to stem from context-dependent payoffs) and that fitness consequences of egg investment could arise via effects on aspects of performance other than offspring survival to fledging (e.g., effects on the downstream performance of fledged young).

While we do not analyse the fitness consequences for offspring of variation in post-natal provisioning within the paper, we can confirm here that provisioning rate does indeed positively predict offspring survival to fledging in this species. Natural variation in the overall provisioning rate that broods receive significantly positively predicts the nestlings' probability of survival to fledging (binomial GLMM: effect of total provisioning rate on nestling survival ± S.E. = 0.076 ± 0.023; $\chi^2_1 = 11.32$, $p = 0.001$; n = 177 broods reared by 35 social groups over nine breeding seasons). This analysis utilises the binomial mixed-effects model of nestling survival to fledging whose findings are presented in Figure 3 of Capilla-Lasheras et al., (2021), but with the rainfall and helper number fixed-effect predictors removed and replaced with a predictor that captured the overall rate at which the brood was provisioned (specifically, the average of the available daily

measures of the overall rate at which the focal broods were fed; as determined for the analysis of overall provisioning rates presented in Figure 1 of Capilla-Lasheras et al., (2021)). The analysis was restricted to the 177 broods for which we had such provisioning rate information, and simultaneously controlled for the effects of variation in brood size (number of hatchlings) as a fixed effect predictor, with group ID, mother ID and breeding season ID fitted as random effects.

13. L 206 (and throughout) An effect size would be much more useful in the text than a chi squared stat - that and the p-value show the same thing, and the chisquared stats are in the tables. Presenting the effect sizes in the text allows comparison whilst reading, rather than constant reference back to a table (where they are also useful).

Authors' Response:
Thank you for this observation. We now include effect sizes in the results section to allow the reader comparison while reading. When we report effect sizes, we would prefer to keep the associated Chi squared and p-values in the text.

14. L214 (and 262) 'A significant difference between the effect sizes for the within- and among-mother components of female helper number would indicate that consistent differences in egg volume among mothers (other than those arising from the maternal plastic response to female helper number) also contribute significantly to the population-level relationship between female helper number and egg volume detected in Table 1'. This is confusing statistical significance with effect size. When the repeatability of helper number is low, the overall effect will be dominated by the within mother effect (as is seen here), and vice versa. A statistically significant difference just shows that the slopes are different, the contribution to the overall effect is to do with the relative amounts of within and between mother variation in the predictor.

Authors' Response:
Following your suggestions above and here, we have removed these statements (as we no longer include a formal comparison of within and among mother effects).

15. Tables - Is rainfall^1 refers to linear effect, I would suggest having rainfall and rainfall^2 instead - this is more standard notation. I was expecting footnotes to the table when I first looked at it.

Authors' Response:
Thank you for suggesting this clarification. We have amended main text and supplements as requested.

16. L 393 ' If, as here, mothers lay larger eggs when helped, controlling for variation in egg size could lead to the underestimation of helper effects on offspring, by factoring out helper effects that arise indirectly via maternal investment in the egg.'. I am not convinced that this is 'dangerous' approach - it simply helps separate direct and indirect effects - you would underestimate the total effects due to helpers, but correctly estimate the direct effects of helpers. It just addresses a different question, and requires researchers to to explicit about the assumptions of their model and what question their model answers.

Authors' Response:
We have amended the wording to clarify that underestimating the *total* helper effects is indeed the issue (L443) and have rephrased '…highlight a danger of this approach.' to '…highlight a potential problem with this approach.' (L441). This is an important point for us to make because the status quo in the cooperative breeding literature is an advocation of the need to factor out variation in offspring performance due to egg size to accurately measure *total* helper effects (a point first made in Russell et al. 2007).

17. L 481 It seems strange that measures provisioning rate per brood are averaged over, but not egg size per clutch?

> Authors' Response:
> We appreciate the point; we could have analysed maternal provisioning rate using the original 'repeated daily measures' of provisioning rate per brood (with Clutch ID as a random factor), rather than analysing the average of our original daily measures per brood (as we currently do). However, we can confirm that taking this alternative 'repeated daily measures' approach to the maternal provisioning analysis yields the same conclusions. We know this because we took the approach of analysing the repeated daily measures of maternal provisioning per brood (with brood ID as a random effect) when we employed the Poisson approach to modelling maternal provisioning rate in response to Comment 4 above (see there for details); this Poisson model yielded identical conclusions to those already presented within the paper.

18. L 484 How correlated were successive measures of provisioning rate?

> Authors' Response:
> Within broods, successive measures of maternal provisioning rates had a correlation coefficient of 0.46 (95%CI = [0.31, 0.59]). We have now included this information at this point within the methods (now L546-547).

19. L 524 'These interactive terms are included to specifically test whether the effect of helpers on egg volume is dependent on egg position or clutch size.'. In my last review I mentioned that the interactions lacked biological motivation. I still think this is the case. Why would the effects of helpers depend on egg position or clutch size? What hypothesis is being tested here?

> Authors' Response:
> We have further clarified this point in the introduction (L180-183 and L185-187). Less briefly… Helpers have the potential to differentially benefit offspring that are struggling due to deficits in maternal investment, and this plight is often predictably structured by laying order and/or clutch size (e.g., as eggs can differ in size or constituents with laying order or because first-laid eggs yield first-hatched chicks that dominate subsequent clutch-size dependent sibling competition; (Schwabl, 1993; Slagsvold et al., 1973; Fortuna et al. 2023). As such, we felt that it was important to allow for the possibility that mothers adjust egg volume to helper numbers in a different way across the laying order (e.g., under the differential allocation hypothesis, if helpers have little impact on the plight of the first-hatched chick [which will tend to hatch from the first-laid egg in this species], mothers might only be predicted to increase their investment in the second-laid egg when helped) or with variation in clutch size (e.g., similarly, under the differential allocation hypothesis, if helpers have little impact on the plight of chicks in one-egg clutches, mothers might only be predicted to increase their investment in the eggs of two-egg clutches when helped). Indeed, a recent paper has just reported that relationships between helper numbers and egg constituents do vary across the laying sequence in a related species (Fortuna et al., 2023). We therefore designed the statistical model of egg volume to account for egg position and clutch size effects and also allow for interactions between egg position and helper numbers and between clutch size and helper numbers.

20. There is no explanation as to relevance of egg position, although it is included in all the models. More importantly I don't understand why it is used as a criteria for exclusion of nearly half the available egg data (L540). It varies within a clutch, whereas the variables of interest (helper number) vary across clutches, and so I don't really see why egg position would affect these focal results. So, apart from it being nice to have a model with all explanatory variables in, I don't see the logic of MASSIVELY reducing the power of the analysis on behalf of this

variable. Please present results using data for all egg data, to show that the pattern still holds. This would also in part address the issue of how 'demanding' bivariate models might be with this data.

Authors' Response:
Thank you for highlighting the lack of clarity here. It was a priority to conduct the egg volume analysis with information available on egg position (laying order) in order to allow us to test for potentially important interactions between egg position and the focal helper effects of interest (see our reply to the previous comment) as well as to control for within-clutch variation in egg volume arising from egg position (the strongest predictor of egg volume; Table 1). The value of this approach is illustrated by recent evidence that the relationships between helper numbers and egg constituents can indeed vary across the laying sequence (Fortuna et al., 2023; a study that we now cite in the introduction [e.g., L187] and methods [L586]).

We also clarify that the egg volume data for which we lack egg position information has other correlated weaknesses (L602-604). For example, for methodological reasons, our measures of laid clutch size (a key predictor in the egg volume model, to allow for an egg volume / number trade-off) will also be less accurate for clutches without laying order data. This is because egg position information and accurate laid clutch size information both stem from conducting daily nest checks throughout the laying sequence (i.e., the longer the time interval between nest checks, the less likely it was that we had accurate laying order data, and the higher the probability that we missed laid eggs that were subsequently lost from the clutch before our next nest check). An analysis without the egg position predictor in the model would also leave any estimated clutch size effect confounded by variation in egg volume due to uncontrolled egg position effects (which our model shows are strong), because, for example, second-laid eggs can only occur in clutch sizes or two or more. Such confounding egg position effects would also complicate drawing biological inferences from our tested interaction between clutch size and helper numbers. As having accurate clutch size and egg position data was central to the questions of this study from the onset, we always carried out our analyses using the 490 eggs of known egg position and accurate clutch size, and never considered the extended dataset as it did not contain the necessary information.

However, following this comment, we have repeated the analysis with the full 906 eggs and confirmed that it leads to similar conclusions despite the limitations outlined above. To do this additional analysis, we first repeated the whole sliding window approach to characterise the appropriate time windows for assessment of the rainfall and heatwaves indices using this extended dataset. This recovered the same time windows of relevance for both indices as are already used in the existing analysis. We then re-ran the full model partitioning female and male helper number in their within- and among-mother components, including interactions between helper numbers and clutch size and using the random effect structure specified in the main text. This full model yielded an effect size for the effect of within-mother variation in female helper number on egg volume of +0.010 cm3 / female helper (for reference, the effect reported in the main text is +0.019 cm3 / female helper; Table 2). Additional analyses in which we aimed to explore the origin of this slight reduction in the overall effect size provided strong evidence for a positive effect of within-mother variation in female helper number on egg volume in clutches of one egg (effect size = +0.131, $\chi^2_1 = 20.89$, $p < 0.001$) and clutches of three eggs (effect size = +0.058, $\chi^2_1 = 7.26$, $p = 0.007$); but no evidence of an effect in clutches of two eggs (effect size = -0.002, $\chi^2_1 = 0.03$, $p = 0.855$). These contrasting effects of within-mother variation in female helper number across clutches of different sizes were reflected as a significant interaction between clutch size (as a categorical variable) and within-mother variation in female helper number ($\chi^2_2 = 6.06$, $p = 0.048$). The biological interpretation of this interaction is not straightforward within the expanded data set, as

egg position is unknown and it will confound clutch size (see above), but it does at least indicate that while the positive maternal plastic response to female helper number reported in the main text is still apparent within the expanded data set, its magnitude may depend upon clutch size and/or egg position.

As our existing analysis allows us to tease apart both the main and interactive effects of egg position and clutch size, allows us to test for biologically meaningful interactions of relevance to current literature (Fortuna et al 2023), uses the most accurate data, and is supported by the analysis of the expanded data set above, we have retained our existing approach within the paper. However, to complement that, we have now included the summary outlined above of the analysis of the expanded dataset within the supplementary materials (Supplementary Materials I), which we now refer to from the methods at the point at which we highlight our use of the dataset used for the main text analyses (L607-610). We also now highlight at that point in the methods, that these additional analyses of the expanded data set (i) support our main conclusion regarding a positive plastic maternal response in egg volume to female helper number, but (ii) highlight the possibility that the magnitude of that plastic response could vary with clutch size and/or egg position (being substantially larger in some contexts than that reported in the analyses presented in the main paper).

21. L 567 I suspect clutch size is very underdispersed with respect to a Poisson (it is notoriously so), I am surprised that this would fit well - simulating data Poisson with a mean of 2 results in a reasonable proportion of 4+ and that is with no additional variation between clutches/individual. Underdispersion would act to increase the false negative rate in these models.

Authors' Response:
Thank you for this suggestion. To confirm that our conclusions are robust to the potential existence of underdispersion, we re-ran the clutch size model presented in the main text using a zero-truncated negative binomial distribution in the R package glmmTMB (Brooks et al., 2017). This model provided no evidence for helper effects on clutch size. We further fitted this model using a Gaussian model in the R package lme4 (Bates et al., 2015) and, again, found no evidence for helper effects on clutch size. We have not included these additional results in the manuscript, but would be happy to consider it.

22. L 590 Why was breading season not included as random term in the analysis of number of clutches?

Authors' Response:
We do not include season as a random effect in this analysis because it perfectly covaries with rainfall. All mothers have the same rainfall predictor in a given breeding season (as it captures our estimate of the rainfall at the study site over that season). We have clarified this in the manuscript (L675-677).

23. It doesn't appear that maternal age is considered in the any analysis. Female reproductive output is known to change with age in many systems, and also conceivable that group size may also change with a dominant females age. Could age be driving any of the reported effects?

Authors' Response:
Thank you for this comment. We agree that this point is relevant and have now addressed it via some changes to the discussion (L323-325), which reference the following two studies in which we have verified that maternal age is not correlated with helper numbers in this system:

On page 2 of Capilla-Lasheras et al., (2021), we explain that maternal age effects are unlikely to confound helper effects in this species because maternal age and helper numbers aren't correlated:

> "Nor can these patterns be attributed to variation in maternal age, as maternal age is not correlated with female helper number [maternal age does not outperform a null (intercept only) model in predicting female helper number: ΔAIC (Akaike's information criterion) = +0.3; n = 124 breeding attempts by 25 mothers of known (±15 days) age]."

And on page 4 of Brown et al., (2021), we explain that (accordingly) parental age effects are unlikely to be confounded by helper effects for the same reasons:

> "Additional analyses suggest that these parental age relationships cannot be attributed to correlated variation in social group size (and hence helper number) […] (see electronic supplementary material, S3.1 and S3.2)".

References used in Authors' Response:

Bates, D., Machler, M., Bolker, B., & Walker, S. (2015). Fitting Linear Mixed-Effects Models Using lme4. *Journal of Statistical Software*, *67*, 1–48.

Brooks, M. E., Kristensen, K., van Benthem, K. J., Magnusson, A., Berg, C. W., Nielsen, A., Skaug, H. J., Maechler, M., & Bolker, B. M. (2017). glmmTMB Balances Speed and Flexibility Among Packages for Zero-inflated Generalized Linear Mixed Modeling. *The R Journal*, *9*(2), 378–400.

Brown, A. M., Wood, E. M., Capilla-lasheras, P., Harrison, X. A., & Young, A. J. (2021). Longitudinal evidence that older parents produce offspring with longer telomeres in a wild social bird. *Biology Letters*, *17*, 20210409.

Capilla-Lasheras, P., Harrison, X., Wood, E. M., Wilson, A. J., & Young, A. J. (2021). Altruistic bet hedging and the evolution of cooperation in a Kalahari bird. *Science Advances*, *7*, eabe8980.

Dixit, T., English, S., & Lukas, D. (2017). The relationship between egg size and helper number in cooperative breeders: a meta-analysis across species. *PeerJ*, *5*, e4028. https://doi.org/10.7717/peerj.4028

Fortuna, R., Paquet, M., Biard, C., Élard, L., Ferreira, A. C., Leroux-Coyaux, M., Parenteau, C., Silva, L. R., Théron, F., Covas, R., & Doutrelant, C. (2023). Egg components and offspring survival vary with group size and laying order in a cooperative breeder. *Oecologia*. https://doi.org/10.1007/s00442-023-05379-w

Hadfield, J. D. (2010). MCMC Methods for Multi-Response Generalized Linear Mixed Models in R: The MCMCglmm R package. *Journal of Statistical Software*, *33*(2), 1–22. https://doi.org/10.1002/ana.22635

Harrison, X., York, J. E., Cram, D. L., Hares, M. C., & Young, A. J. (2013). Complete reproductive skew within white-browed sparrow weaver groups despite outbreeding opportunities for subordinates of both sexes. *Behavioral Ecology and Sociobiology*, *67*(12), 1915–1929. https://doi.org/10.1007/s00265-013-1599-1

Harrison, X., York, J. E., & Young, A. J. (2014). Population genetic structure and direct observations reveal sex-reversed patterns of dispersal in a cooperative bird. *Molecular Ecology*, *23*, 5740–5755. https://doi.org/10.1111/mec.12978

Ludtke, O., Marsh, H. W., Robitzsh, A., Trautwein, U., Asparouhov, T., & Muthén, B. (2008). The Multilevel Latent Covariate Model: A New, More Reliable Approach to Group-Level Effects in Contextual Studies. *Psychological Methods*, *13*, 203–229. https://doi.org/10.1037/a0012869.supp

McAdam, A. G., Garant, D., & Wilson, A. J. (2014). The effects of others' genes: maternal and other indirect genetic effects. In A. Charmantier, dany Darant, & L. E. Kruuk (Eds.), *Quantitative Genetics in the Wild* (pp. 84–103). Oxford University Press. https://academic.oup.com/book/27921/chapter/204009906

Pick, J. L., Khwaja, N., Spence, M. A., Ihle, M., & Nakagawa, S. (2023). Counter culture: causes, extent and solutions of systematic bias in the analysis of behavioural counts. *PeerJ*, *11*, e15059. https://doi.org/10.7717/peerj.15059

Russell, A. F., Langmore, N. E., Cockburn, A., Astheimer, L. B., & Kilner, R. M. (2007). Reduced egg investment can conceal helper effects in cooperatively breeding birds. *Science*, *317*, 941–944.

Schielzeth, H., Dingemanse, N. J., Nakagawa, S., Westneat, D. F., Allegue, H., Teplitsky, C., Réale, D., Dochtermann, Ned. A., Garamszegi, L. Z., & Araya-Ajoy, Y. G. (2020). Robustness of linear mixed-effects models to violations of distributional assumptions. *Methods in Ecology and Evolution*, in press. https://doi.org/10.1111/2041-210x.13434

Schwabl, H. (1993). Yolk is a source of maternal testosterone for developing birds. *Proc. Natl. Acad. Sci. USA*, *90*, 11446–11450.

Slagsvold, T., Sandvik, J., Rofstad, G., Lorentsen, Y., & Husby, M. (1973). On the adaptive value of intraclutch egg-size variation in birds. *The Auk*, *101*, 685–697. https://academic.oup.com/auk/article/101/4/685/5191619
