# Supplementary material for: Mothers in a cooperatively breeding bird increase investment per offspring at the pre-natal stage when they will have more help with post-natal care
Source: PLoS Biol. 2023 Nov 9;21(11):e3002356. doi: 10.1371/journal.pbio.3002356 (PMC10635431; doi:10.1371/journal.pbio.3002356)
Supplement: Attachment — Submitted filename: Response to Reviewers - PBIOLOGY-D-22-02779R2.pdf [file pbio.3002356.s043.pdf]

Point-by-point response to editors' and reviewer's comments
PBIOLOGY-D-22-02779R2

Dear Dr Capilla-Lasheras,

Thank you for your patience while we considered your revised manuscript "Mothers front-load their investment to the egg stage when helped in a wild cooperative bird" for consideration as a Research Article at PLOS Biology. Your revised study has now been evaluated by the PLOS Biology editors, the Academic Editor and one of the original reviewers (reviewer #4).

IMPORTANT: You'll see that reviewer #4 still harbours some concerns. I discussed these with the Academic Editor, who sent me the following advice (somewhat edited), which I hope you will find useful:

"The authors have worked hard to deal with extensive reviewer comments and seem to have been diligent. However, I do suggest that you ask the authors to amend the paper in the light of the last set of comments of reviewer #4. I would make it clear that they don't have to make all the changes he required, but, in cases where they do not, they should put in writing why. This way there is a clear written record and it is possible to adjudicate as to the validity of their responses... The one change they should definitely make, however, is to include all the models they ran in the Supp Info. I am less certain about the full vs half dataset issues. In my experience authors usually have a good reason to exclude data. I would give the authors the benefit of the doubt here - but, again, ask them to be precise and put in writing - in the Supp Info or main text - exactly why they did not use the 'full dataset' or think it is not really suitable to draw the strongest conclusions."

In light of the reviews, which you will find at the end of this email, we are pleased to offer you the opportunity to address the [comments/remaining points] from the reviewers in a revision that we anticipate should not take you very long. We will then assess your revised manuscript and your response to the reviewers' comments with our Academic Editor aiming to avoid further rounds of peer-review, although might need to consult with the reviewers, depending on the nature of the revisions.

Authors' Response:
We thank the Editorial team for the invitation to respond to the reviewer comments and the guidance above on how we might best achieve this. As requested, we have responded in full to the reviewer's comments (see below for details) and addressed each of the Editorial requests above. This has entailed making the following revisions:

(i) including the additional analyses conducted in the previous round of replies within the main paper or supplements (just one was missing; see below for details);

(ii) including within the supplementary materials the full model output tables for all analyses conducted (the supplements now present 31 model output tables);

(iii) providing fuller and more conspicuous coverage of the outcomes of these additional analyses within the main paper, where they are addressed in full within the supplements (including the outcomes of the bivariate approach to estimating the maternal reaction norms and the analysis using the extended egg volume data set; see below for details);

(iv) ensuring, as requested by the Academic Editor, that we have clearly explained why the 'full dataset' analysis of egg volume is not suitable for drawing the strongest conclusions (while also reporting the outcomes of this full data set analysis in full within the supplements and signposting its findings within the main paper results section; see below for details);

(v) refining the bivariate modelling of the maternal reaction norms and the trade-off between maternal pre- and post-natal investment, which has left our results unchanged (see below for details).

We have sought throughout to embrace the full range of statistical approaches suggested by reviewers and have maximised the transparency of our reporting of the outcomes of these additional analyses, to ensure that readers are in a strong position to judge the evidence for themselves. To this end, we have also made available all of the R code and data used in our analyses (via a Github repository, which would get a DOI via Zenodo if the manuscript was accepted), to allow readers to also assess the evidence with their own preferred statistical methods, should they differ from the range of approaches already used here to verify our results.

We do hope our revised manuscript meets with your approval, and would be happy to make any further refinements should they be desired.

Best wishes and thank you again for taking the time to consider our manuscript.

Pablo Capilla-Lasheras
Andrew Young

REVIEWERS' COMMENTS:

Reviewer #4:
[identifies himself as Joel Pick]

The comments from my last review have generally been addressed. However, from what is presented between supplements and the response to reviewers, the results seem quite dependent on the model specification, and on the exact dataset used.

My purpose in suggesting some of these additional analyses is for them to act as sensitivity analyses. Statistical analysis is complex, and there are often many defensible ways to model something. Although I feel that some of the suggestions I have made have a clear root in statistical literature (e.g. the use of bivariate models versus within subject centering), if they give a similar answer than it is up to the authors to choose and defend their particular choice. These sensitivity analyses should then be clearly reported, and act to validate the findings presented. In this case, although many of the additional analyses suggested have been done, they are either not presented in the main text or supplements (just in the response to reviewers), or are presented in the supplements but somewhat disingenuously referred to in the main text (e.g. l 611-614). These additional analyses generally show results in the same direction, but in all cases the ones retained in the main text show a 'significant' result and the others non-significant effects. Now I am not particularly bothered by statistical significance at an arbitrary threshold, but this is the paradigm that is used in this manuscript to assess presence or absence of effects, and so this pattern of results being just below p=0.05 in the main text and above p=0.05 in sensitivity analyses does concern me. In some cases there is also a considerable shift in effect sizes between analyses (for example, when the 'full' dataset is used in the egg size analysis), but the same conclusions are presented.

> Authors’ Response: We thank the reviewer for their observations and have made further revisions to the analyses and manuscript to address them (see below for details).
>
> We have (i) included all of the ‘sensitivity’ analyses conducted in the previous round of replies in full, either within the main paper or supplementary materials, (ii) included the full model output tables for all of the additional analyses conducted, (iii) providing fuller and more conspicuous coverage of the outcomes of these additional analyses within the main paper, wherever they are addressed in full within the supplements (including the outcomes of the bivariate approach to estimating the maternal reaction norms and the analysis using the extended egg volume data set; see below for details), and (iv) refining the bivariate modelling of the maternal reaction norms and the trade-off between maternal pre- and post-natal investment, which has left our results unchanged (see below for details). We have also made available all of the R code and data used in our analyses to allow readers to assess the evidence with their own preferred statistical methods should they differ from the range of approaches already used here.
>
> The one analysis from the previous replies document that we didn’t include in the previous manuscript version was the bivariate version of our partitioning approach (though we did highlight that we’d be happy to include it, and had assumed that it would be available online anyway as part of our replies document). We have now included full coverage of this analysis within the supplementary materials (Supplementary Materials, Section H) and refer within the main paper both to the use of this method (methods lines 762-764) and to its outcome (main paper results section, lines 222-226 for egg volume & lines 271-274 for provisioning rate). We cover this bivariate analysis in more detail below in response to the reviewer’s suggestion focused on it.

Regarding our treatment of the expanded data set analysis (also covered in more detail below), our supplementary materials (Section J) make clear that there is still 'significant' evidence of plasticity when using this larger data set, but that this stems here from a significant interaction, in which the magnitude of the within-mother effect of female helper number (i.e., the plastic maternal response) depends upon clutch size. The estimated plastic maternal response is markedly stronger than that reported in the main paper in some clutch sizes and weaker in others (see below; Section J), but given weaknesses in data quality in this expanded data set (see below and Section J for details) we can't tell whether the interaction is driven by variation in clutch size or correlated differences in egg position, rendering biological interpretation difficult. We previously sought to highlight this outcome briefly within the main text while referring to full coverage of the findings within the supplementary materials. To ensure that this brevity in the main paper isn't interpreted as disingenuous, we have (i) expanded coverage of this extended analysis within the main text methods (lines 626-643) and (ii) highlighted the possibility of context dependence in the maternal reaction norm within the main paper results section (lines 239-244).

Follow up on previous comments:

1. Use of the 'full' dataset.
The change in effect size between the models run on different datasets is considerable. The effect size halves when the full dataset is used, and essentially becomes the same as the effect size seen in males in the reduced dataset (0.01 in females in full dataset versus 0.008 in males in reduced dataset(l 225)). However, this effect in females seems to be considered meaningful (l 611 - 'we also verified that our main conclusions are confirmed when the expanded dataset is analysed'), whereas this non-significant effect in males is described as there being no effect (e.g. l 309 'sparrow-weaver mothers appear to significantly adjust egg size according to female helper number and not male helper number').

In the response to reviewers, it is stated that this additional data is not suitable because clutch size is not known with high certainty. First, clutch size appears to have no affect on egg size, and is not affected by helper number, and there is no interaction effect on egg size between the two in the 'reduced' dataset. Second, it should be clear how often eggs are lost in this reduced dataset, and so what kind of uncertainty this would produce (although if clutch size doesn't correlate with helper number or egg size, it is not clear how important this uncertainty would be). Third, not finding a clutch on the first day of laying (and so the potential for missed eggs) doesn't seem to lead to the exclusion of nests from the analysis of clutch size (l 667).

I am also not convinced of how important it is to know the egg position for the analysis in question. Unlike clutch size, egg position does have a clear 'main' effect on egg size. However, it is not clear how this effect, when measured at the level of the observation (i.e. within clutches) should impact the within and between *mother* effects of helper number. As clutch size is not affected by helper number, there seems no reason why egg position should systematically vary with help number. I think dropping the egg position effect from the reduced dataset analysis would give some idea of any confounding effect - if the same change in effect size of the within mother effect of helper number is seen, this would indicate that there was something confounding here. If the result does not change, then I do not think there is a clear reason why the reduced dataset findings should be considered more reliable than a dataset double the size? A very quick look at this using the data provided on dryad shows that excluding egg position from this analysis does not change the within mother effect of helper number.

Also based on the description in the supplements, some aspects of egg position would be known for the additional nests, for example in nests with three eggs, when two were discovered on the same day, it would be known when the other egg was laid. There are several

ways of including missing data in analyses, and here it seems that data on over half the eggs would be known, and so these methods would be feasible. It is up to the authors to decide how to subset their data, but I am not convinced by the statistical justifications given to half the dataset.

Authors' Response: The expanded data set analysis (in which we explicitly highlighted the change in the mean effect size for female helper number mentioned above and then went on to probe its origins) yielded a significant interaction between within-mother variation in female helper number and clutch size, in which significant within-mother effects of female helper number were apparent for some clutch sizes but not others (see Supplementary materials, Section J). As such, the analysis provides evidence (i) of significant positive within-mother plasticity in egg size according to female helper number (the main finding from the paper), and (ii) suggests that the magnitude of this positive maternal reaction norm may depend upon clutch size, being larger than that reported in the main paper in some contexts (>3x larger for clutch size = 1 and >6x larger for clutch size = 3) and smaller in others (effectively zero for clutch size = 2). These findings support those in the main paper regarding the existence of maternal plasticity, but also highlight the possibility of context dependence in the magnitude of that plasticity.

We don't consider these results using the full data set more reliable (or more useful regarding biological insight) than those already in the main paper, principally because this full data set lacks information on egg position, leaving it impossible to account for effects of egg position, which is the strongest predictor of egg volume in the main paper analyses (Table 1) and will confound effects of clutch size (the term with which female helper number interacts in this analysis). We explain these points in the main text (methods lines 626-643; revised to provide more extensive coverage) when justifying the use of the higher quality data set, summarising the findings of the expanded data set analysis, and directing the reader to supplementary materials for full coverage of the analysis.

That said, we do agree that it is possible that the results when using the full data set do reflect biological reality, because the inability to allow for effects of egg position in this analysis (or tease them apart from effects of clutch size) need not necessarily render inaccurate the estimates of the maternal plastic response that this analysis yields. As such, we do now also direct the reader to the outcome of this analysis within the main paper results text (lines 239-244; pointing to full coverage in supplements), highlighting the possibility of some context-dependence in the maternal plastic response observed.

Finally, please present standard errors in the SM, not just lone effect sizes (and full model results would be preferable).

Authors' Response: As requested, we have now complemented the effect sizes and P values with the standard errors (Section J paragraph 3), and also provide the full model output tables for these analyses (Tables S26-S29). We include standard errors and/or 95% credible/confidence intervals in all model output tables.

2. Bivariate models.
Please provide the full results of the bivariate analyses in the supplements. In the responses to reviewers the correlations have been calculated and compared. As per the references in the last reviews I gave, the important thing to compare here are the *slopes* (covariance/variance in helper number at the same level). These slopes will also be directly comparable to the slopes estimated in the within/among subject centering approach. As such, it is difficult to compare the two approaches, and the comparison between within and among mother effects are not meaningful. As noted in the beginning of my review, the credible

intervals of the correlation estimates overlap 0 - presenting the derived slopes and their credible intervals would help interpret how large that overlap is in these analyses.

> Authors' Response: As requested, we've included the full coverage of the bivariate approach within the supplementary materials (Section H). Thank you for the suggestion here to make the effect size estimates from the partitioning and bivariate approaches comparable too. We have now implemented this approach and made changes to (i) highlight within the main paper results section that the two approaches yield comparable effect size estimates for the within-mother plastic reaction norms of interest (lines 222-226 for egg volume & lines 271-274 for provisioning rate), (ii) highlight within the methods that we conducted this bivariate analysis (pointing the reader to the supplementary materials for full information), and (iii) provided full coverage of the bivariate analysis methods and outcome (including the full model output tables) within the supplementary materials (Section H).
>
> To summarise, the partitioning approach employed throughout is expected to yield unbiased estimates of the within-mother slopes of interest here (Westneat et al., 2020); i.e., the maternal plasticity reaction norm). The partitioning approach estimates the effect size (± SE) for the within-mother reaction norm to female-helper number to be +0.019 (± 0.009) cm^3 / female helper ($p = 0.037$; Figure 2b; Table 2). The suggested bivariate approach is more data demanding but has the potential advantage of also yielding an unbiased estimate for the among-mother effect (which is not of particular interest here, hence it being unnecessary to employ this approach as our primary analytical method). The bivariate approach yields a similar estimate for the within-mother reaction norm to female-helper number (+0.015 cm^3 / female helper; as expected, given Westneat et al., 2020) with a 95% Credible Interval (-0.004, +0.033) that is principally positive too. That this credible interval spans zero at its limit (just as that for our within-mother correlation estimate did in our previous replies document; the two results being mathematically linked), is to be expected given the data-demanding nature of the bivariate approach and the already modest statistical significance of the reaction norm when using the partitioning approach ($p = 0.037$; see above). As the partitioning approach is already expected to yield unbiased estimates for the within-mother slopes (Westneat et al., 2020), and (accordingly) the bivariate approach yields a similar outcome, we see the two findings as complementary and appreciate the merit in the reader now having access to the outcomes of both within our results section.

3. The relationship between egg size and provisioning rate.
Thank you for adding this analysis. It is worth noting that there can be missing data in one or both of the responses in these models in MCMCglmm (it can just be included as NAs), and so the entire provisioning and egg size datasets can be used, even if they don't fully overlap. This will provide greater confidence to the between mother effects, and so may help estimate the correlations with more precision. I would also suggest changing the title in the supplements; 'Evidence for a trade-off between egg volume and maternal provisioning rate within mothers' is quite misleading as no trade-off between the two is detected. Also please present the full results of the model in a table.

> Authors' Response: Thank you for this suggestion. We have adjusted the implementation of the bivariate model as suggested, to include 'NA' data from each response variable (Supplementary Materials, Section I). Doing so has left the findings and conclusions from this model unchanged.
>
> To our knowledge, fitting the suggested model using MCMCglmm is not possible (because NA appear in the fixed effect matrix when a given response term is also NA). However, the suggested model can be fitted using the brms package (Bürkner, 2017)

and so we used this package to implement the suggestion. The results remain very similar to the previous analysis, revealing no evidence of a trade-off between maternal pre- and post-natal investment. Specifically, covariance within-mothers between egg volume and maternal provisioning rate was estimated to be 0.018 (95% CrI = -0.058, 0.097) in the initial model without helper numbers included as fixed effects (Table S24) and 0.032 (95% CrI = -0.042, 0.112) in the subsequent model including helper numbers as fixed effects (Table S25). We have updated the relevant methods and results within Supplementary Materials Section I to reflect this approach and its outcomes.

As requested, we have also (i) changed the title of the supplementary section (now: 'Seeking evidence of a trade-off between egg volume and maternal provisioning rate'; Supplementary materials, Section I) and (ii) included the full model output tables for this bivariate analysis (Tables S24 & S25).

Other comments:

l 234 - please present full model results in the supplements.

Authors' Response: As requested, we have now provided the full model output tables for all of the models referred to in the main text or supplementary materials (the supplements now feature 31 full model output tables).

l 614 'and could be substantially larger in some contexts than that reported in in the main paper).' Or substantially smaller!! On average they are smaller, and >70% nests (based on provided data) have a clutch size of 2, in which there is no within female effect in this extended analysis.

Authors' Response: Yes, we appreciate the point and have revised the main text to capture this outcome in more detail (lines 635-638). While the plastic response is estimated to be larger than that reported in the main paper for clutch sizes of 1 and 3 (see above) it is indeed estimated to be smaller for the clutch size of 2.

l 679 'Breeding season was not included as a random intercept in this analysis as it perfectly covaries with rainfall (i.e., all mothers were exposed to the same amount of rainfall per breeding season).'
I do not understand this argument, and I don' think it has any statistical basis. Any non-independence in the dataset needs to be modelled, otherwise there is a risk of underestimating standard errors. There are many parts of this analysis where a covariate is included that varies at the same level as a random effect. For example random effects of clutch ID are modelled in the same model that includes clutch size. Indeed, breeding season needs to be included in part because you are modelling something that varies at that level - otherwise you may appear to be over confident (reduced standard errors) in the estimates of rainfall. Perhaps what is meant is that rainfall explains all the among breeding season variation in the number of clutches? If so I think it would be clearer to describe in a different way, and I still don't see why breeding season wouldn't be included, even if it explained 0 variance.

Authors' Response: As requested, we have now included breeding season as a random effect in this analysis, which has left the relevant findings and conclusions unchanged. As in our previous version, this analysis reveals that neither female nor male helper number predict the number of clutches that mothers lay per year; findings reflected in the revised full model tables for these analyses (Table S18 and Table S19). As expected, the effect of the breeding season's rainfall becomes non-significant following the inclusion of breeding season as a random effect, but this result was of no consequence for our findings.

Tables 1-4 It would be worth keeping the order of male and female helper effects consistent

> Authors' Response: Thank you for this observation. We have made the requested change.

l301 in SM - Should it be 'bivariate' instead of 'binomial'?

> Authors' Response: Indeed. Thank you for catching this. We have now corrected it.

Generally, full results of models for all analyses in the main text and supplements need to be presented in the supplements.

> Authors' Response: As requested, we have now provided the full model output tables for all of the analyses within the supplementary materials.

Joel Pick

References

Bürkner, P.-C. (2017). brms: An R Package for Bayesian Multilevel Models Using Stan. *Journal of Statistical Software*, *10*(1), 1–28. https://doi.org/10.18637/jss.v080.i01

Westneat, D. F., Araya-Ajoy, Y. G., Allegue, H., Class, B., Dingemanse, N., Dochtermann, N., Garamszegi, L. Z., Martin, J. G. A., Nakagawa, S., Réale, D., & Schielzeth, H. (2020). Collision between biological process and statistical analysis revealed by mean-centering. *Journal of Animal Ecology*, *89*, 2813–2824. https://doi.org/10.1111/1365-2656.13360
